# Potential Precursors for Terminal Methylidene Rare‐Earth‐Metal Complexes Supported by a Superbulky Tris(pyrazolyl)borato Ligand

**DOI:** 10.1002/chem.201903606

**Published:** 2019-10-22

**Authors:** Verena M. Birkelbach, Renita Thim, Christoph Stuhl, Cäcilia Maichle‐Mössmer, Reiner Anwander

**Affiliations:** ^1^ Institut für Anorganische Chemie Eberhard Karls Universität Tübingen Auf der Morgenstelle 18 72076 Tübingen Germany

**Keywords:** alkyls, halides, heteroleptic compounds, rare-earth metals, scorpionates

## Abstract

A series of solvent‐free heteroleptic terminal rare‐earth‐metal alkyl complexes stabilized by a superbulky tris(pyrazolyl)borato ligand with the general formula [Tp^*t*Bu,Me^LnMeR] have been synthesized and fully characterized. Treatment of the heterobimetallic mixed methyl/tetramethylaluminate compounds [Tp^*t*Bu,Me^LnMe(AlMe_4_)] (Ln=Y, Lu) with two equivalents of the mild halogenido transfer reagents SiMe_3_X (X=Cl, I) gave [Tp^*t*Bu,Me^LnX_2_] in high yields. The addition of only one equivalent of SiMe_3_Cl to [Tp^*t*Bu,Me^LuMe(AlMe_4_)] selectively afforded the desired mixed methyl/chloride complex [Tp^*t*Bu,Me^LuMeCl]. Further reactivity studies of [Tp^*t*Bu,Me^LuMeCl] with LiR or KR (R=CH_2_Ph, CH_2_SiMe_3_) through salt metathesis led to the monomeric mixed‐alkyl derivatives [Tp^*t*Bu,Me^LuMe(CH_2_SiMe_3_)] and [Tp^*t*Bu,Me^LuMe(CH_2_Ph)], respectively, in good yields. The SiMe_4_ elimination protocols were also applicable when using SiMe_3_X featuring more weakly coordinating moieties (here X=OTf, NTf_2_). X‐ray structure analyses of this diverse set of new [Tp^*t*Bu,Me^LnMeR/X] compounds were performed to reveal any electronic and steric effects of the varying monoanionic ligands R and X, including exact cone‐angle calculations of the tridentate tris(pyrazolyl)borato ligand. Deeper insights into the reactivity of these potential precursors for terminal alkylidene rare‐earth‐metal complexes were gained through NMR spectroscopic studies.

## Graphical abstract

**There's a bulky scorpion in the way**: Although the scorpionate ligand Tp^*t*Bu,Me^ (hydrotris(3‐*tert*‐butyl‐5‐methylpyrazolyl)borato) facilitates the formation of unprecedented discrete heteroleptic alkyl complexes of the type [Tp^*t*Bu,Me^LnMeR] (Ln=Y, Lu; R=CH_2_Ph, CH_2_SiMe_3_), conversion into alkylidene derivatives is impeded due to lack of reactivity or ancillary ligand degradation.

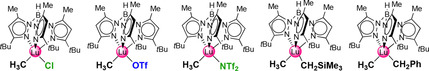

## Introduction

**Scheme 1:**
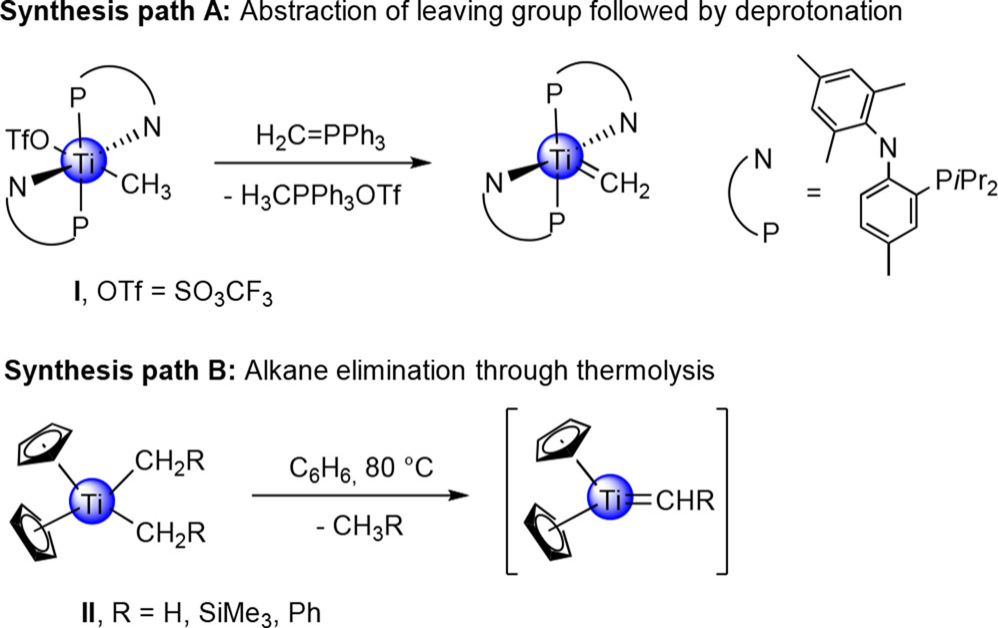
Synthesis approaches in titanium alkylidene chemistry. **Path A** displays the formation of a terminal methylidene through abstraction of a weakly coordinating OTf group and introduction of the CH_2_ group utilizing the Wittig reagent. **Path B** shows the proposed intermediate for the thermolysis and alkane/toluene elimination of dialkyl titanocenes.

Terminal transition‐metal carbene and alkylidene complexes are of fundamental importance in organometallic chemistry/catalysis and organic synthesis.^[1]^ In contrast, discrete terminal rare‐earth‐metal (Ln) alkylidene complexes of the type LLn [=CR_2_] (R=H or hydrocarbon substituent; L=monoanionic ancillary ligand) have remained elusive,^[2]^ which is mainly attributed to the dominance of Ln−C ionic bonding and hence pronounced tendency for intermetallic bridging.^[3]^ Notwithstanding, such bridging alkylidene moieties were accessed in mixed methyl/methylidene,^[4]^ methyl/chloride,^[5]^ cubane‐like methylidene complexes,^[6]^ and the first four‐coordinate methandiide alkyl lutetium complex.^[7]^ Also, Lewis acid‐stabilized^[8]^ or pincer‐like rare‐earth‐metal alkylidene complexes^[9]^ have been reported. Recent advances in the latter areas are represented by the syntheses of the first bridged bis‐alkylidene scandium complex,^[10]^ a non‐pincer‐type monometallic phosphinoalkylidene scandium complex,^[11]^ and silyl‐thiophosphinoyl alkylidene as well as phosphinomethylidene rare‐earth‐metal compounds.^[12]^ By nature, higher‐valent transition‐metal chemistry draws upon alternative approaches to access terminal alkylidenes. For example, in 2017, Mindiola and co‐workers isolated the first terminal titanium methylidene complex [(PN)_2_Ti(=CH_2_)] by treating [(PN)_2_Ti(CH_3_)(OTf)] (**I**, PN=(*N*‐(2‐(diisopropylphosphino)‐4‐methylphenyl)‐2,4,6‐trimethylanilide)) with the Wittig reagent H_2_CPPh_3_. This protocol involves the abstraction of the weakly coordinating OTf group (OTf=trifluoromethanesulfonato, also triflato or SO_3_CF_3_) and formation of the reactive Ti=CH_2_ moiety (Scheme 1, path **A**).^[13]^ Another prominent example in transition‐metal methylidene chemistry is the reaction behavior of [Cp_2_Ti(CH_2_R)_2_] (**II**, Cp=C_5_H_5_, R=H, SiMe_3_, Ph) during thermolysis.^[14]^ Petasis et al. found this compound to be an olefination agent for carbonylic derivatives. Therefore, terminal alkylidenes [Cp_2_Ti(=CHR)] were proposed as reaction intermediates (Scheme 1, path **B**), similar to the effective methylenating species of the Tebbe reagent.^[15]^ Although Petasis et al. could not confirm their proposal by X‐ray diffraction analysis (neither did Tebbe et al.), methane elimination during thermolysis and further reactivity studies substantiated their proposal of an intermediate methylidene moiety. Additionally, mixed alkyl titanocenes, for example, [Cp_2_Ti(CH_3_)(CH_2_SiMe_3_)]^[14a]^ showed the ability of olefination during exposure to higher temperatures. Crucially, all the aforementioned titanium(IV) alkylidene chemistry proceeds at a relatively small Ti^IV^ center supported by two monoanionic stabilizing ligands. Only recently, Okuda and co‐workers reported on the structural elucidation of the anionic complex [Li(Me_3_TACD)Ti(CHSiMe_3_)(CH_2_SiMe_3_)_2_] (Me_3_TACD=1,4,7‐trimethyl‐1,4,7,10‐tetraazacyclododecane).^[16]^ Inspired by this transition‐metal alkylidene chemistry, and in particular that of titanium, our group investigated the feasibility of rare‐earth‐metal variants of Mindiola's and Petasis’ starting compounds, for example, [LLn(CH_3_)(OTf)] and [LLn(CH_3_)R] (R = alkyl, L = monoanionic ancillary ligand). Herein, we present different reaction schemes for the synthesis of the targeted heteroleptic complexes and further reactivity studies for their utilization in rare‐earth‐metal alkylidene chemistry.

## Results and Discussion

In search of potential precursors for terminal Ln^III^ alkylidene chemistry we focused on monomeric compounds [Tp^*t*Bu,Me^LnMe(AlMe_4_)] (Ln=Y, Lu)^[17]^ and [Tp^*t*Bu,Me^LuMe_2_]^[18]^ supported by the superbulky scorpionate ligand Tp^*t*Bu,Me^ (hydrotris(3‐*tert*‐butyl‐5‐methylpyrazolyl)borato).^[19]^ Importantly, Piers et al. and Takats and co‐workers reported similar complexes featuring [Tp^R,Me^Sc(CH_2_SiMe_3_)_2_(THF)_*x*_] (R=Me, *x=*1; R=*t*Bu, *x=*0),^[20]^ [Tp^Me,Me^Ln(CH_2_SiMe_3_)_2_(THF)] (Ln=Y, Nd, Sm, Yb, Lu),^[21]^ [Tp^*t*Bu,Me^Ln(CH_2_SiMe_3_)_2_] (Ln=Y, Yb, Lu),^[21]^ [Tp^*i*Pr,*i*Pr^Ln(CH_2_SiMe_3_)_2_(THF)] (Ln=Y, Lu)^[22]^ obtained from [Ln(CH_2_SiMe_3_)_3_(THF)_*x*_] either by protonolysis with HTp^R,R^ or reaction with TlTp^R,R^.

### “Half‐sandwich” triflate complexes

**Scheme 2:**
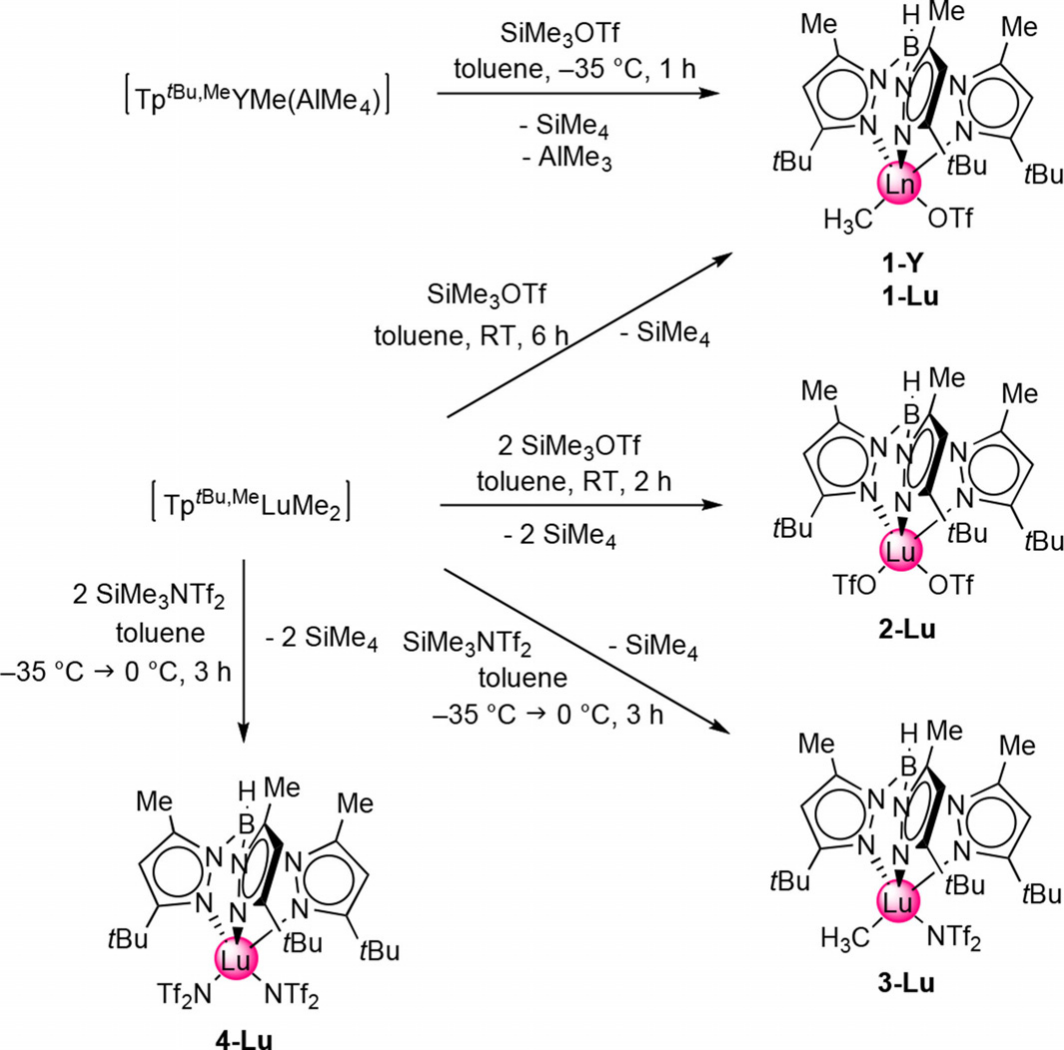
Synthesis pathways toward mixed methyl/triflate complexes [Tp^*t*Bu,Me^LnMe(OTf)] (Ln=Y, Lu), the mixed methyl/trifluoromethanesulfonimide complex [Tp^*t*Bu,Me^LuMe(NTf_2_)], bis(triflate) complex [Tp^*t*Bu,Me^Lu(OTf)_2_], and bis(triflimide) complex [Tp^*t*Bu,Me^Lu(NTf_2_)_2_].

In accordance to Scheme 1/path **A**/complex **I**, we anticipated the introduction of trifluoromethanesulfonato (OTf) or the even weaker coordinating trifluoromethansulfonimido (N(SO_2_CF_3_)_2_ or NTf_2_) ligands to be feasible through mild trimethylsilyl‐based transfer reagents SiMe_3_X (X=OTf, NTf_2_). Therefore, the scorpionate‐supported hydrocarbyl complexes [Tp^*t*Bu,Me^YMe(AlMe_4_)] and [Tp^*t*Bu,Me^LuMe_2_] were treated with one or two equivalents of SiMe_3_X, respectively, in toluene (Scheme 2).

In particular, given that the yttrium derivatives are extremely temperature sensitive, careful adjustment of the reaction conditions was required to afford complexes [Tp^*t*Bu,Me^LnMe(OTf)] (Ln=Y, **1‐Y**; Lu, **1‐Lu**), [Tp^*t*Bu,Me^Lu(OTf)_2_] (**2‐Lu**), [Tp^*t*Bu,Me^LnMe(NTf_2_)] (**3‐Lu**), and [Tp^*t*Bu,Me^Ln(NTf_2_)_2_] (**4‐Lu**). The ambient‐temperature ^1^H NMR spectra of the diamagnetic compounds **1**–**4** showed only one set of signals for the pyrazolyl groups of the Tp^*t*Bu,Me^ ligand with chemical shifts similar to those of the starting compounds (spectral data are presented in the Supporting Information). This indicates a highly fluxional behavior, which is in accordance with previous studies on complex [Tp^*t*Bu,Me^LuMe(AlMe_4_)].^[17]^ However, these previous studies also reported that similar complexes behave differently at lower temperatures, with the pyrazolyl rings revealing a 2:1 splitting in the ^1^H NMR spectra in accordance with the *C_s_* symmetry of these complexes in the solid state.^[17]^ For **1‐Lu** and **3‐Lu,** the Lu‐bound Me groups gave sharp singlets at *δ*=0.39 and 0.14 ppm, respectively.

**Figure 1:**
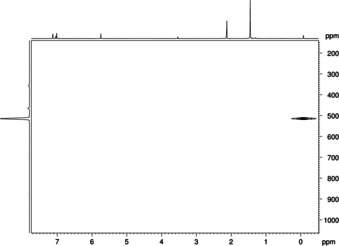
^1^H‐^89^Y HSQC NMR spectrum (24.5 MHz, [D_8_]toluene and a few drops of [D_8_]THF) of complex [Tp^*t*Bu,Me^YMe(OTf)] (**1‐Y**) at 0 °C.

The ambient‐temperature ^1^H NMR spectrum of **1‐Y** in C_6_D_6_ showed a broadened signal at *δ*=0.26 ppm for the terminal methyl moiety, not indicative of any Y−H coupling. To further investigate this behavior, a low‐temperature ^1^H NMR spectroscopy study was carried out (Figure S2 in the Supporting Information). Due to solubility issues in toluene at temperatures below 20 °C and rapid decomposition of complex **1‐Y** in THF, a few drops of [D_8_]THF were added to a precooled solution of **1‐Y** in [D_8_]toluene. Remarkably, the chosen NMR solvent “mixture” showed a strong influence on the chemical shift of the Y–Me moiety at low temperature, revealing a doublet at *δ*=−0.13 ppm (^2^ *J*(Y−H)=1.5 Hz) markedly shifted to higher fields compared with **1‐Y** in [D_6_]benzene (*δ*=0.26 ppm, Figure S1, Supporting Information). The ^1^H–^89^Y HSQC NMR spectrum of **1‐Y** at 0 °C shows a cross peak at *δ*=515 ppm on the ^89^Y NMR scale (Figure 1), which is shifted to higher field in comparison to precursor [Tp^*t*Bu,Me^YMe(AlMe_4_)] (*δ*=798 ppm).^[18]^ The ^13^C NMR spectra of the fluorine‐containing complexes **1‐Ln**, **2‐Lu**, **3‐Lu**, and **4‐Lu** showed one set of signals for the Tp^*t*Bu,Me^ ligand but ^13^C resonances of the CF_3_ groups could not be detected, which is consistent with already reported compounds.^[23]^ Notwithstanding, the presence of OTf and NTf_2_ moieties was unambiguously evidenced by ^19^F NMR spectroscopy revealing one sharp resonance at *δ*=−78.0, −78.1, −77.5, −77.9, and −76.9 ppm each for complexes **1‐Y**, **1‐Lu**, **2‐Lu**, **3‐Lu**, and **4‐Lu**, respectively.

**Figure 2:**
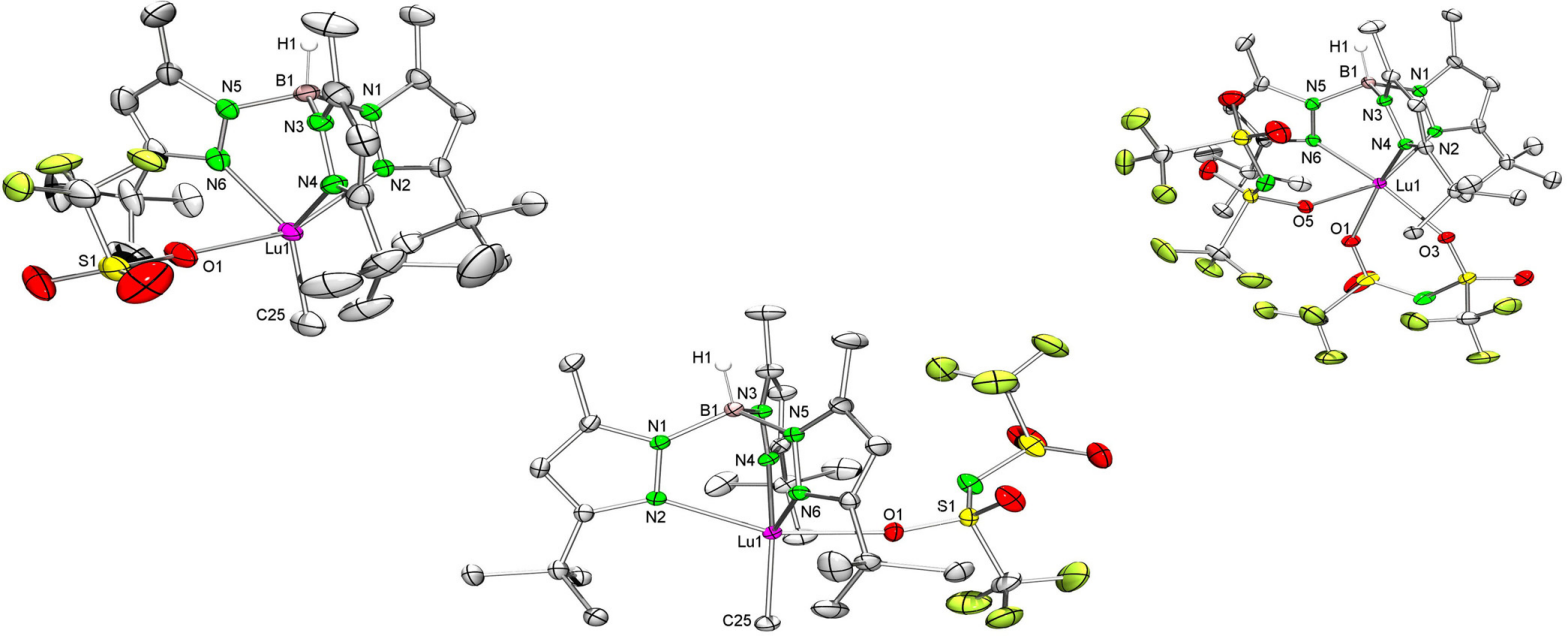
ORTEP representation of the molecular structure of **1‐Lu** (left), **3‐Lu** (middle), and **4‐Lu** (right) with atomic displacement parameters set at the 50 % level. Hydrogen atoms except for BH, toluene, and the disorder in one *t*Bu and the SO_3_CF_3_ group are omitted for clarity. Selected bond lengths are given in Table 1.

X‐ray crystallographic structure determinations were performed on **1‐Lu**, **3‐Lu**, and **4‐Lu** (Figure 2, Table 1). The fivefold‐coordinated lutetium centers of the methyl complexes [Tp^*t*Bu,Me^LuMeX] (X=OTf, **1‐Lu**; NTf_2_, **3‐Lu**) adopt a distorted trigonal‐bipyramidal coordination geometry. Moreover, the typically observed κ^3^ coordination of the ancillary ligand is adopted. In comparison with the reactant [Tp^*t*Bu,Me^LuMe_2_],^[24]^ the Lu−N(pz) (pz=pyrazolyl) bond lengths (2.339(2)–2.483(2) Å) of the Tp^*t*Bu,Me^ ligand are significantly shortened for **1‐Lu** (2.299(3)–2.376(3) Å) and **3‐Lu** (2.289(1)–2.328(1) Å); this could be attributable to the bulky electron‐withdrawing triflato moieties. As known from literature, OTf^−^ and NTf_2_^−^ moieties can coordinate in a monodentate, non‐bridging (N‐ or O‐wise, the latter was found for **1‐Lu** and **3‐Lu**) or in a bidentate, bridging fashion.^[25]^ The Lu−O1 distance for **1‐Lu** (2.191(3) Å) is significantly shorter than those reported before for eightfold‐coordinated [CpLu(OTf_2_)_2_(THF)_3_]^[26]^ (2.237(4), 2.213(4) Å) and [Lu(OTf)_3_(OPPh_3_)_4_]^[25a]^ (2.202(6), 2.232(5) Å) featuring likewise monodentate triflato ligands. Similarly, the Lu−O1 distance in bidentate eightfold‐coordinated complex [(bmpyr)Lu(NTf_2_)_4_]^[27]^ (bmpyr=1‐butyl‐1‐methylpyrrolidinium) (av. 2.30 Å) is elongated compared with that in **3‐Lu** (2.243(1) Å). The presence of the electron‐withdrawing triflato moieties implies also slightly shorter Lu−C(Me) distances of **1‐Lu** (2.327(4) Å) and **3‐Lu** (2.323(2) Å) than those in the precursor [Tp^*t*Bu,Me^LuMe_2_]^[24]^ (2.364(3)/2.375(2) Å). Despite several achievements on the structural characterization of various Tp‐supported Ln−OTf complexes,^[28]^ mixed Me/OTf and Me/NTf_2_ structural motifs have not yet been identified. So far, the structurally authenticated complexes comprise “sandwich complexes” exclusively, namely sevenfold‐coordinated [(Tp^Me,Me^)_2_Nd(OTf)] (Nd−O, 2.421(5) Å), sixfold‐coordinated [{(Tp^Me,Me^)_2_Yb}(OTf)], eightfold‐coordinated [(Tp^Me,Me^)_2_La(OTf)(CH_3_CN)] (La−O, 2.514(5) Å), and sevenfold‐coordinated [{(Tp^Me,Me^)_2_Nd(CH_3_CN)_2_}(OTf)]. All these complexes were synthesized through salt metathesis employing Ln(OTf)_3_ and KTp^Me,Me^, followed by exposure to donor molecules. Interestingly, complex [Tp^*t*Bu,Me^Ln(NTf_2_)_2_] (**4‐Lu**) is sixfold‐coordinated by Tp^*t*Bu,Me^ (κ^3^‐mode) and each one monodentate and bidentate NTf_2_ ligand (Figure 2, right). The Lu−O(triflato) distances range from 2.2213(1) to 2.2885(1) Å.

**Table 1:** Selected bond lengths [Å] of **1‐Lu**, **3‐Lu**, **4‐Lu**, **5‐Lu**, **6‐Lu**, **7‐Lu**, **8‐Lu**, **9‐Lu**.

	**1‐Lu** (X=Me, X′=O)	**3‐Lu** (X=Me, X′=O)	**4‐Lu** (X=X′=O)	**5‐Lu** (X=X′=Cl)	**6‐Lu** (X=X′=I)	**7‐Lu** (X=Me, X′=Cl)	**8‐Lu** (X=Me, X′=CH_2_)	**9‐Lu** (X=Me, X′=CH_2_)
Lu−N2	2.376(3)	2.376(3)	2.311(2)	2.391(6)	2.298(3)	2.313(9)	2.352(2)	2.310(2)
Lu−N4	2.299(3)	2.299(3)	2.3260(2)	2.306(3)	2.293(3)	2.413(1)	2.353(2)	2.323(2)
Lu−N6/N′	2.309(4)	2.309(4)	2.3108(2)	2.306(3)	2.378(3)	2.313(9)	2.487(2)	2.466(2)
Lu−X	2.327(4)	2.323(2)	2.2820(1)/2.2885(1)	2.4916(2)	2.8467(4)	2.393(1)	2.343(2)	2.349(3)
Lu−X′	2.191(3)	2.191(3)	2.2213(1)	2.494(1)	2.8987(4)	2.526(4)	2.372(2)	2.412(3)

To target the envisaged LnMeX to Ln=CH_2_ transformation (see Scheme 1/path **A**) complexes [Tp^*t*Bu,Me^LuMeX] (X=OTf, **1‐Lu**; NTf_2_, **3‐Lu)** were treated with one equivalent of H_2_CPPh_3_ in [D_6_]benzene. Unfortunately, no reactivity was observed at ambient temperature. Further heating the reaction mixture to 50 °C led to methane elimination through C−H‐bond activation involving the *t*Bu methyl groups of the ancillary ligand and the Lu−CH_3_ moiety, as observed previously.^[17]^ This intramolecular C−H‐bond activation could not be prevented by addition of N‐ or O‐donors like 4‐dimethylaminopyridine (DMAP) and tetrahydrofuran (THF).

### Generation of di(halogenido) and mixed methyl/halogenido and methyl/alkyl complexes

**Scheme 3:**
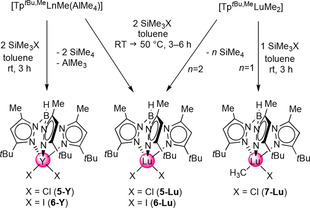
Synthesis pathways toward di(halide) complexes [Tp^*t*Bu,Me^LnX_2_] (Ln=Y, Lu; X=Cl, I) and the mixed methyl/halide complex [Tp^*t*Bu,Me^LuMeCl].

Further efforts to generate Ln^III^ alkylidenes led to the idea of targeting mixed methyl/alkyl (Me/R) complexes [Tp^*t*Bu,Me^LuMeR]. The latter might be convertible to the envisaged alkylidene species following a thermal or donor‐induced intramolecular elimination of either methane or the respective HR analog to Petasis (see Scheme 1/path **B**). Note that half‐sandwich complexes of the type [(C_5_Me_4_SiMe_3_)LnMe_2_]_3_ were previously shown to undergo such reactions affording tetrametallic cuboid clusters [(C_5_Me_4_SiMe_3_)Ln(*μ*_3_‐CH_2_)]_4_ (Ln=Tm, Lu).^[6]^ Preliminary NMR‐scale reactivity studies probing the olefination capability of [Tp^*t*Bu,Me^LuMe_2_] toward 9‐fluorenone at 50 °C (according to Petasis) indicated the exclusive formation of the respective alkoxide species. Therefore, to evade such preferential nucleophilic attack of the methyl moiety at the carbonyl functionality, the initial formation of an alkylidene species was envisaged. To provide a more versatile platform for further derivatization reactions, the above‐mentioned precursors [Tp^*t*Bu,Me^LnMe(AlMe_4_)] and [Tp^*t*Bu,Me^LuMe_2_] were treated with one equivalent of SiMe_3_X (here X=Cl, I) in toluene for the generation of mixed alkyl/halogenido compounds as depicted in Scheme 3.

Unfortunately, for yttrium and the combination Lu/I only the di(halogenido) derivatives [Tp^*t*Bu,Me^YCl_2_] (**5‐Y**), [Tp^*t*Bu,Me^YI_2_] (**6‐Y**), and [Tp^*t*Bu,Me^LuI_2_] (**6‐Lu**) could be isolated, evidencing extensive ligand redistribution. It is noteworthy that the synthesis and isolation of such di(halogenido) “half‐sandwich” complexes has been formerly assessed as problematic because of the occurrence of ligand redistribution reactions and B−N bond cleavage (formation of pyrazole adducts), in particular for complexes derived from Tp^Me,Me^.^[29]^ The few monomeric complexes [Tp^R,R^Ln(halogenido)_2_] authenticated by X‐ray structure analysis include THF adducts [(Tp^Me,Me^)LnCl_2_(THF)] and [(Tp^Me,Me^)_2_NdI_2_(THF), as well as N‐donor stabilized [(Tp^Me,Me^)_2_LnCl_2_(dmpzH)],^[30]^ [(Tp^Me,Me^)_2_YCl_2_(1,10‐phen)], and [(Tp^Me,Me^)_2_LaCl_2_(bipy)] (dmpzH: dimethylpyrazole, 1,10‐phen: 1,10‐phenanthroline, bipy: 2,2‘‐bipyridine).^[31]^

**Figure 3:**
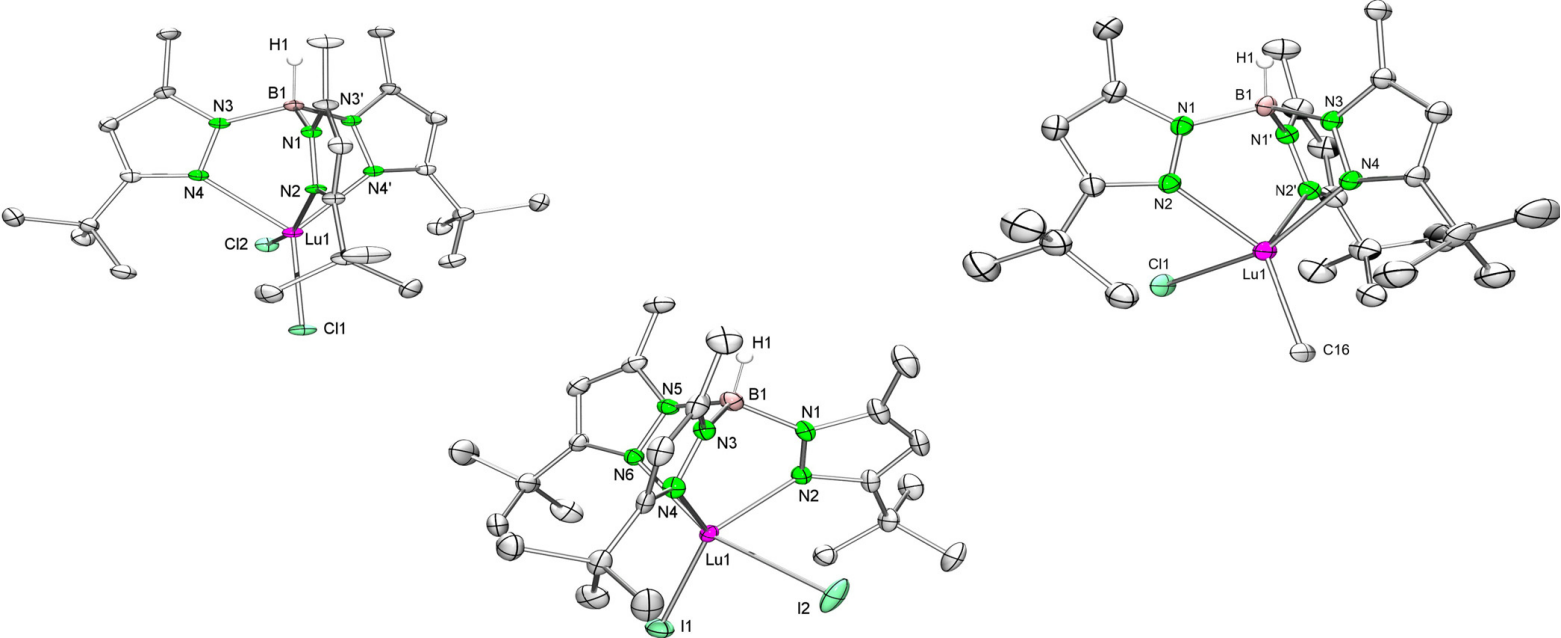
ORTEP representation of the molecular structures of **5‐Lu** (left), **6‐Lu** (middle), and **7‐Lu** (right) with atomic displacement parameters set at the 50 % level. Hydrogen atoms except for BH and solvent THF are omitted for clarity. Selected bond lengths are given in Table 1.

Much to our delight, the combination Lu/Cl gave the desired mixed methyl/chloride complex [Tp^*t*Bu,Me^LuMeCl] (**7‐Lu**), in addition to the di(chlorido) derivative [Tp^*t*Bu,Me^LuCl_2_] (**5‐Lu**, two‐equivalent reaction). All halide complexes exhibit low solubility which facilitated their isolation through crystallization (**5‐Y**, **6‐Y**, **6‐Lu**, **7‐Lu**) or precipitation (**5‐Lu**) from toluene solutions. Single crystals of **6‐Lu** and **7‐Lu** were grown from saturated toluene solutions at −35 °C, whereas **5‐Lu** was crystallized from THF at −35 °C. The ^1^H and ^13^C NMR spectroscopic data for all compounds clearly showed only one set of signals for the pyrazolyl groups of the ancillary ligand. In comparison with **1‐Lu** and **3‐Lu**, the proton NMR spectrum of **7‐Lu** shows a sharp singlet of the Lu−Me moiety located at *δ*=0.29 ppm, and hence shifted slightly to lower field. Overall, the Lu−N(pz) (pz=pyrazolyl) bond lengths in **5‐Lu**, **6‐Lu** and **7‐Lu** (Figure 3) are comparable to those found for **1‐Lu**, **3‐Lu**, and **4‐Lu**. The Lu−X distances in the di(halogenido) derivatives [Tp^*t*Bu,Me^LuCl_2_] (**5‐Lu**) and [Tp^*t*Bu,Me^LuI_2_] (**6‐Lu**) average 2.493 and 2.873 Å, respectively, reflecting the size of the halogenido anion. The Lu−C(methyl) bond length of 2.393(1) Å in **7‐Lu** is slightly longer than in [Tp^*t*Bu,Me^LuMe_2_]^[24]^ (2.364(3)/2.375(2) Å) and complexes **1‐Lu** and **3‐Lu** (see Table 1). Striking is the elongated Lu−Cl bond of 2.526(4) Å in **7‐Lu** compared with **5‐Lu**, apparently caused by the presence of the methyl ligand.

**Scheme 4:**
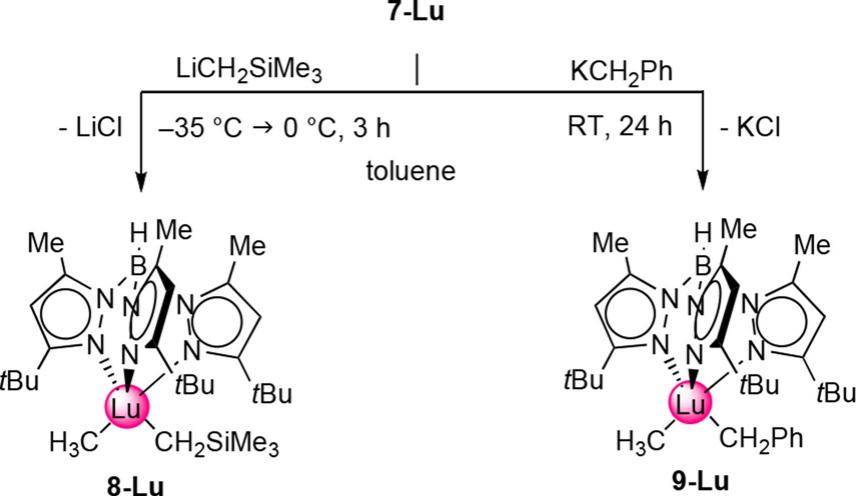
Salt‐metathesis conversion of methyl/halide complex [Tp^*t*Bu,Me^LuMeCl] (**7‐Lu**) to mixed methyl/alkyl compounds [Tp^*t*Bu,Me^LuMeR] (R=CH_2_SiMe_3_ (**8‐Lu**), CH_2_Ph (**9‐Lu**)).

Aiming at mixed methyl/alkyl compounds, the mixed methyl/chloride lutetium complex **7‐Lu** was examined in salt‐metathesis reactions with different alkali‐metal alkyls (Scheme 4). Due to the low solubility of **7‐Lu** in other nonpolar solvents and unintended C−H‐bond activation in donor solvents, all subsequent reactions were carried out in toluene.

The mixed alkyl complex [Tp^*t*Bu,Me^LuMe(CH_2_SiMe_3_)] (**8‐Lu**) was obtained by reacting **7‐Lu** with LiCH_2_SiMe_3_. Due to the thermal lability of **8‐Lu**, the reaction was performed at temperatures below 0 °C. Such low temperatures are also beneficial to the use of Li salts because conducting the involved metathesis reactions at ambient temperature favors the formation of LiTp^*t*Bu,Me^.^[20]^ In contrast, the mixed methyl/benzyl complex [Tp^*t*Bu,Me^LuMe(CH_2_Ph)] (**9‐Lu**) is thermally stable, but a prolonged reaction time is crucial when reacting **7‐Lu** with potassium benzyl. For both mixed bis(alkyl) complexes **8‐Lu** and **9‐Lu**, the ^1^H and ^13^C NMR spectra show only one set of signals for the pyrazolyl groups. The Ln‐bound methyl groups appeared as narrow singlets at *δ*=0.19 (**8‐Lu**) and 0.39 ppm (**9‐Lu**). In agreement with literature reports, the methylene moieties of the neosilyl and benzyl ligand feature distinctly shifted signals at *δ*=−0.71 and 1.63 ppm, respectively, attributable to a strong electronic influence of the SiMe_3_/Ph groups.

**Figure 4:**
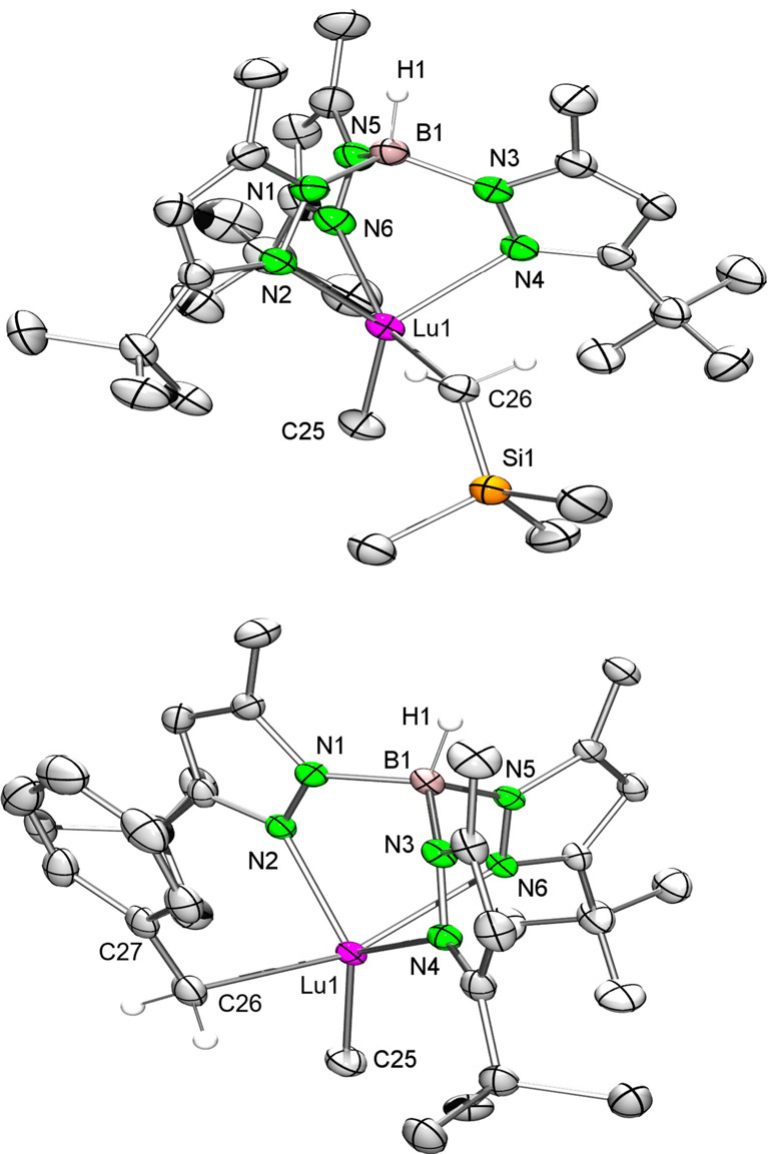
ORTEP representation of the molecular structures of **8‐Lu** (top) and **9‐Lu** (bottom) with atomic displacement parameters set at the 50 % level. Hydrogen atoms except for BH and CH_2_ are omitted for clarity. For **9‐Lu** the disorder in one *t*Bu group and toluene are omitted for clarity. Selected bond lengths are given in Table 1.

Complexes **8‐Lu** and **9‐Lu** were crystallized from saturated toluene solutions at −35 °C and their solid‐state structures analyzed by X‐ray crystallography (Figure 4). As commonly observed for Ln^III^−Tp^*t*Bu,Me^ complexes with coordination number 5, both complexes adopt a distorted trigonal‐bipyramidal geometry. The pyrazolyl nitrogen atoms N2 and N4 and the methyl carbon C25 reside in the equatorial plane, whereas the methylene carbon atom C26 and the pyrazolyl nitrogen atom N6 occupy the axial positions. In comparison with complexes **1‐Lu** and **3‐Lu** the Lu−N(pz) bond lengths are slightly elongated for the mixed alkyl compounds **8‐Lu** (2.352(2)–2.487(2) Å) and **9‐Lu** (2.310(2)–2.466(2) Å).

Furthermore, the Lu−C(Me) distances of **8‐Lu** (2.343(2) Å) and **9‐Lu** (2.349(3) Å) lie in between those of **1‐Lu**/**3‐Lu** and **7‐Lu** (Table 1). In accordance with literature, the Lu−C(neosilyl) bond length of **8‐Lu** of 2.372(2) Å is in the same range as detected for Lu(CH_2_SiMe_3_)_3_(THF)_2_ ^[32]^ (2.314(18)–2.344(18) Å) and Tp^Me,Me^Lu(CH_2_SiMe_3_)_2_(THF) (2.373(2)–2.379(2) Å).^[21]^ Other rare solid‐state structures of monomeric but heteroleptic Tp‐based rare‐earth‐metal complexes as [Tp^R,R^Ln(Danip)(CH_2_SiMe_3_)] (R=Me or R=H, Ln=Yb, Danip=2,6‐di(*o*‐anisol)phenyl)) display similar bond lengths (Yb−C_*ipso*_: 2.414(3)–2.438(4)/2.402(4)–2.435(5) Å; Yb−C(neosilyl): 2.379(4)–2.392(4)/2.359(4)–2.368(4) Å) taking into account the metal‐ion size.^[33]^ The Lu−C(benzyl) bond length (2.412(3) Å) in **9‐Lu** matches that in Lu(CH_2_Ph)_3_(THF)_3_ ^[34]^ (2.404(7)–2.413(5) Å) and Lu(CH_2_Ph)_3_(THF)_2_ (2.380(3)–2.404(3) Å)^[35]^ but is slightly elongated compared with Cp*Lu(CH_2_Ph)_2_(THF) (2.378(2)–2.386(2) Å; Cp*=C_5_Me_5_).^[36]^ Furthermore, there is no significant secondary interaction between Lu1 and the *ipso* carbon atom C27 for **9‐Lu**, as suggested by the Lu1⋅⋅⋅C27 distance of 3.314 Å and the Lu‐C(CH_2_)‐C27 angle (114.3(2)°). For further comparison, complex [Tp^Me,Me^Y(CH_2_Ph)_2_(THF)] was obtained through salt metathesis from [Tp^Me,Me^YCl_2_(THF)_2_] and potassium benzyl (Y−C(CH_2_) 2.457(8) and 2.418(8) Å, Y‐CH_2_‐C_ipso_ 116.4(6) and 130.1(6)°).^[37]^

Next, we examined whether complexes **8‐Lu** and **9‐Lu** are capable of intramolecular deprotonation and alkane elimination (see Scheme 1/path **B**). As mentioned before, [Tp^*t*Bu,Me^LuMe(CH_2_SiMe_3_)] is temperature‐sensitive. After one day at ambient temperature, a solution of complex **8‐Lu** in [D_6_]benzene turned from colorless to yellow. Monitoring this behavior with ^1^H NMR spectroscopy revealed degradation of the ancillary ligand as indicated by different new pyrazolyl signals and methane evolution. Further attempts at generating alkylidenes through intramolecular deprotonation led to the use of N‐ or O‐donor molecules such as DMAP or THF, but failed for the same reasons. Although compound **9‐Lu** is stable in solution in [D_6_]benzene at ambient temperature, heating to 40 °C for 4 h also led to degradation of the ancillary ligand, as did the utilization of various donors. In accordance with other already reported degradation processes, we assume C−H‐bond activation of one *tert*‐butyl group or B−N bond cleavage to be responsible for the formation of multiple unidentified metal complexes in these reaction mixtures.[^29b^, ^38^] It is also noteworthy, that the di(chlorido) derivative [Tp^*t*Bu,Me^LuCl_2_] (**5‐Lu**) does not undergo any “Tebbe‐like” reaction with AlMe_3_ at ambient temperature, but leads to unidentified complicated reaction mixtures (ancillary ligand degradation) at elevated temperatures (*T*=50 °C).

In spite of these sobering findings, the successful isolation of mixed alkyl complexes **8‐Lu** and **9‐Lu** spurred our interest in the evaluation of the steric effects on the ancillary Tp^*t*Bu,Me^ ligand caused by the distinct triflato, halogenido, or alkyl co‐ligands. According to a method recently reported by our group, we calculated the exact ligand cone angles *Θ*° (the procedure is given in the Supporting Information).^[39]^ According to Allen and co‐workers, the term “exact” refers to the acute mathematical solution and does not reflect the accuracy of the input structure itself.^[39b]^ As a prerequisite for meaningful interpretations, the metal centers should have the same coordination number (CN, here 5) and the same overall charge. A general overview of the determined cone angles is summarized in Table 2.

**Table 2:** Overview of mathematically exact calculated cone angles *Θ*° [°] of selected Tp^*t*Bu,Me^LuMeX or Tp^*t*Bu,Me^LuMeR (see the Supporting Information for calculations).^[a]^

**1‐Lu**	**3‐Lu**	**6‐Lu**	**7‐Lu**	**8‐Lu**	**9‐Lu**
278.0 280.9	280.4	278.2	278.9	277.1	277.3

[a] directly determined from atomic positions.

The Tp^*t*Bu,Me^ ligand engages in an exclusive trigonal‐bipyramidal coordination geometry at the Lu complexes under study, and hence, very similar cone angles (*Θ*°=277.1 to 280.9° for CN=5) were calculated. For **1‐Lu**, two different cone angles are displayed due to the respective disorder in one *tert*‐butyl group. Nonetheless, the noticeable trend makes complexes with mixed alkyl co‐ligands the least sterically demanding, followed by the di(halide) complexes, whereas the weakly coordinating triflato or triflimido moieties allow for the largest cone angles. Another important finding is that the mathematically exact method determines cone angles distinctly higher than those reported for Tp^*t*Bu,Me^ complexes in the literature (*Θ*°=244°).^[40]^ Therefore, further efforts should be expended to build up a library for better comparison.

## Conclusions

Aiming at new synthesis protocols for terminal rare‐earth‐metal alkylidene complexes, we gained access to unprecedented mono‐tris(pyrazolyl)borate complexes. Following TMS‐elimination protocols by applying complexes [Tp^*t*Bu,Me^YMe(AlMe_4_)] and [Tp^*t*Bu,Me^LuMe_2_] along with SiMe_3_X (X=OTf, NTf_2_), the superbulky ligand Tp^*t*Bu,Me^ supports the formation of mixed methyl triflate and mixed methyl triflimide complexes of yttrium and lutetium as new structural motif in rare‐earth‐metal chemistry. Moreover, similar reactions employing SiMe_3_X (X=Cl, I) afforded not only unsolvated di(halide) complexes Tp^*t*Bu,Me^LnX_2_ but also the mixed methyl/chloride complex [Tp^*t*Bu,Me^LuMeCl]. The latter gave efficient access to mixed alkyl complexes [Tp^*t*Bu,Me^LuMe(CH_2_SiMe_3_)] and [Tp^*t*Bu,Me^LuMe(CH_2_Ph)] through salt‐metathesis reactions with different alkali‐metal salts. Unfortunately, neither reactivity studies utilizing the Wittig reagent nor the attempted thermally‐induced intramolecular deprotonation afforded rare‐earth‐metal alkylidene compounds. It seems that terminal alkylidenes devoid of Lewis acid stabilization are not accessible/isolable in the presence of this very Tp^*t*Bu,Me^ ligand, which engages preferably in intramolecular B−N‐ and C−H‐bond activation processes. Ongoing research focuses on tripodal ancillary ligand systems which are less prone to degradation and C−H‐bond activation.

## Experimental Section

All operations were performed under rigorous exclusion of air and water by using standard Schlenk, high‐vacuum, and glovebox techniques (MBraun 200B; <0.1 ppm O_2_, <0.1 ppm H_2_O). Solvents were purified by using Grubbs‐type columns (MBraun SPS, solvent purification system) and stored inside a glovebox. [D_6_]Benzene and [D_8_]toluene were obtained from Sigma–Aldrich and degassed, [D_6_]benzene was dried over NaK alloy for two days and [D_8_]toluene was stored over Na. Both were filtered prior to use. [D_8_]THF was obtained from Sigma–Aldrich, stirred over NaK alloy, and distilled. SiMe_3_Cl, trimethylsilyl trifluoromethanesulfonate (Me_3_SiOTf), and (trimethylsilyl)methyllithium (LiCH_2_SiMe_3_) solutions were purchased from Sigma Aldrich, SiMe_3_I and N‐(trimethylsilyl)bis(trifluoromethanesulfonyl)imide (Me_3_SiNTf_2_) were purchased from ABCR and all chemicals were used as received. Potassium benzyl (KBn),^[41]^ [Tp^*t*Bu,Me^YMe(AlMe_4_)],^[17]^ [Tp^*t*Bu,Me^LuMe(AlMe_4_)],^[17]^ and [Tp^*t*Bu,Me^LuMe_2_]^[18]^ were synthesized according to literature procedures. The NMR spectra of air‐ and moisture‐sensitive compounds were recorded by using J. Young valve NMR tubes on a Bruker AVII+400 spectrometer (^1^H, 400.13; ^13^C, 100.61; ^19^F, 376.31 MHz), on a Bruker AVII+500 spectrometer (^1^H, 500.13; ^13^C, 125.76; ^89^Y, 24.51 MHz) and on a Bruker AVII+250 spectrometer (^1^H, 250.00; ^11^B, 80.21; ^13^C, 62.86 MHz). IR spectra were recorded on a Thermo Fisher Scientific NICOLET 6700 FTIR spectrometer using a DRIFT chamber with dry KBr/sample mixture and KBr windows; IR (DRIFT) data were converted by using the Kubelka–Munk refinement. Elemental analyses were performed on an Elementar Vario MICRO Cube.

### Synthesis and characterization

**[Tp** ^***t*****Bu,Me**^ **YMe(OTf)] (1‐Y)**: A chilled solution of Me_3_SiOTf (18.1 mg, 0.0814 mmol) in toluene (2 mL) was added to a precooled solution of [Tp^*t*Bu,Me^YMe(AlMe_4_)] (50.0 mg, 0.0814 mmol) in toluene (5 mL) at −20 °C. The reaction mixture was allowed to stir for 12 h at −20 °C. The solution was concentrated in vacuo and stored at −35 °C. Crystallization yielded compound **1‐Y** (30.0 mg, 0.0443 mmol, 54 %) as colorless crystals. ^1^H NMR (250 MHz, [D_6_]benzene, 25 °C): *δ*=5.56 (s, 3 H, 4‐pz‐*H*), 4.42 (v br d, ^1^ *J*(BH)=350 Hz,1 H, B*H*), 2.01 (s, 9 H, pz‐C*H*_3_), 1.36 (s, 27 H, pz‐C(C*H*_3_)_3_), 0.26 ppm (s, Y‐C*H*_3_). ^1^H NMR (500 MHz, [D_8_]toluene, 0 °C): *δ*=5.70 (s, 3 H, 4‐pz‐*H*), 4.52 (v br d, ^1^ *J*(BH)=355 Hz,1 H, B*H*), 2.07 (s, 9 H, pz‐C*H*_3_), 1.40 (s, 27 H, pz‐C(C*H*_3_)_3_), −0.13 ppm (d, ^2^ *J*(YH)=1.5 Hz, 3 H, Y‐C*H*_3_). ^13^C{^1^H} NMR (126 MHz, [D_8_]toluene, 0 °C): *δ*=164.7 (5‐pz‐*C*), 147.0 (3‐pz‐*C*), 104.4 (4‐pz‐*C*), 32.7 (pz‐*C*(CH_3_)_3_), 31.0 (pz‐C(*C*H_3_)_3_), 25.4 (d, ^2^ *J*(YC)=22.3 Hz, Y‐C*H*_3_)), 13.4 ppm (pz‐C(*C*H_3_)). ^13^C NMR resonances for the triflato groups were not detected. ^11^B{^1^H} NMR (80 MHz, [D_6_]benzene, 26 °C): *δ*=−8.6 ppm. ^19^F{^1^H} NMR (376 MHz, [D_6_]benzene, 26 °C): *δ*=−78.0 ppm. ^89^Y NMR (from ^1^H‐^89^Y HSQC, 25 MHz, [D_8_]toluene, 0 °C): *δ*=515 ppm. IR (KBr): $\tilde{\nu }$ =2964 (w), 2931 (w), 2883 (w), 2873 (w), 2822 (vw), 2577 (B‐H, vw), 2248 (vw), 2202 (vw), 2124 (vw), 1764 (vw), 1737 (vw), 1562 (vw), 1536 (w), 1518 (vw), 1501 (w), 1480 (w), 1462 (w), 1428 (w), 1342 (m), 1335 (m), 1282 (s), 1270 (s), 1228 (vs), 1204 (vs), 1171 (m), 1164 (m), 1136 (vw), 1097 (vw), 1053 (s), 1002 (vw), 995 (vw), 960 (vw), 940 (vw), 909 (vw), 892 (vw), 873 (vw), 860 (vw), 850 (vw), 837 (vw), 799 (w), 763 (w), 716 (m), 711 (m), 702 (m), 696 (m), 688 (m), 637 (vs), 586 (vw), 571 (vw), 550 (vw), 512 (w), 480 (vw), 469 (vw), 463 (vw), 431 (vw), 424 cm^−1^(vw); elemental analysis calcd (%) for C_26_H_43_BF_3_N_6_O_3_SY: C 46.17, H 6.41, N 12.42; found C 46.98, H 7.80, N 12.95. Due to the high S and F contents no better elemental analysis could be obtained.

**[Tp** ^***t*****Bu,Me**^ **LuMe(OTf)] (1‐Lu)**: A solution of Me_3_SiOTf (18.0 mg, 0.0814 mmol) in toluene (2 mL) was added to a solution of [Tp^*t*Bu,Me^LuMe_2_] (50.0 mg, 0.0788 mmol) in toluene (5 mL) at ambient temperature. The reaction mixture was allowed to stir for 2 h. The solution was concentrated in vacuo and stored at −35 °C. Crystallization yielded compound **1‐Lu** (48.0 mg, 0.0630 mmol, 80 %) as colorless crystals. ^1^H NMR (250 MHz, [D_6_]benzene, 26 °C): *δ*=5.64 (s, 3 H, 4‐pz‐*H*), 4.50 (v br d, ^1^ *J*(BH)=355 Hz,1 H, B*H*), 1.96 (s, 9 H, pz‐C*H*_3_), 1.44 (s, 27 H, pz‐C(C*H*_3_)_3_), 0.39 ppm (s, 3 H, Lu‐C*H*_3_). ^13^C{^1^H} NMR (63 MHz, [D_6_]benzene, 26 °C): *δ*=166.5 (5‐pz‐*C*), 147.9 (3‐pz‐*C*), 104.4 (4‐pz‐*C*), 36.6 (Lu‐C*H*_3_), 32.5 (pz‐*C*(CH_3_)_3_), 31.1 (pz‐C(*C*H_3_)_3_), 13.1 ppm (pz‐C(*C*H_3_)). ^13^C NMR resonances for the triflato groups could not be detected. ^11^B{^1^H} NMR (80 MHz, [D_6_]benzene, 26 °C): *δ*=−9.2 ppm. ^19^F{^1^H} NMR (376 MHz, [D_6_]benzene, 26 °C): *δ*=−78.1 ppm. IR (KBr): $\tilde{\nu }$ =2964 (m), 2931 (w), 2910 (w), 2887 (vw), 2866 (vw), 2558 (vw, B‐H), 1540 (m), 1477 (w), 1464 (w), 1433 (m), 1382 (vw), 1365 (m), 1351 (s), 1336 (s), 1238 (s), 1206 (vs), 1186 (vs), 1141 (vw), 1070 (m), 1062 (m), 1030 vs), 1010 (w), 989 (vw), 848 (vw), 840 (vw), 822 (vw), 804 (w), 789 (w), 765 (m), 678 (vw), 660 (vw), 648 (s), 587 (vw), 516 (w), 511 (w), 488 (vw), 413 cm^−1^ (m); elemental analysis calcd (%) for C_26_H_42_BF_3_LuN_6_O_3_S: C 40.96, H 5.68, N 11.02; found C 41.12, H 5.57, N 10.53.

**[Tp** ^***t*****Bu,Me**^ **Lu(OTf)_2_] (2‐Lu)**: A solution of Me_3_SiOTf (36.2 mg, 0.163 mmol) in toluene (2 mL) was added to a solution of [Tp^*t*Bu,Me^LuMe_2_] (50.0 mg, 0.0788 mmol) in toluene (5 mL). The reaction mixture was allowed to stir for 4 h at ambient temperature. The solution was concentrated in vacuo and stored at −35 °C. Crystallization yielded compound **2‐Lu** (50.0 mg, 0.0558 mmol, 71 %) as colorless crystals. ^1^H NMR (400 MHz, [D_6_]benzene, 26 °C): *δ*=5.51 (s, 3 H, 4‐pz‐*H*), 4.61 (v br d, ^1^ *J*(BH)=115 Hz, 1 H, B*H*), 1.87 (s, 9 H, pz‐C*H*_3_), 1.40 ppm (s, 27 H, pz‐C(C*H*_3_)_3_). ^13^C{^1^H} NMR (63 MHz, [D_6_]benzene, 26 °C): *δ*=167.1 (5‐pz‐*C*), 148.5 (3‐pz‐*C*), 104.8 (4‐pz‐*C*), 32.4 (pz‐*C*(CH_3_)_3_), 31.0 (pz‐C(*C*H_3_)_3_), 12.8 ppm (pz‐C(*C*H_3_)). ^13^C NMR resonances for the triflato groups were not detected. ^11^B{^1^H} NMR (80 MHz, [D_6_]benzene, 26 °C): *δ*=−8.3 ppm. ^19^F{^1^H} NMR (376 MHz, [D_6_]benzene, 26 °C): *δ*=−77.5 ppm. IR (KBr): $\tilde{\nu }$ =3138 (vw), 2963 (w), 2932 (vw), 2849 (vw), 2572 (vw, B‐H), 1538 (m), 1480 (w), 1467 (w), 1455 (w), 1434 (m), 1355 (vs.), 1350 (vs), 1290 (vw), 1240 (s), 1202 (vs), 1193 (vs), 1167 (s), 1132 (vw), 1076 (w), 1061 (w), 1021 (m), 1004 (vs), 859 (vw), 850 (vw), 839 (vw), 826 (vw), 817 (vw), 804 (w), 765 (w), 677 (vw), 661 (vw), 637 (vs), 589 (vw), 568 (vw), 524 (vw), 508 cm^−1^ (vw); elemental analysis calcd (%) for C_26_H_40_BF_6_LuN_6_O_6_S_2_: C 34.83, H 4.50, N 9.37; found C 34.70, H 4.52, N 9.40.

**[Tp** ^***t*****Bu,Me**^ **LuMe(NTf_2_)] (3‐Lu)**: A precooled solution of Me_3_SiNTf_2_ (56.0 mg, 0.158 mmol) in toluene (5 mL) was added to a precooled solution of [Tp^*t*Bu,Me^LuMe_2_] (100 mg, 0.158 mmol) in toluene (5 mL) at −35 °C. The reaction mixture was allowed to stir for 3 h at 0 °C. The solution was concentrated in vacuo and stored at −35 °C. Crystallization yielded compound **3‐Lu** (80.0 mg, 0.0895 mmol, 57 %) as colorless crystals. ^1^H NMR (250 MHz, [D_6_]benzene, 26 °C): *δ*=5.66 (s, 3 H, 4‐pz‐*H*), 4.55 (v br d, ^1^ *J*(BH)=355 Hz, 1 H, B*H*), 2.05 (s, 9 H, pz‐C*H*_3_), 1.38 (s, 27 H, pz‐C(C*H*_3_)_3_), 0.14 ppm (s, 3 H, Lu‐C*H*_3_). ^13^C{^1^H} NMR (63 MHz, [D_6_]benzene, 26 °C): *δ*=166.9 (5‐pz‐*C*), 148.8 (3‐pz‐*C*), 105.1 (4‐pz‐*C*), 35.7 (Lu‐C*H*_3_), 32.3 (pz‐*C*(CH_3_)_3_), 31.0 (pz‐C(*C*H_3_)_3_), 13.0 ppm (pz‐C(*C*H_3_)). ^13^C NMR resonances for the triflato groups were not detected. ^11^B{^1^H} NMR (80 MHz, [D_6_]benzene, 26 °C): *δ*=−8.3 ppm. ^19^F{^1^H} NMR (376 MHz, [D_6_]benzene, 26 °C): *δ*=−77.9 ppm. IR (KBr): $\tilde{\nu }$ =3138 (vw), 3026 (vw), 2968 (m), 2931 (w), 2913 (w), 2863 (w), 2569 (vw, B‐H), 1602 (vw), 1537 (m), 1393 (w), 1477 (m), 1465 (m), 1433 (m), 1367 (vs), 1352 (s), 1323 (s), 1208 (vs), 1190 (vs), 1161 (s), 1141 (s), 1122 (s), 1060 (vs), 1030 (m), 988 (w), 848 (vw), 817 (w), 801 (m), 759 (m), 741 (w), 728 (w), 694 (w), 675 (vw), 657 (w), 642 (m), 614 (m), 599 (w), 569 (w), 511 (m), 482 (vw), 465 (vw), 434 cm^−1^ (w); elemental analysis calcd (%) for C_27_H_43_BF_6_LuN_7_O_4_S_2_ *x* C_7_H_8_: C 41.43, H 5.21, N 9.95; found C 41.01, H 5.12, N 9.99.

**[Tp** ^***t*****Bu,Me**^ **Lu(NTf_2_)_2_] (4‐Lu)**: A precooled solution of Me_3_SiNTf_2_ (56.0 mg, 0.158 mmol) in toluene (5 mL) was added to a precooled solution of [Tp^*t*Bu,Me^LuMe_2_] (50.0 mg, 0.0788 mmol) in toluene (5 mL) at −35 °C. The reaction mixture was allowed to stir for 4 h at 0 °C. The solution was concentrated in vacuo and stored at −35 °C. Crystallization yielded compound **4‐Lu** (65.0 mg, 0.0561 mmol, 71 %) as colorless crystals. ^1^H NMR (250 MHz, [D_6_]benzene, 26 °C): *δ*=5.74 (s, 3 H, 4‐pz‐*H*), 4.54 (v br d, ^1^ *J*(BH)=340 Hz, 1 H, B*H*), 2.06 (s, 9 H, pz‐C*H*_3_), 1.31 ppm (s, 27 H, pz‐C(C*H*_3_)_3_). ^13^C{^1^H} NMR (63 MHz, [D_6_]benzene, 26 °C): *δ*=168.1 (5‐pz‐*C*), 150.5 (3‐pz‐*C*), 106.9 (4‐pz‐*C*), 32.3 (pz‐*C*(CH_3_)_3_), 30.9 (pz‐C(*C*H_3_)_3_), 13.3 ppm (pz‐C(*C*H_3_)). ^13^C NMR resonances for the triflato groups could not be detected. ^11^B{^1^H} NMR (80 MHz, [D_6_]benzene, 26 °C): *δ*=−7.6 ppm. ^19^F{^1^H} NMR (376 MHz, [D_6_]benzene, 26 °C): *δ*=−76.9 ppm. IR (KBr): $\tilde{\nu }$ =3149 (vw), 2974 (w), 2936 (w), 2873 (vw), 2569 (vw, B‐H), 1544 (m), 1482 (w), 1464 (w), 1422 (w), 1358 (s), 1338 (vs), 1324 (m), 1239 (vs), 1218 (vs), 1193 (vs),1134 (s), 1119 (s), 1100 (vs), 1055 (m), 1035 (m), 1017 (w), 982 (vw), 928 (vw), 847 (vw), 838 (vw), 824 (w), 806 (w), 767 (w), 743 (w), 681 (vw), 661 (w), 653 (m), 605 (s), 579 (m), 531 (vw), 512 (m), 441 (vw), 426 cm^−1^ (vw); elemental analysis calcd (%) for C_28_H_40_BF_12_LuN_6_O_8_S_4_: C 29.03, H 3.48, N 9.67; found C 30.12, H 3.49, N 9.27. Due to the high S and F contents no better elemental analysis could be obtained.

**[Tp** ^***t*****Bu,Me**^ **YCl_2_] (5‐Y)**: A solution of SiMe_3_Cl (18.0 mg, 0.166 mmol) in toluene (5 mL) was added to a solution of [Tp^*t*Bu,Me^YMe(AlMe_4_)] (50.0 mg, 0.0814 mmol) in toluene (5 mL) and stirred for 3 h at ambient temperature. The solution was concentrated in vacuo and stored at −35 °C. Crystallization yielded compound **5‐Y** (42.0 mg, 0.0720 mmol, 89 %) as colorless crystals. ^1^H NMR (250 MHz, [D_8_]toluene, 26 °C): *δ*=5.56 (s, 3 H, 4‐pz‐*H*), 4.47 (v br d, ^1^ *J*(BH)=140 Hz, 1 H, B*H*), 1.95 (s, 9 H, pz‐C*H*_3_), 1.50 ppm (s, 27 H, pz‐C(C*H*_3_)_3_). ^13^C{^1^H} NMR (63 MHz, [D_8_]toluene, 26 °C): *δ*=175.4 (5‐pz‐*C*), 147.1 (3‐pz‐*C*), 104.0 (4‐pz‐*C*), 32.6 (pz‐*C*(CH_3_)_3_), 31.5 (pz‐C(*C*H_3_)_3_), 13.1 ppm (pz‐C(*C*H_3_)). ^13^C{^1^H} NMR (63 MHz, [D_8_]THF, 26 °C): *δ*=166.4 (5‐pz‐*C*), 147.6 (3‐pz‐*C*), 105.7 (4‐pz‐*C*), 33.3 (pz‐*C*(CH_3_)_3_), 31.5 (pz‐C(*C*H_3_)_3_), 13.2 ppm (pz‐C(*C*H_3_)). ^11^B{^1^H} NMR (80 MHz, [D_8_]THF, 26 °C): *δ*=−7.9 ppm. IR (KBr): $\tilde{\nu }$ =2963 (s), 2928 (w), 2859 (w), 2577 (vw, B‐H), 1538 (vs), 1471 (w), 1463 (m), 1435 (s), 1382 (w), 1360 (m), 1346 (s), 1346 (s), 1332 (w), 1241 (w), 1192 (s), 1173 (vs), 1133 (vw), 1121 (vw), 1067 (m), 1064 (m), 1029 (m), 1014 (w), 989 (vw), 847 (vw), 810 (w), 804 (w), 787 (m), 777 (w), 765 (s), 729 (vw), 683 (vw), 677 (vw), 659 (vw), 645 (m), 515 cm^−1^ (vw); elemental analysis calcd (%) for C_24_H_40_BCl_2_N_6_Y: C 49.42, H 6.91, N 14.41; found C 49.01, H 6.99, N 13.74.

**[Tp** ^***t*****Bu,Me**^ **LuCl_2_] (5‐Lu)**: In a pressure tube a solution of SiMe_3_Cl (40.0 mg, 0.368 mmol) in toluene (5 mL) was added to a solution of [Tp^*t*Bu,Me^LuMe_2_] (100 mg, 0.158 mmol) in toluene (10 mL) and stirred for 6 h at 50 °C. The formed precipitate was allowed to settle, the supernatant was decanted and the solid washed with *n*‐hexane (3×2 mL). The precipitate was dried in vacuo to afford **5‐Lu** (60.0 mg, 0.0896 mmol, 57 %) as a white solid. Single crystals suitable for X‐ray diffraction could be obtained by crystallization from a saturated THF solution at −35 °C. ^1^H NMR (400 MHz, [D_8_]THF, 26 °C): *δ*=6.04 (s, 3 H, 4‐pz‐*H*), 4.83 (v br d, ^1^ *J*(BH)=135 Hz, 1 H, B*H*), 2.38 (s, 9 H, pz‐C*H*_3_), 1.49 ppm (s, 27 H, pz‐C(C*H*_3_)_3_). ^13^C{^1^H} NMR (63 MHz, [D_6_]benzene, 26 °C): *δ*=166.1 (5‐pz‐*C*), 147.0 (3‐pz‐*C*), 104.1 (4‐pz‐*C*), 32.6 (pz‐*C*(CH_3_)_3_), 31.0 (pz‐C(*C*H_3_)_3_), 13.0 ppm (pz‐C(*C*H_3_)). ^11^B{^1^H} NMR (80 MHz, [D_8_]THF, 26 °C): *δ*=−9.2 ppm. IR (KBr): $\tilde{\nu }$ =2961 (vs), 2906 (s), 2862 (s), 2550 (w, B‐H), 1539 (vs), 1476 (s), 1463 (s), 1424 (vs), 1380 (m), 1356 (vs), 1332 (m), 1295 (vw), 1241 (s), 1192 (vs.), 1176 (vs), 1070 (vs), 1028 (s), 1015 (s), 987 (m), 913 (w), 867 (m), 849 (m), 840 (s), 804 (s), 789 (s), 781 (s), 766 (vs), 731 (w), 658 (m), 644 (s), 515 cm^−1^ (w); elemental analysis calcd (%) for C_24_H_40_Bl_2_LuN_6_: C 43.07, H 6.02, N 12.56; found C 43.32, H 5.99, N 12.39.

**[Tp** ^***t*****Bu,Me**^ **YI_2_] (6‐Y)**: A solution of SiMe_3_I (33.0 mg, 0.165 mmol) in toluene (5 mL) was added to a solution of [Tp^*t*Bu,Me^YMe(AlMe_4_)] (50.0 mg, 0.0814 mmol) in toluene (5 mL) and stirred for 3 h. The solution was concentrated in vacuo and stored at −35 °C. Crystallization yielded compound **6‐Y** (52.0 mg, 0.0679 mmol, 84 %) as colorless crystals. ^1^H NMR (250 MHz, [D_8_]toluene, 26 °C): *δ*=5.55 (s, 3 H, 4‐pz‐*H*), 4.50 (v br d, ^1^ *J*(BH)=130 Hz, 1 H, B*H*), 1.95 (s, 9 H, pz‐C*H*_3_), 1.54 ppm (s, 27 H, pz‐C(C*H*_3_)_3_). ^13^C{^1^H} NMR (63 MHz, [D_8_]toluene, 26 °C): *δ*=166.6 (5‐pz‐*C*), 147.9 (3‐pz‐*C*), 104.5 (4‐pz‐*C*), 33.1 (pz‐*C*(CH_3_)_3_), 32.1 (pz‐C(*C*H_3_)_3_), 13.2 ppm (pz‐C(*C*H_3_)). ^11^B{^1^H} NMR (80 MHz, [D_8_]toluene, 26 °C): *δ*=−8.7 ppm. IR (KBr): $\tilde{\nu }$ =2964 (s), 2927 (w), 2863 (vw), 2562 (vw, B‐H), 1539 (vs), 1473 (m), 1456 (w), 1430 (vs), 1380 (w), 1364 (m), 1135 (vw), 1124 (vw), 1068 (m), 1061 (m), 1027 (m), 1014 (w), 985 (w), 846 (vw), 825 (vw), 802 (w), 799 (w), 764 (s), 729 (vw), 683 (vw), 674 (vw), 659 (w), 642 (m), 515 (vw), 472 (vw), 440 cm ^−1^ (vw); elemental analysis calcd (%) for C_24_H_40_BI_2_N_6_Y: C 37.62, H 5.26, N 10.97; found C 37.68, H 5.18, N 11.00.

**[Tp** ^***t*****Bu,Me**^ **LuI_2_] (6‐Lu)**: A solution of SiMe_3_I (47.0 mg, 0.235 mmol) in toluene (5 mL) was added to a solution of [Tp^*t*Bu,Me^LuMe_2_] (50.0 mg, 0.0788 mmol) in toluene (5 mL) and stirred for 3 h at ambient temperature. The solution was concentrated in vacuo and stored at −35 °C. Crystallization yielded compound **6‐Lu** (60.0 mg, 0.0704 mmol, 89 %) as colorless crystals. ^1^H NMR (250 MHz, [D_8_]toluene, 26 °C): *δ*=5.60 (s, 3 H, 4‐pz‐*H*), 4.48 (v br d, ^1^ *J*(BH)=135 Hz, 1 H, B*H*), 1.94 (s, 9 H, pz‐C*H*_3_), 1.56 ppm (s, 27 H, pz‐C(C*H*_3_)_3_). ^13^C{^1^H} NMR (101 MHz, [D_8_]toluene, 26 °C): *δ*=167.3 (5‐pz‐*C*), 148.1 (3‐pz‐*C*), 105.0 (4‐pz‐*C*), 32.9 (pz‐*C*(CH_3_)_3_), 31.8 (pz‐C(*C*H_3_)_3_), 12.9 ppm (pz‐C(*C*H_3_)). ^11^B{^1^H} NMR (80 MHz, [D_8_]toluene, 26 °C): *δ*=−7.6 ppm. IR (KBr): $\tilde{\nu }$ =3126 (vw), 2961 (vs), 2928 (w), 2906 (w), 2862 (w), 2553 (vw, B‐H), 1541 (vs), 1475 (m), 1463 (w), 1430 (vs), 1381 (w), 1354 (s), 1324 (w), 1242 (w), 1201 (m), 1191 (s), 1171 (vs), 1131 (m), 1065 (vs), 1030 (w), 1024 (w), 1015 (w), 984 (w), 846 (vw), 824 (vw), 804 (m), 794 (m), 762 (s), 729 (vw), 673 (vw), 656 (w), 642 (m), 472 (vw), 412 cm^−1^ (w); elemental analysis calcd (%) for C_24_H_40_BI_2_LuN_6_: C 33.83, H 4.73, N 9.86; found C 33.96, H 4.68, N 9.93.

**[Tp** ^***t*****Bu,Me**^ **LuMeCl] (7‐Lu)**: A solution of SiMe_3_Cl (34.2 mg, 0.315 mmol) in toluene (5 mL) was added to a solution of [Tp^*t*Bu,Me^LuMe_2_] (200.0 mg, 0.315 mmol) in toluene (10 mL) and stirred for 3 h at ambient temperature. The solvent was evaporated and the remaining white precipitate was washed with cold toluene (3×2 mL). The solid was dried in vacuo to afford **7‐Lu** (150 mg, 0.231 mmol, 73 %). Single crystals suitable for X‐ray diffraction could be obtained by crystallization from a saturated THF solution at −35 °C. ^1^H NMR (400 MHz, [D_8_]THF, 26 °C): *δ*=5.98 (s, 3 H, 4‐pz‐*H*), 4.75 (v br d, ^1^ *J*(BH)=135 Hz, 1 H, B*H*), 2.40 (s, 9 H, pz‐C*H*_3_), 1.48 (s, 27 H, pz‐C(C*H*_3_)_3_), −0.29 ppm (s, 3 H, Lu‐C*H*_3_). ^13^C{^1^H} NMR (63 MHz, [D_8_]THF, 26 °C): *δ*=166.0 (5‐pz‐*C*), 147.6 (3‐pz‐*C*), 104.7 (4‐pz‐*C*), 35.5 (Lu‐C*H*_3_), 32.9 (pz‐*C*(CH_3_)_3_), 31.0 (pz‐C(*C*H_3_)_3_), 13.1 ppm (pz‐C(*C*H_3_)). ^11^B{^1^H} NMR (80 MHz, [D_8_]THF, 26 °C): *δ*=−8.7 ppm. IR (KBr): $\tilde{\nu }$ =2963 (s), 2953 (s), 2931 (w), 2907 (m), 2861 (w), 2575 (vw, B‐H), 1540 (vs), 1474 (m), 1463 (m), 1435 (vs), 1381 (w), 1362 (s), 1351 (s), 1335 (w), 1242 (w), 1193 (s), 1172 (vs), 1123 (w), 1075 (s), 1063 (s), 1030 (m), 1014 (w), 987 (w), 849 (vw), 841 (w), 806 (m), 787 (vs), 777 (m), 765 (vs.), 729 (w), 694 (vw), 677 (w), 660 (w), 645 (s), 515 (w), 492 (vw), 442 (vw), 411 cm^−1^ (m); elemental analysis calcd (%) for C_25_H_43_BClLuN_6_: C 46.28, H 6.68, N 12.95; found C 45.70, H 6.42, N 12.66.

**[Tp** ^***t*****Bu,Me**^ **LuMe(CH_2_SiMe_3_)] (8‐Lu)**: A precooled solution of LiCH_2_SiMe_3_ (14.5 mg, 0.154 mmol) in toluene (5 mL) was added to a precooled solution of [Tp^*t*Bu,Me^LuMeCl] (100 mg, 0.154 mmol) in toluene (5 mL) at −35 °C. The reaction mixture was allowed to stir for 3 h at 0 °C. The precipitate was filtered off and the solution was concentrated in vacuo and stored at −35 °C. Crystallization yielded compound **8‐Lu** (56.0 mg, 0.0799 mmol, 52 %) as colorless crystals. ^1^H NMR (400 MHz, [D_6_]benzene, 26 °C): *δ*=5.65 (s, 3 H, 4‐pz‐*H*), 4.54 (v br d, ^1^ *J*(BH)=360 Hz, 1 H, B*H*), 2.06 (s, 9 H, pz‐C*H*_3_), 1.51 (s, 27 H, pz‐C(C*H*_3_)_3_), 0.23 (s, 18 H, SiC*H*_3_), 0.19 (s, 3 H, Lu‐C*H*_3_), −0.71 ppm (s, 2 H, C*H*_2_SiMe_3_). ^13^C{^1^H} NMR (63 MHz, [D_6_]benzene, 26 °C): *δ*=165.3 (5‐pz‐*C*), 146.7 (3‐pz‐*C*), 103.9 (4‐pz‐*C*), 37.6 (Lu‐C*H*_2_), 32.6 (pz‐*C*(CH_3_)_3_), 31.9 (Lu‐C*H*_3_), 31.4 (pz‐C(*C*H_3_)_3_), 13.2 (pz‐C(*C*H_3_)), 4.5 ppm (Si*Me*_3_). ^11^B{^1^H} NMR (80 MHz, [D_6_]benzene, 26 °C): *δ*=−8.2 ppm. ^29^Si{^1^H} dept45 NMR (50 MHz, [D_6_]benzene, 26 °C): *δ*=−0.3 ppm. IR (KBr): $\tilde{\nu }$ =2960 (vs), 2926 (s), 2866 (m), 2815 (vw), 2552 (vw, B‐H), 1540 (vs), 1463 (m), 1432 (s), 1379 (w), 1360 (s), 1334 (w), 1236 (m), 1205 (m), 1195 (m), 1175 (s), 1128 (w), 1071 (m), 1060 (m), 1025 (w), 1013 (w), 985 (w), 894 (w), 872 (s), 854 (m), 816 (w), 806 (w), 791 (m), 766 (m), 743 (w), 731 (w), 717 (w), 675 (vw), 663 (w), 645 (m), 521 (vw), 513 (vw), 473 (vw), 434 (vw), 421 (w), 404 cm^−1^ (m); elemental analysis calcd (%) for C_29_H_54_BLuN_6_Si: C 49.71, H 7.77, N 11.99; found C 49.73, H 7.65, N 11.73.

**[Tp** ^***t*****Bu,Me**^ **LuMe(CH_2_Ph)] (9‐Lu)**: A suspension of KCH_2_Ph (20.0 mg, 0.154 mmol) in toluene (5 mL) was added to a solution of [Tp^*t*Bu,Me^LuMeCl] (100 mg, 0.154 mmol) in toluene (5 mL) and stirred for 24 h at ambient temperature. The reaction mixture was filtered and the solution was concentrated in vacuo and stored at −35 °C. Crystallization yielded compound **9‐Lu** (49.0 mg, 0.0695 mmol, 45 %) as colorless crystals. ^1^H NMR (250 MHz, [D_6_]benzene, 26 °C): *δ*=6.95 (t, ^3^ *J*(HH)=15.5 Hz, 2 H, Ar‐*H*), 6.62 (t, ^3^ *J*(HH)=15.9 Hz, 1 H, Ar‐*H*), 6.35 (d, ^2^ *J*(HH)=7.6 Hz, 2 H, Ar‐*H*), 5.62 (s, 3 H, 4‐pz‐*H*), 4.52 (v br d, ^1^ *J*(BH)=147 Hz, 1 H, B*H*), 2.02 (s, 9 H, pz‐C*H*_3_), 1.63 (s, 2 H, C*H*_2_), 1.44 (s, 27 H, pz‐C(C*H*_3_)), 0.39 ppm (s, 3 H, Lu‐C*H*_3_). ^13^C{^1^H} NMR (101 MHz, [D_6_]benzene, 26 °C): *δ*=164.9 (5‐pz‐*C*), 154.3 (Ar‐*C*1), 147.0 (3‐pz‐*C*), 127.3 (Ar‐*C*2/*C*6), 124.5(Ar‐*C*3/*C*5), 117.1 (Ar‐*C*4), 103.8 (4‐pz‐*C*), 61.0 (Lu‐*C*H_2_), 38.2 (Lu‐*C*H_3_), 32.3 (pz‐*C*(CH_3_)_3_), 31.2 (pz‐C(*C*H_3_)_3_), 13.0 ppm (pz‐C(*C*H_3_)). ^11^B{^1^H} NMR (80 MHz, [D_6_]benzene, 26 °C): *δ*=−8.3 ppm. IR (KBr): $\tilde{\nu }$ =3054 (vw), 2999 (vw), 2963 (vs), 2926 (w), 2903 (m), 2864 (w), 2544 (vw, B‐H), 1589 (m), 1539 (vs), 1486 (s), 1473 (s), 1431 (vs), 1362 (s), 1356 (s), 1330 (w), 1242 (w), 1218 (m), 1203 (m), 1190 (s), 1164 (s), 1129 (m), 1069 (s), 1057 (m), 1025 (m), 1015 (vw), 984 (w), 929 (s), 864 (w), 848 (vw), 810 (m), 802 (m), 787 (m), 775 (w), 764 (s), 742 (m), 732 (s), 696 (s), 682 (vw), 675 (vw), 661 (vw), 643 (s), 521 (w), 510 (vw), 468 (w), 457 cm^−1^ (w); elemental analysis calcd (%) for C_32_H_50_BLuN_6_: C 54.55, H 7.15, N 11.93; found C 54.72, H 7.25, N 12.29.

### X‐ray crystallography and crystal structure determinations

Single crystals of **1‐Lu**, **3‐Lu**, **4‐Lu**, **5‐Lu**, **6‐Lu**, **7‐Lu**, **8‐Lu**, and **9‐Lu** were grown by standard techniques from saturated solutions in *n*‐hexane, toluene or THF at −35 °C as stated in the experimental section. Suitable crystals were collected in a glovebox and coated with Parabar 10312 (previously known as Paratone N, Hampton Research) and fixed on a nylon loop/glass fiber.

X‐ray data for compounds of **1‐Lu**, **3‐Lu**, **4‐Lu**, **5‐Lu**, **6‐Lu**, **7‐Lu**, **8‐Lu**, and **9‐Lu** were collected on a Bruker APEX II DUO instrument equipped with an IμS microfocus sealed tube and QUAZAR optics for MoK_α_ (*λ*=0.71073 Å) and CuK_α_ (*λ*=1.54184 Å) radiation. The data collection strategy was determined using COSMO^[42]^ employing ω‐scans. Raw data were processed using APEX^[43]^ and SAINT,^[44]^ corrections for absorption effects were applied using SADABS.^[45]^ The structures were solved by direct methods and refined against all data by full‐matrix least‐squares methods on F^2^ using SHELXTL^[46]^ and ShelXle.^[47]^ Disorder models were calculated using DSR, a program for refining structures in ShelXl.^[48]^ All graphics were produced employing ORTEP‐3^[49]^ and POV‐Ray.^[50]^ Further details of the refinement and crystallographic data are listed in Table S1 (Supporting Information) and in the CIF files. CCDC 1945695, 1945696, 1945697, 1945698, 1945699, 1945700, 1945701, 1945702 contain the supplementary crystallographic data for this paper. These data are provided free of charge by The Cambridge Crystallographic Data Centre.

## Conflict of interest

The authors declare no conflict of interest.

## Supporting information

As a service to our authors and readers, this journal provides supporting information supplied by the authors. Such materials are peer reviewed and may be re‐organized for online delivery, but are not copy‐edited or typeset. Technical support issues arising from supporting information (other than missing files) should be addressed to the authors.

Supplementary
