# Supplementary material for: Potential Precursors for Terminal Methylidene Rare‐Earth‐Metal Complexes Supported by a Superbulky Tris(pyrazolyl)borato Ligand
Source: Chemistry. 2019 Oct 22;25(64):14711–20. doi: 10.1002/chem.201903606 (PMC7687121; doi:10.1002/chem.201903606)
Supplement: Supplementary file 1 — Supplementary [file CHEM-25-14711-s001.pdf]

CHEMISTRY
A European Journal

Supporting Information

# Potential Precursors for Terminal Methylidene Rare-Earth-Metal Complexes Supported by a Superbulky Tris(pyrazolyl)borato Ligand

Verena M. Birkelbach, Renita Thim, Christoph Stuhl, Cäcilia Maichle-Mössmer, and Reiner Anwander*[a]

chem_201903606_sm_miscellaneous_information.pdf

# Supporting Information

# Table of contents

## NMR spectroscopy


| | |
|---|---|
| $^{1}$H NMR, $^{13}$C{$^{1}$H} NMR, $^{11}$B{$^{1}$H} NMR, $^{19}$F{$^{1}$H} NMR spectra of complex **1-Y** | Pages S2-S4 |
| $^{1}$H NMR, $^{13}$C{$^{1}$H} NMR, $^{11}$B{$^{1}$H} NMR, $^{19}$F{$^{1}$H} NMR spectra of complex **1-Lu** | Pages S4-S6 |
| $^{1}$H NMR, $^{13}$C{$^{1}$H} NMR, $^{11}$B{$^{1}$H} NMR, $^{19}$F{$^{1}$H} NMR spectra of complex **2-Lu** | Pages S6-S8 |
| $^{1}$H NMR, $^{13}$C{$^{1}$H} NMR, $^{11}$B{$^{1}$H} NMR, $^{19}$F{$^{1}$H} NMR spectra of complex **3-Lu** | Pages S9-S10 |
| $^{1}$H NMR, $^{13}$C{$^{1}$H} NMR, $^{11}$B{$^{1}$H} NMR, $^{19}$F{$^{1}$H} NMR spectra of complex **4-Lu** | Pages S10-S12 |
| $^{1}$H NMR, $^{13}$C{$^{1}$H} NMR, $^{11}$B{$^{1}$H} NMR spectra of complex **5-Y** | Pages S12-S13 |
| $^{1}$H NMR, $^{13}$C{$^{1}$H} NMR, $^{11}$B{$^{1}$H} NMR spectra of complex **5-Lu** | Pages S14-S15 |
| $^{1}$H NMR, $^{13}$C{$^{1}$H} NMR, $^{11}$B{$^{1}$H} NMR spectra of complex **6-Y** | Pages S15-S16 |
| $^{1}$H NMR, $^{13}$C{$^{1}$H} NMR, $^{11}$B{$^{1}$H} NMR spectra of complex **6-Lu** | Pages S17-S18 |
| $^{1}$H NMR, $^{13}$C{$^{1}$H} NMR, $^{11}$B{$^{1}$H} NMR spectra of complex **7-Lu** | Pages S19-S20 |
| $^{1}$H NMR, $^{13}$C{$^{1}$H} NMR, $^{11}$B{$^{1}$H} NMR, $^{29}$Si{$^{1}$H} NMR spectra of complex **8-Lu** | Pages S20-S21 |
| $^{1}$H NMR, $^{13}$C{$^{1}$H} NMR, $^{11}$B{$^{1}$H} NMR spectra of complex **9-Lu** | Pages S22-S23 |


## X-ray structure analyses


| | |
|---|---|
| Comprehensive crystallographic data for compounds **1-Lu**, **2-Lu**, **4-Lu**, **5-Lu**, **6-Lu**, **7-Lu**, **8-Lu**, and **9-Lu** | Pages S24-25 |


## Cone-angle analyses


| | |
|---|---|
| Cone angle calculations | Pages S26-28 |
| Raw data files for cone angle calculation | Pages S29-40 |

## NMR spectroscopy

The solvent residual peaks are marked with an asterisk (*).

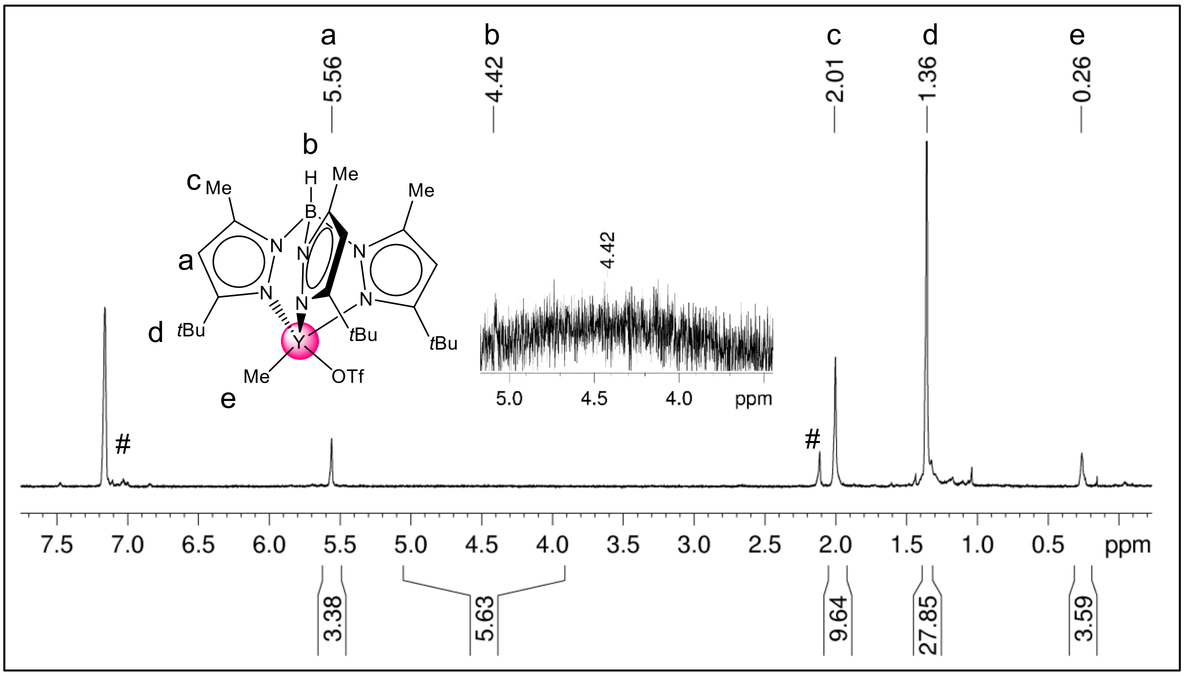


**Figure S1.** $^{1}$H NMR spectrum (250 MHz, [D$_6$]benzene of complex [Tp$^{tBu,Me}$YMe(OTf)] (**1-Y**) at 26 °C with traces of toluene (#).

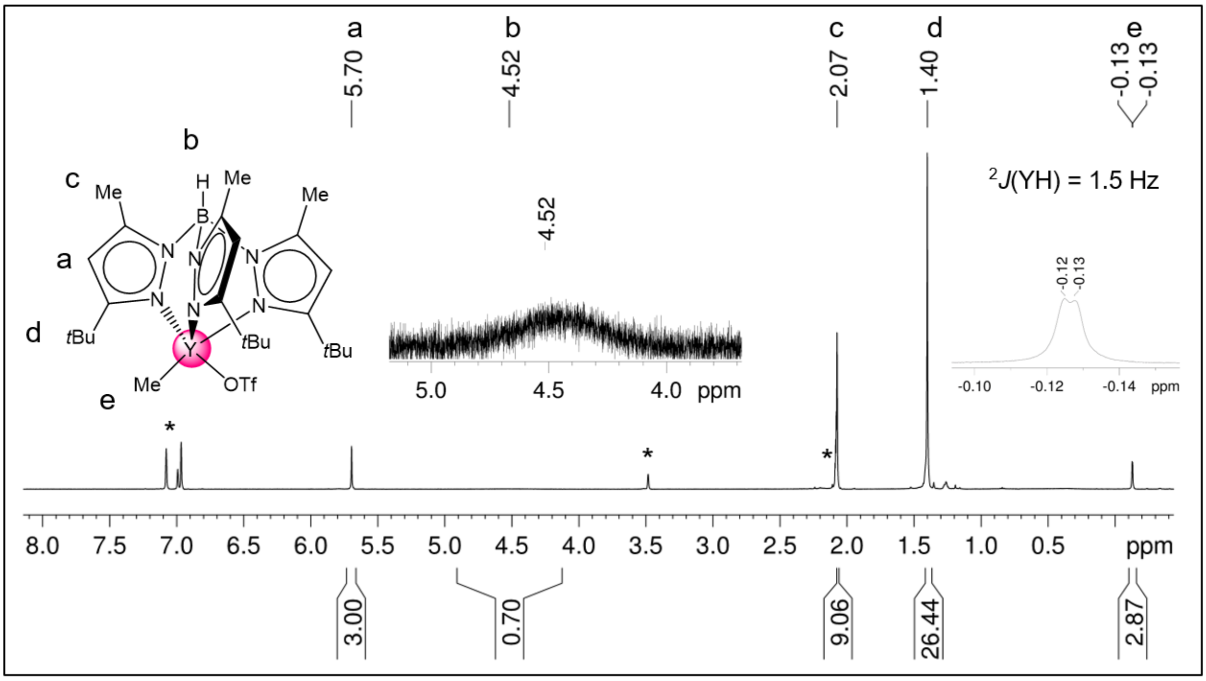


**Figure S2.** $^{1}$H NMR spectrum (500 MHz, [D$_8$]toluene and a few drops of [D$_8$]thf due to solubility issues at lower temperatures) of complex [Tp$^{tBu,Me}$YMe(OTf)] (**1-Y**) at 0 °C.

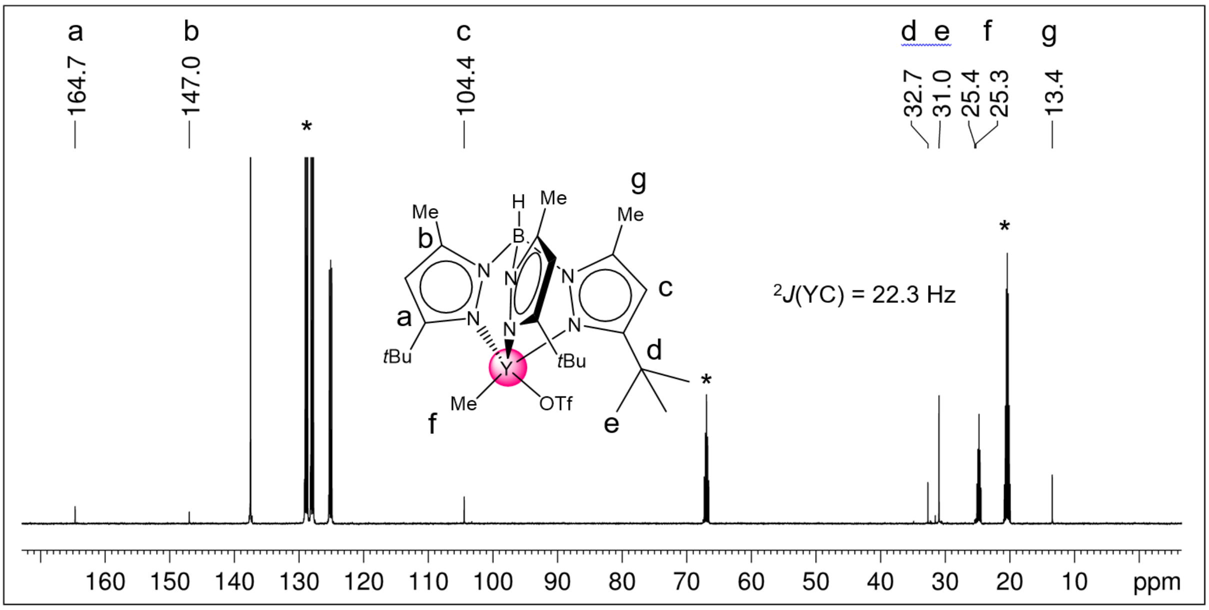


**Figure S3.** $^{13}$C{$^1$H} NMR spectrum (126 MHz, [D8]toluene and a few drops of [D8]thf due to solubility issues at lower temperatures) of complex [Tp$^{tBu,Me}$YMe(OTf)] (**1-Y**) at 0 °C. $^{13}$C NMR resonances for the triflate groups could not be detected.

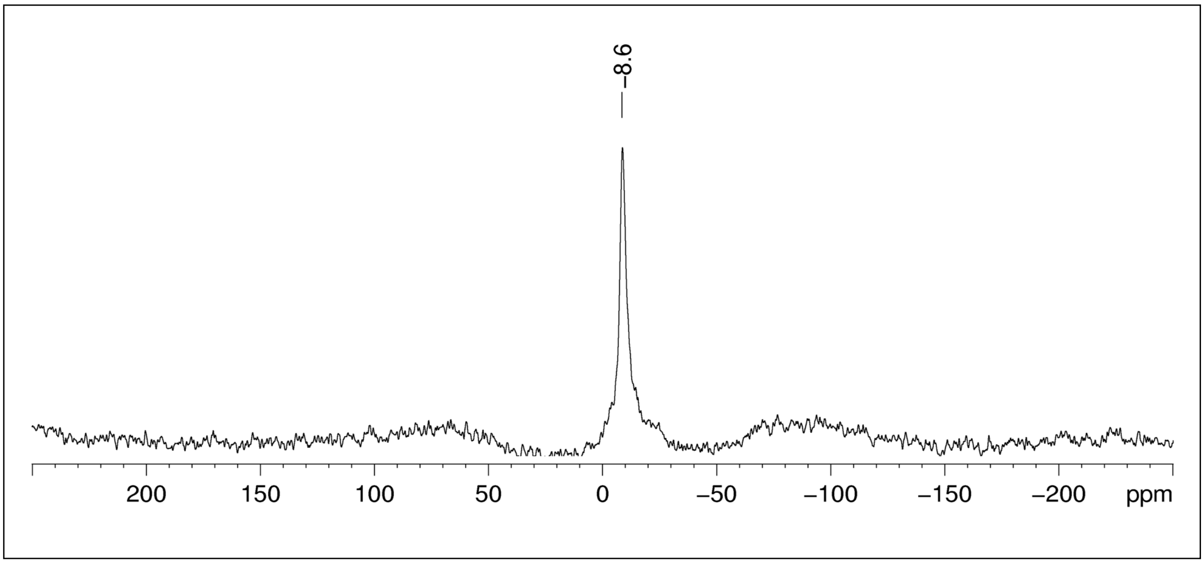


**Figure S4.** $^{11}$B{$^1$H} NMR spectrum (80 MHz, [D6]benzene) of complex [Tp$^{tBu,Me}$YMe(OTf)] (**1-Y**) at 26 °C.

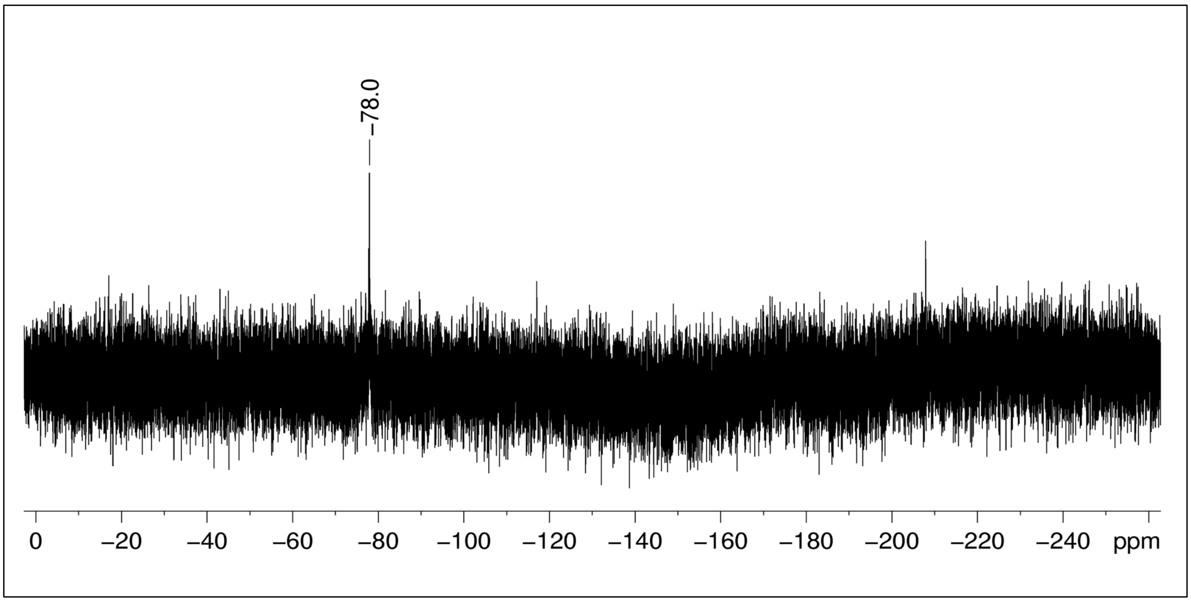


**Figure S5.** $^{19}F\{^{1}H\}$ NMR spectrum (376 MHz, [D6]benzene) of complex [Tp$^{tBu,Me}$YMe(OTf)] (**1-Y**) at 26 °C.

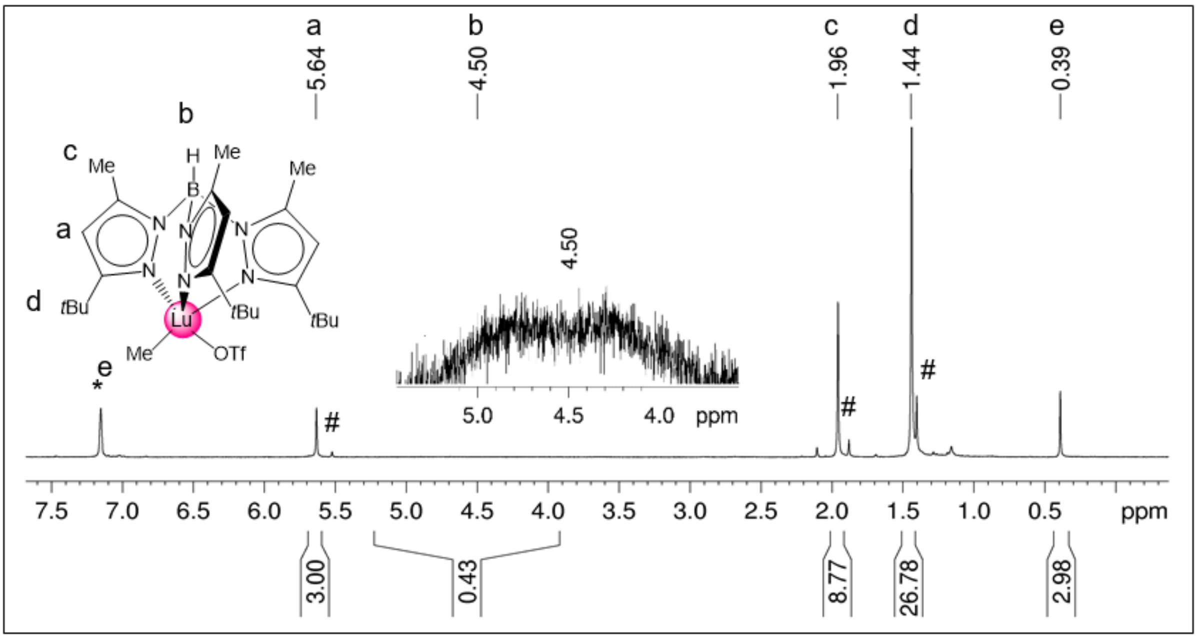


**Figure S6.** $^{1}H$ NMR spectrum (250 MHz, [D6]benzene) of complex [Tp$^{tBu,Me}$LuMe(OTf)] (**1-Lu**) at 26 °C with minor impurities of the reactant [Tp$^{tBu,Me}$Lu(OTf)$_2$] (#).

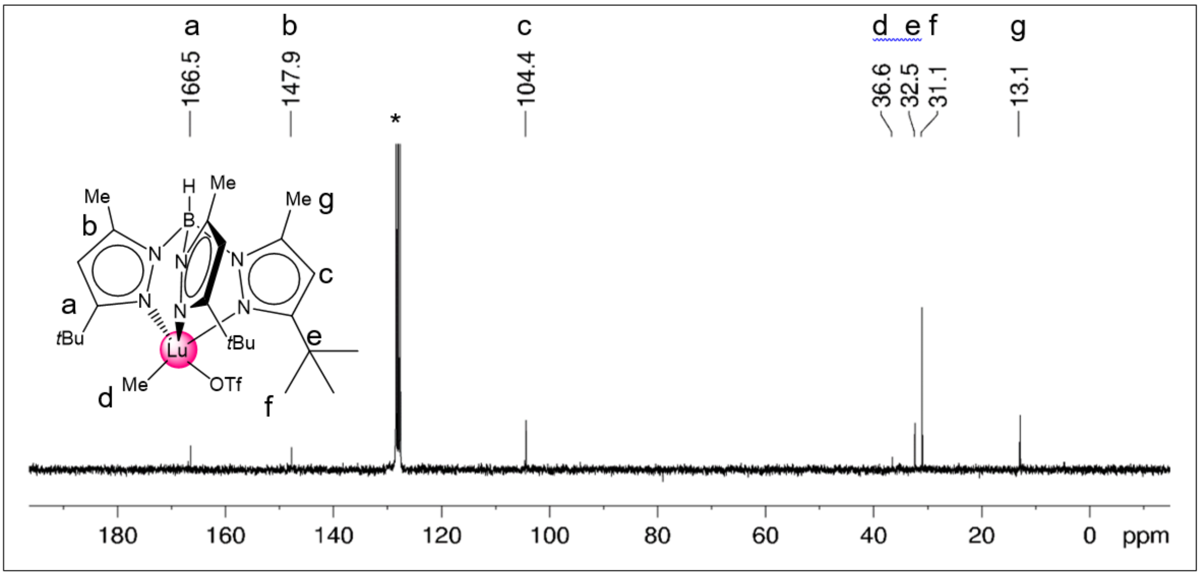


**Figure S7.** $^{13}C\{^{1}H\}$ NMR spectrum (63 MHz, [D6]benzene) of complex [Tp$^{tBu,Me}$LuMe(OTf)] (**1-Lu**) at 26 °C. $^{13}C$ NMR resonances for the triflate groups could not be detected.

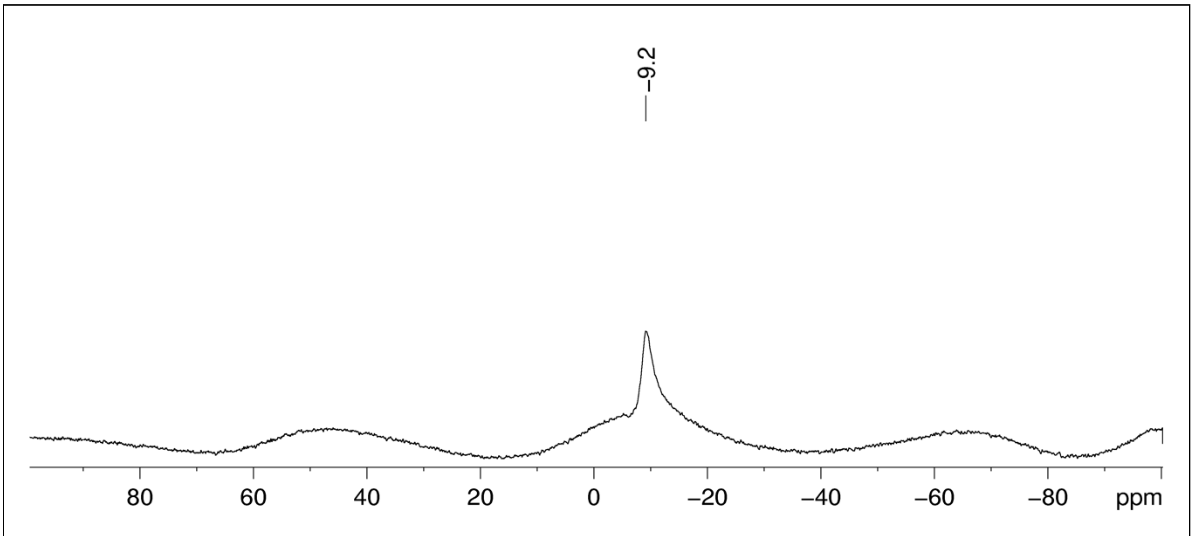


**Figure S8.** $^{11}B\{^{1}H\}$ NMR spectrum (80 MHz, [D6]benzene) of complex [Tp$^{tBu,Me}$LuMe(OTf)] (**1-Lu**) at 26 °C.

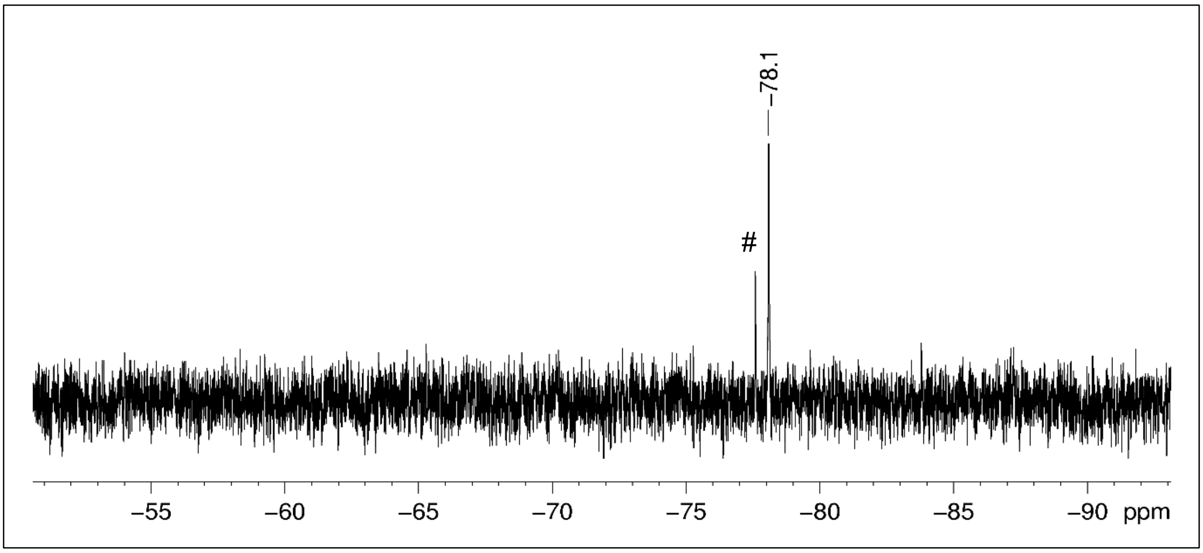


**Figure S9.** $^{19}F\{^{1}H\}$ NMR spectrum (376 MHz, [D6]benzene) of complex [Tp$^{tBu,Me}$LuMe(OTf)] (**1-Lu**) at 26 °C with minor impurities of [Tp$^{tBu,Me}$Lu(OTf)$_2$] (#).

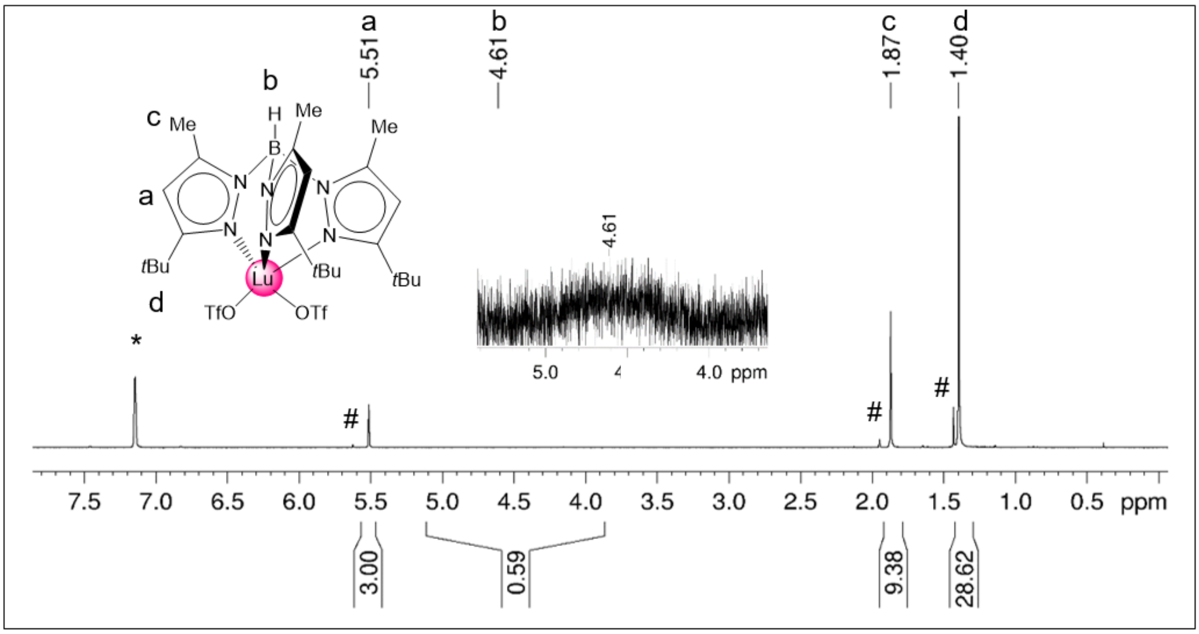


**Figure S10.** $^{1}H$ NMR spectrum (250 MHz, [D6]benzene) of complex [Tp$^{tBu,Me}$Lu(OTf)$_2$] (**2-Lu**) at 26 °C with minor impurities of [Tp$^{tBu,Me}$LuMe(OTf)] (#).

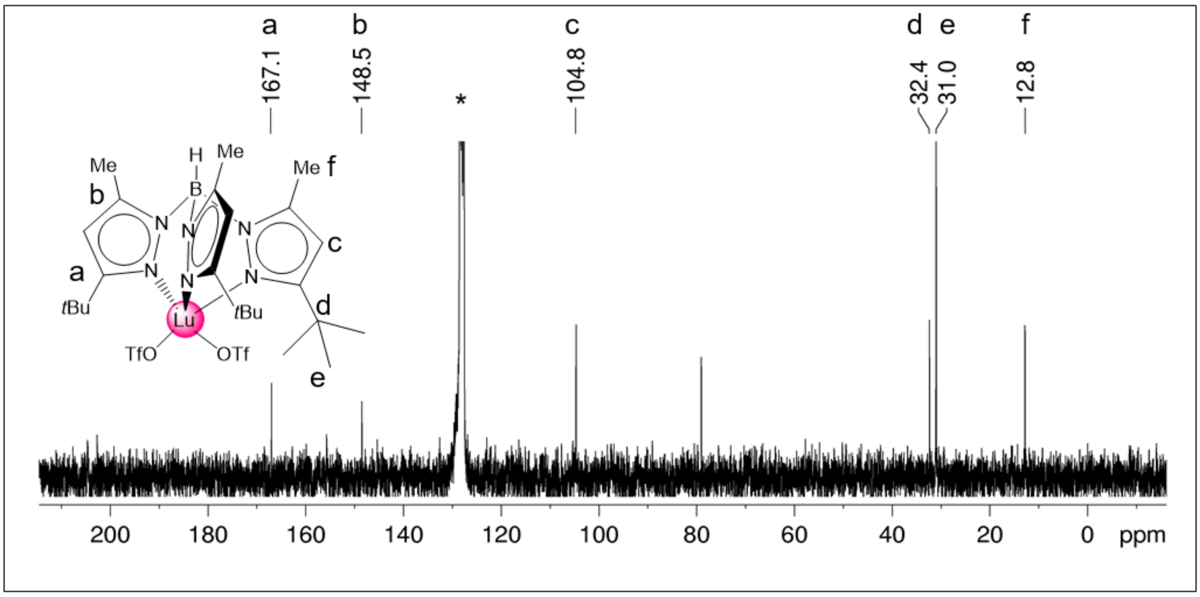


**Figure S11.** $^{13}C\{^1H\}$ NMR spectrum (63 MHz, [D$_6$]benzene) of complex [Tp$^{tBu,Me}$Lu(OTf)$_2$] (**2-Lu**) at 26 °C. $^{13}$C NMR resonances for the triflate groups could not be detected.

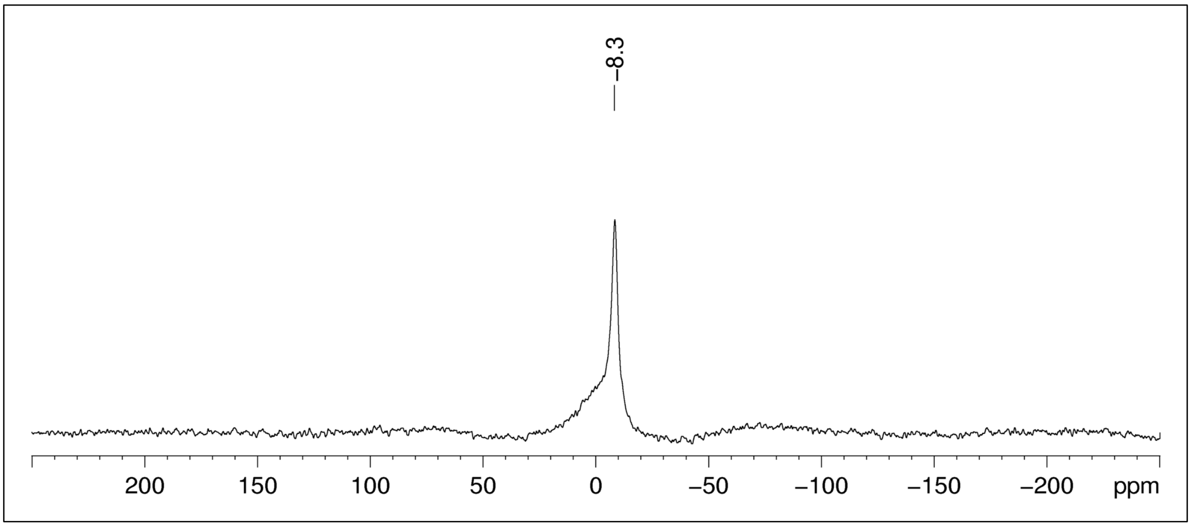


**Figure S12.** $^{11}B\{^1H\}$ NMR spectrum (80 MHz, [D$_6$]benzene) of complex [Tp$^{tBu,Me}$Lu(OTf)$_2$] (**2-Lu**) at 26 °C.

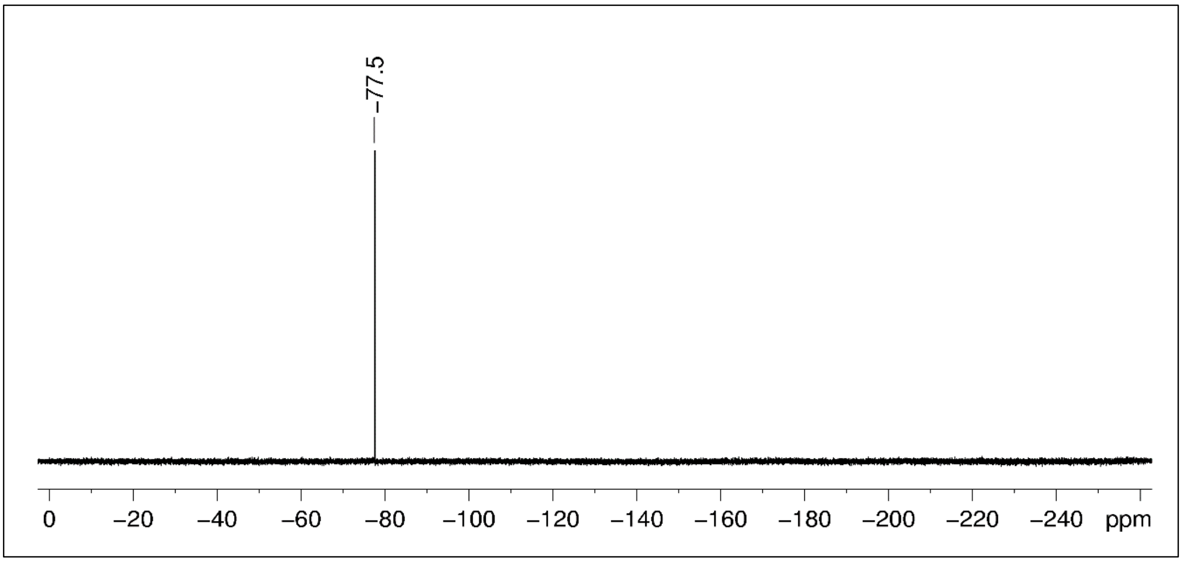


**Figure S13.** $^{19}F\{^{1}H\}$ NMR spectrum (376 MHz, [D6]benzene) of complex [Tp$^{tBu,Me}$Lu(OTf)$_2$] (**2-Lu**) at 26 °C.

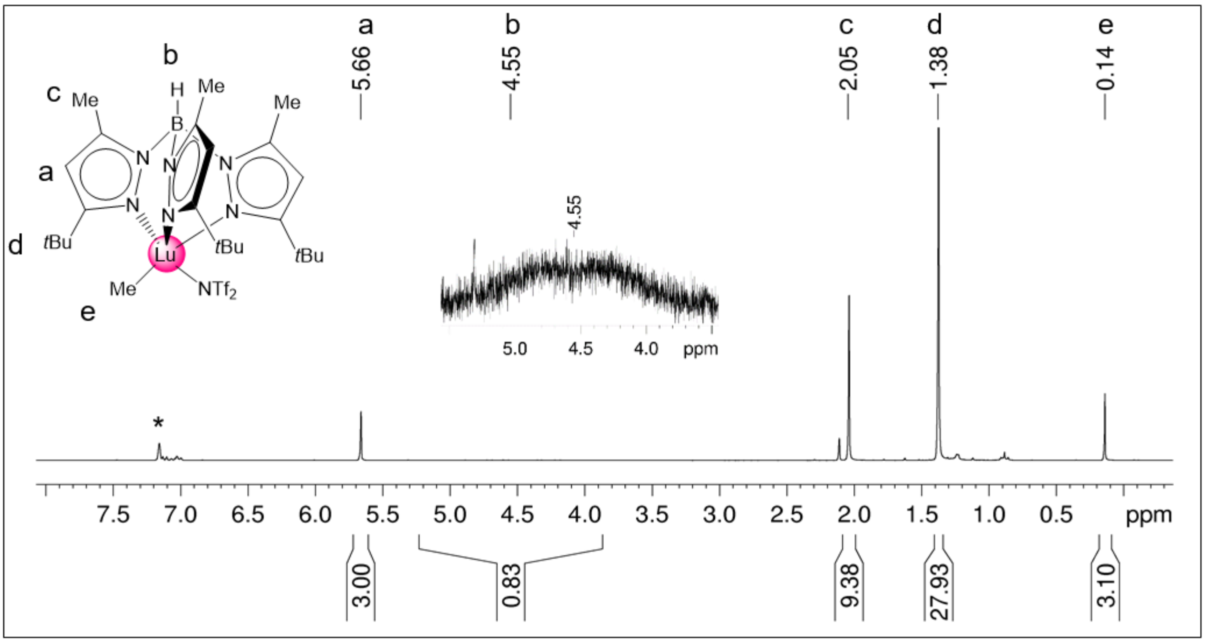


**Figure S14.** $^{1}H$ NMR spectrum (250 MHz, [D6]benzene) of complex [Tp$^{tBu,Me}$LuMe(NTf$_2$)] (**3-Lu**) at 26 °C.

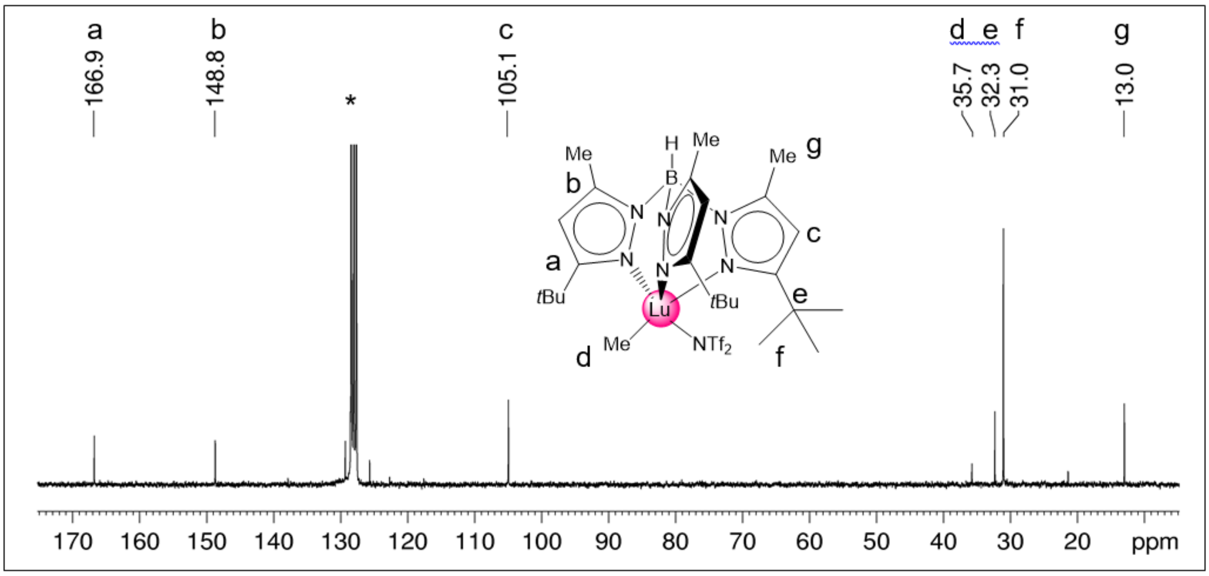


**Figure S15.** $^{13}C\{^1H\}$ NMR spectrum (63 MHz, [D$_6$]benzene) of complex [Tp$^{tBu,Me}$LuMe(NTf$_2$)] (**3-Lu**) at 26 °C. $^{13}$C NMR resonances for the triflate groups could not be detected.

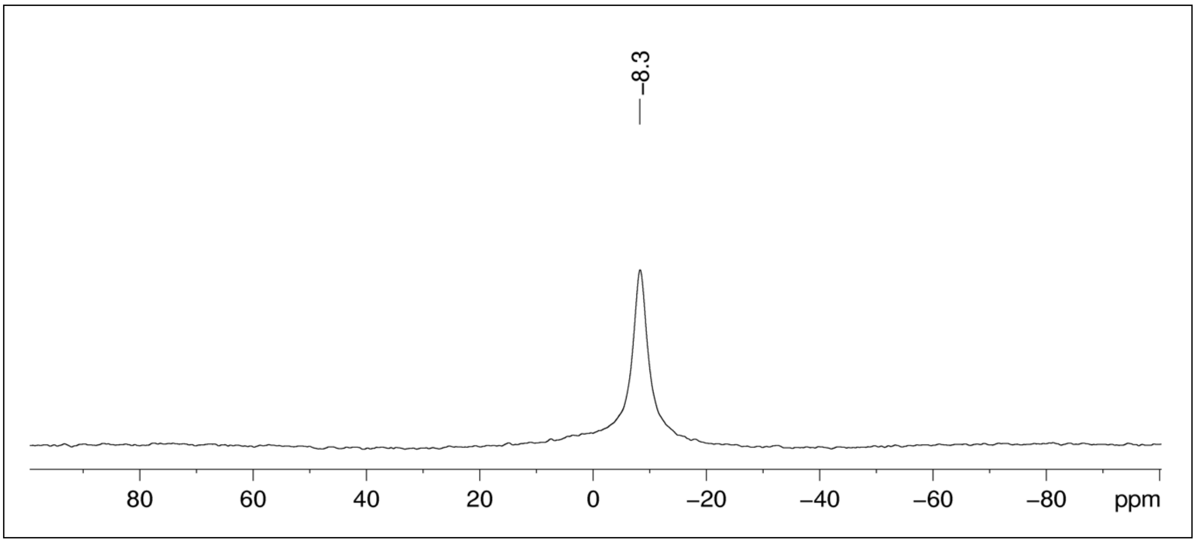


**Figure S16.** $^{11}B\{^1H\}$ NMR spectrum (80 MHz, [D$_6$]benzene) of complex [Tp$^{tBu,Me}$LuMe(NTf$_2$)] (**3-Lu**) at 26 °C.

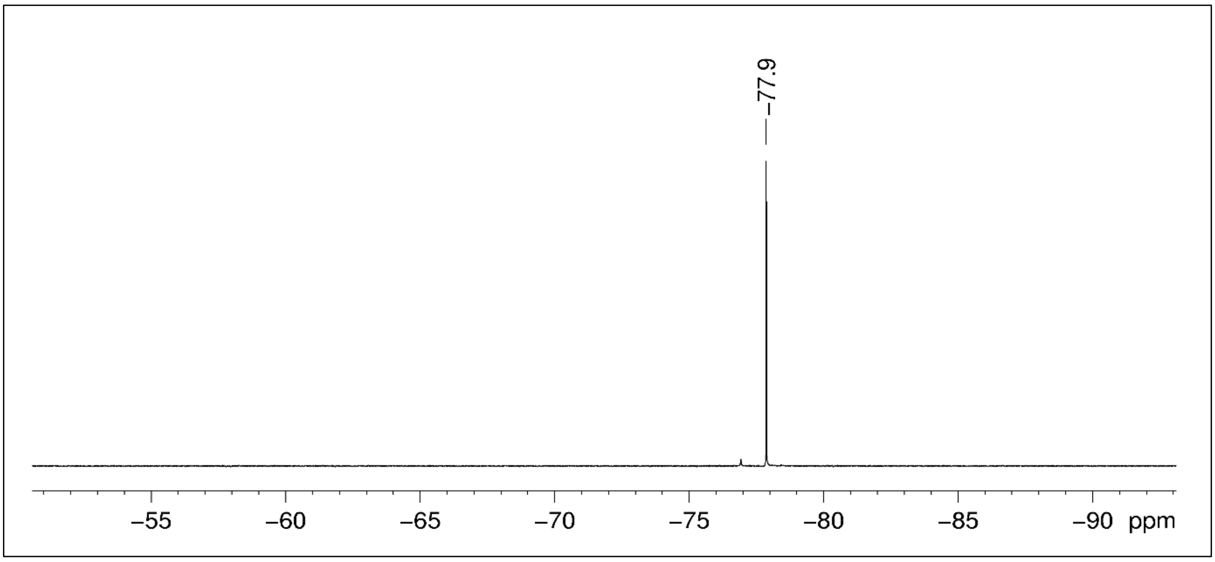


**Figure S17.** $^{19}F\{^{1}H\}$ NMR spectrum (376 MHz, [D6]benzene) of complex [Tp$^{tBu,Me}$LuMe(NTf2)] (**3-Lu**) at 26 °C.

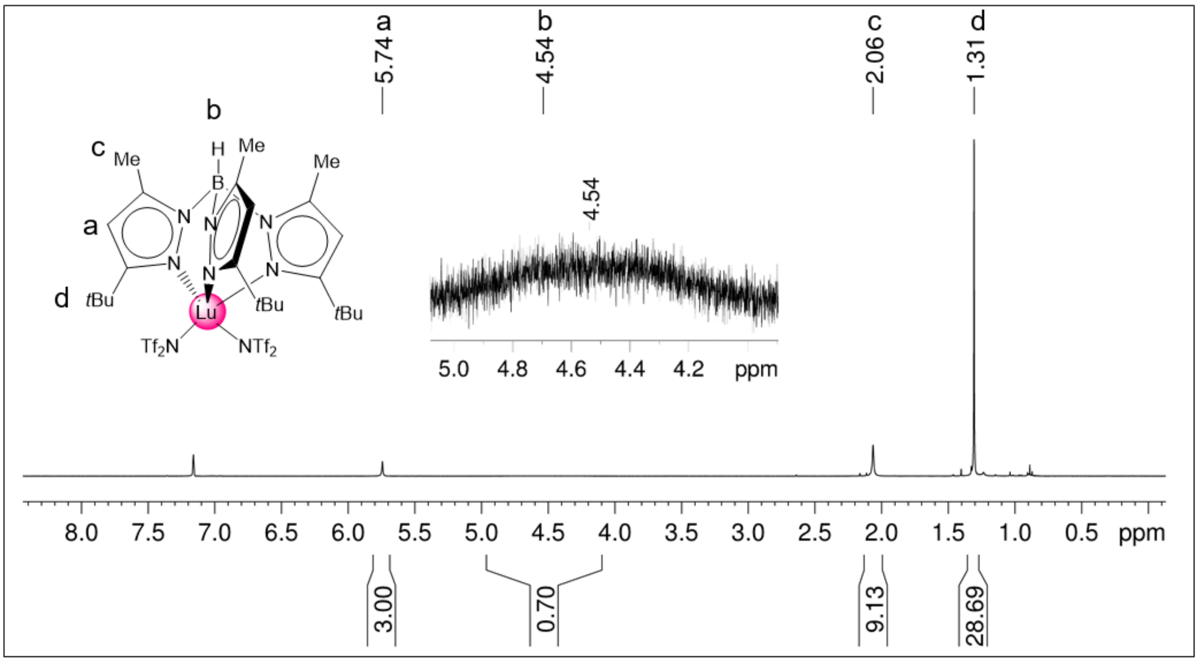


**Figure S18.** $^{1}H$ NMR spectrum (250 MHz, [D6]benzene) of complex [Tp$^{tBu,Me}$Lu(NTf2)2] (**4-Lu**) at 26 °C.

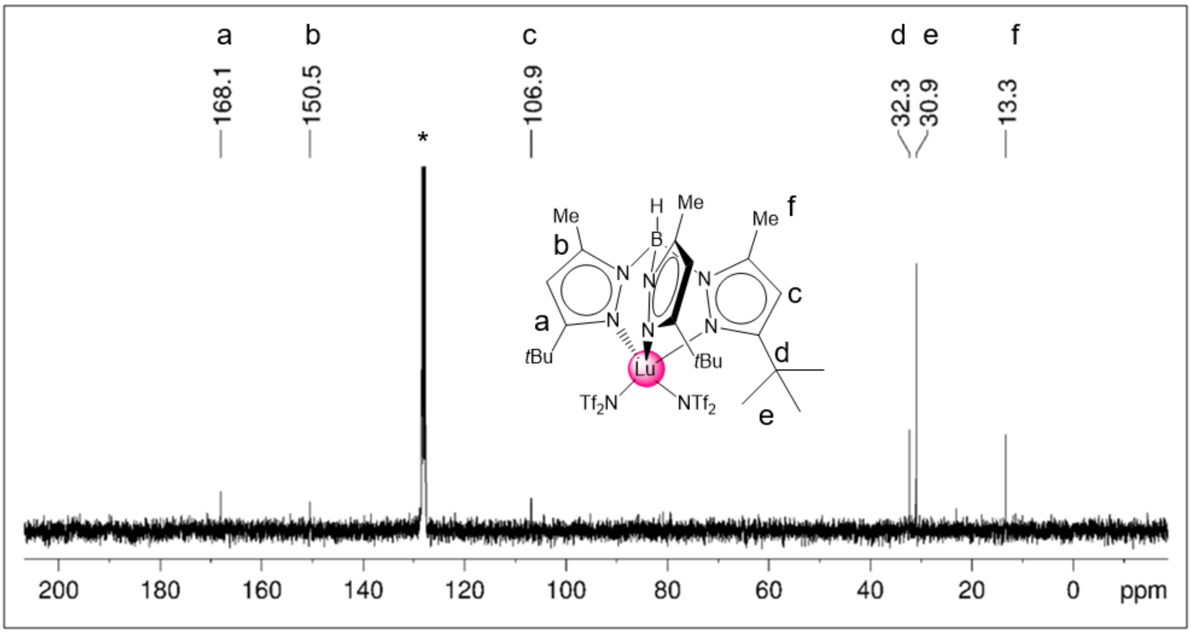


**Figure S19.** $^{13}C\{^{1}H\}$ NMR spectrum (63 MHz, [D6]benzene) of complex [Tp$^{tBu,Me}$Lu(NTf$_2$)$_2$] (**4-Lu**) at 26 °C. $^{13}$C NMR resonances for the triflate groups could not be detected.

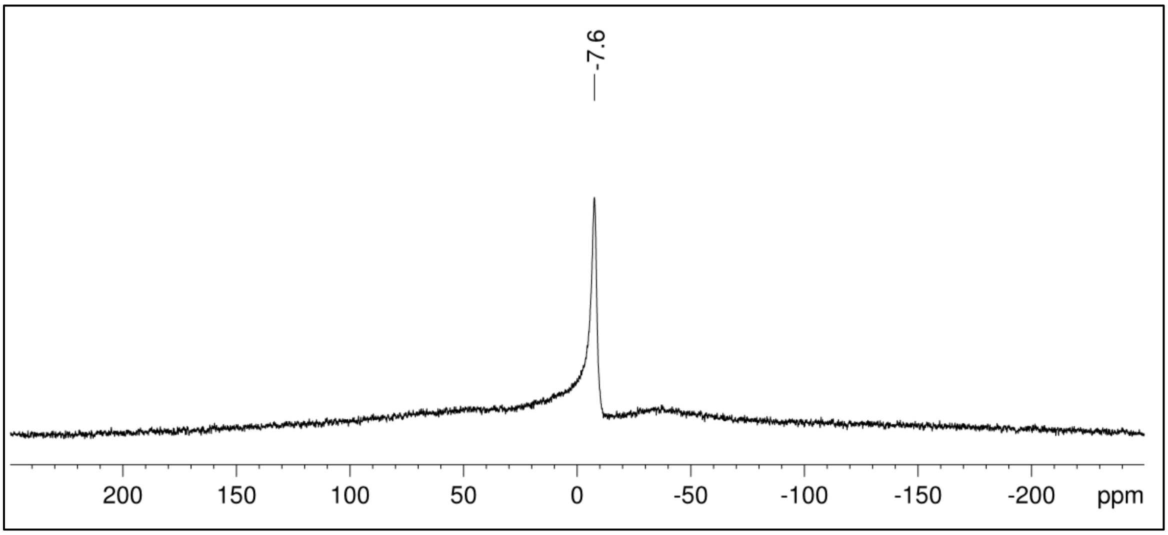


**Figure S20.** $^{11}B\{^{1}H\}$ NMR spectrum (80 MHz, [D6]benzene) of complex [Tp$^{tBu,Me}$Lu(NTf$_2$)$_2$] (**4-Lu**) at 26 °C.

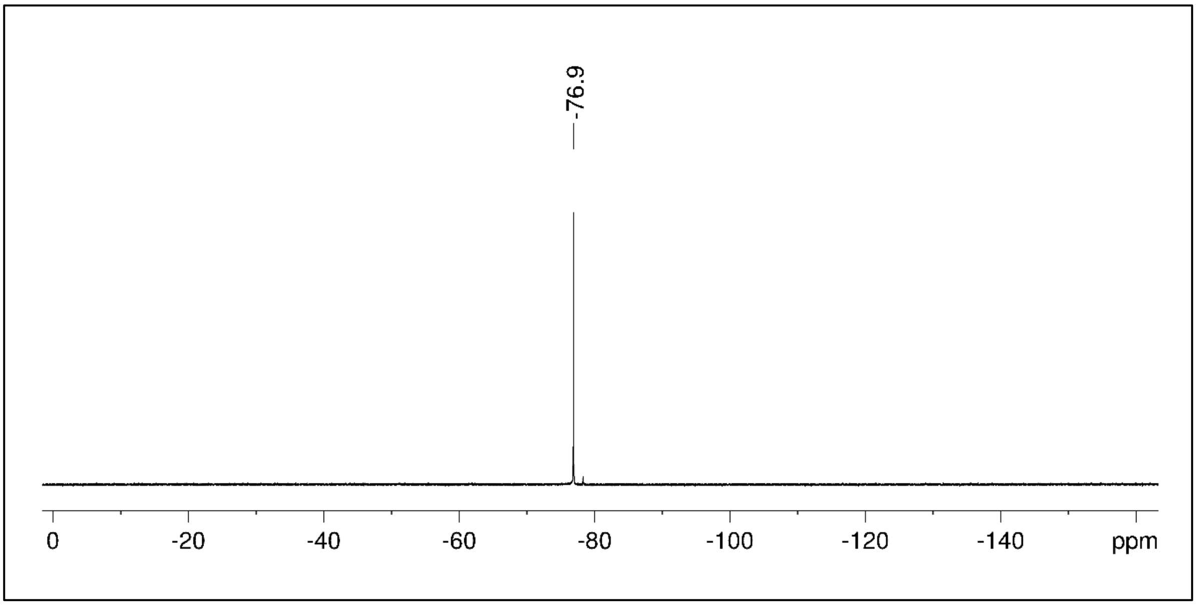


**Figure S21.** $^{19}F\{^{1}H\}$ NMR spectrum (376 MHz, [D$_6$]benzene) of complex [Tp$^{tBu,Me}$Lu(NTf$_2$)$_2$] (**4-Lu**) at 26 °C.

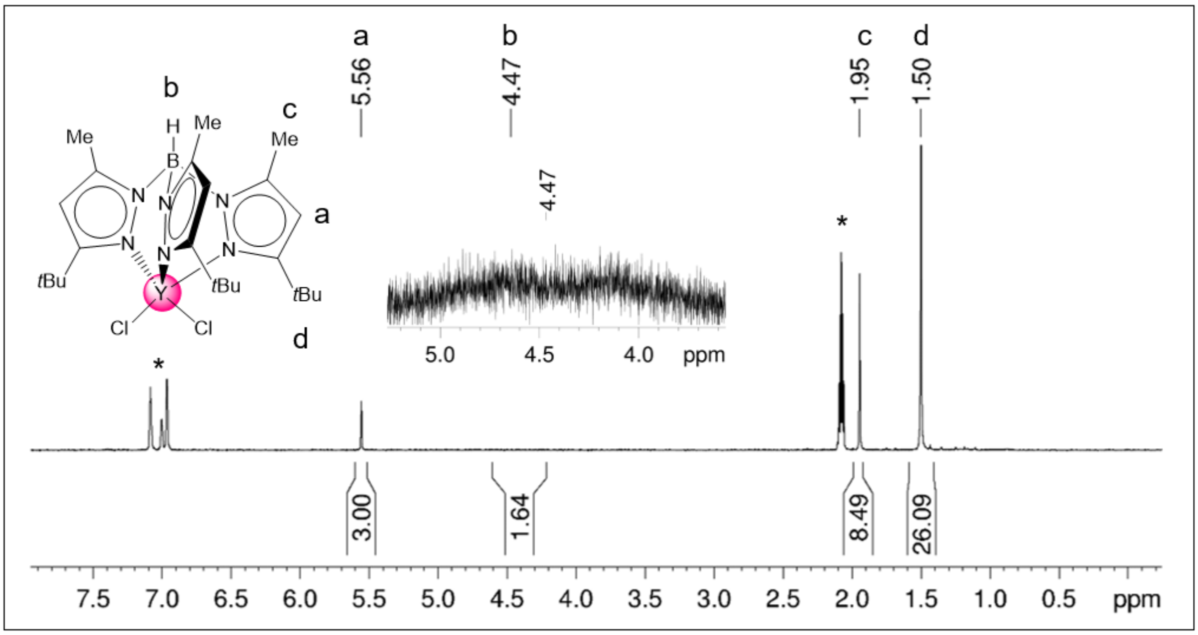


**Figure S22.** $^{1}H$ NMR spectrum (250 MHz, [D$_8$]toluene) of complex [Tp$^{tBu,Me}$YCl$_2$] (**5-Y**) at 26 °C.

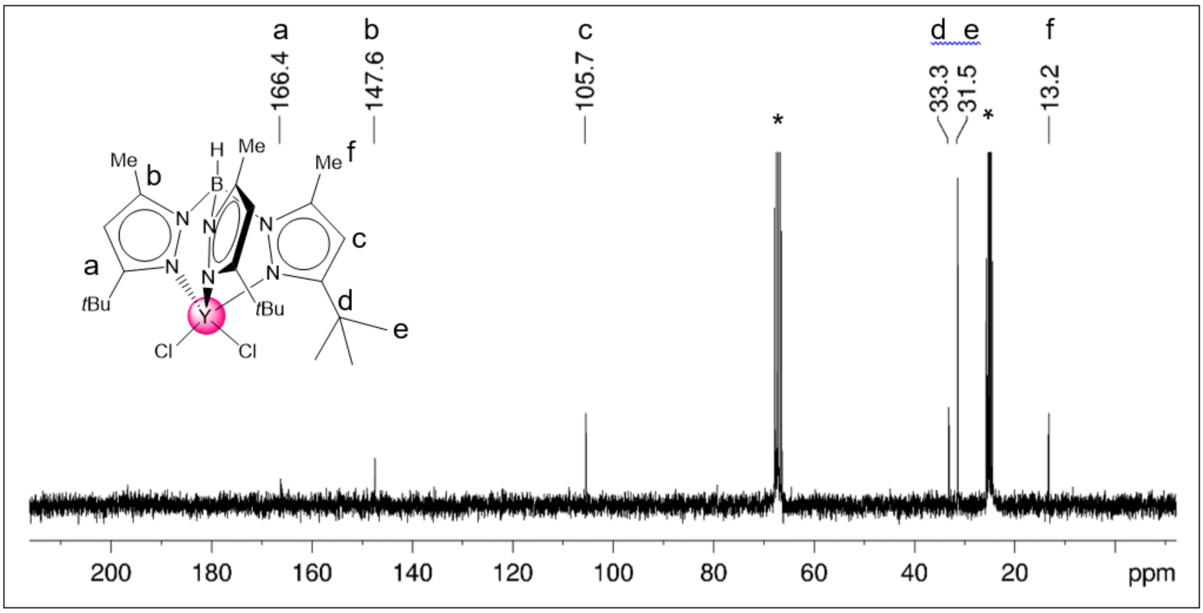


**Figure S23.** $^{13}C\{^1H\}$ NMR spectrum (63 MHz, [D$_8$]thf) of complex [Tp$^{tBu,Me}$YCl$_2$] (**5-Y**) at 26 °C.

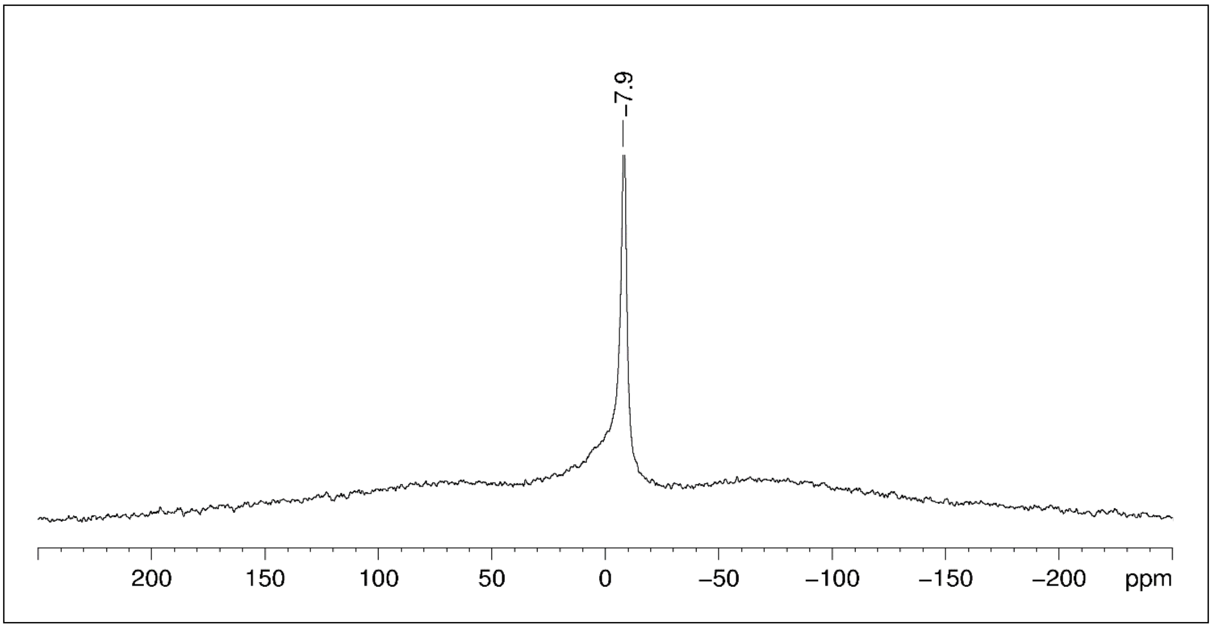


**Figure S24.** $^{11}B\{^1H\}$ NMR spectrum (80 MHz, [D$_8$]thf) of complex [Tp$^{tBu,Me}$YCl$_2$] (**5-Y**) at 26 °C.

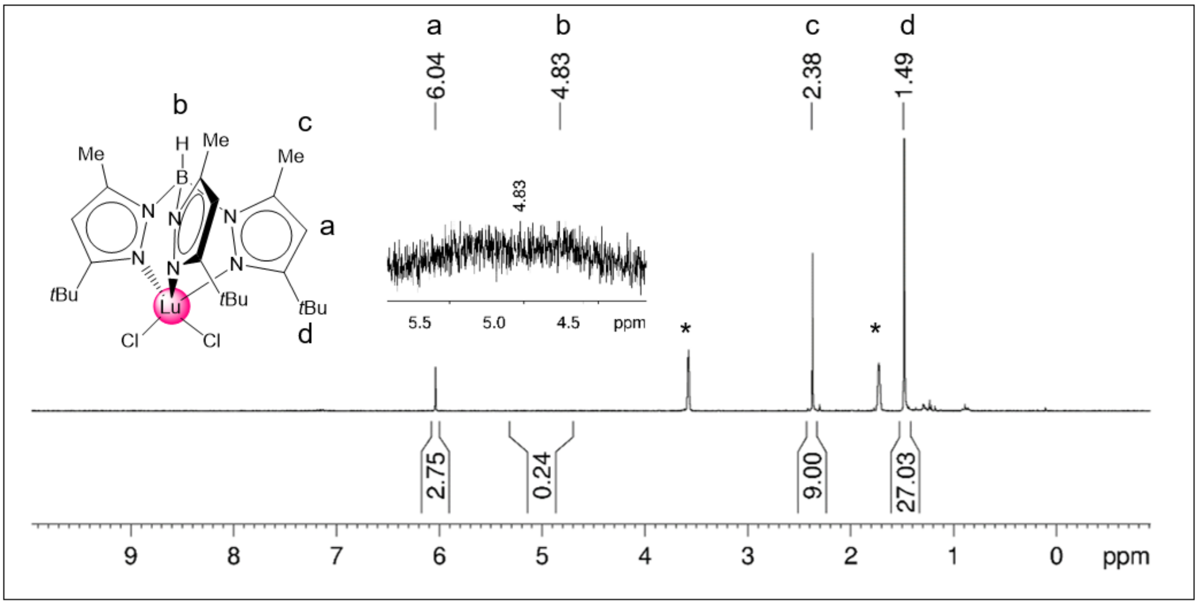


**Figure S25.** $^{1}$H NMR spectrum (400 MHz, [D$_8$]thf) of complex [Tp$^{tBu,Me}$YLuCl$_2$] (**5-Lu**) at 26 °C.

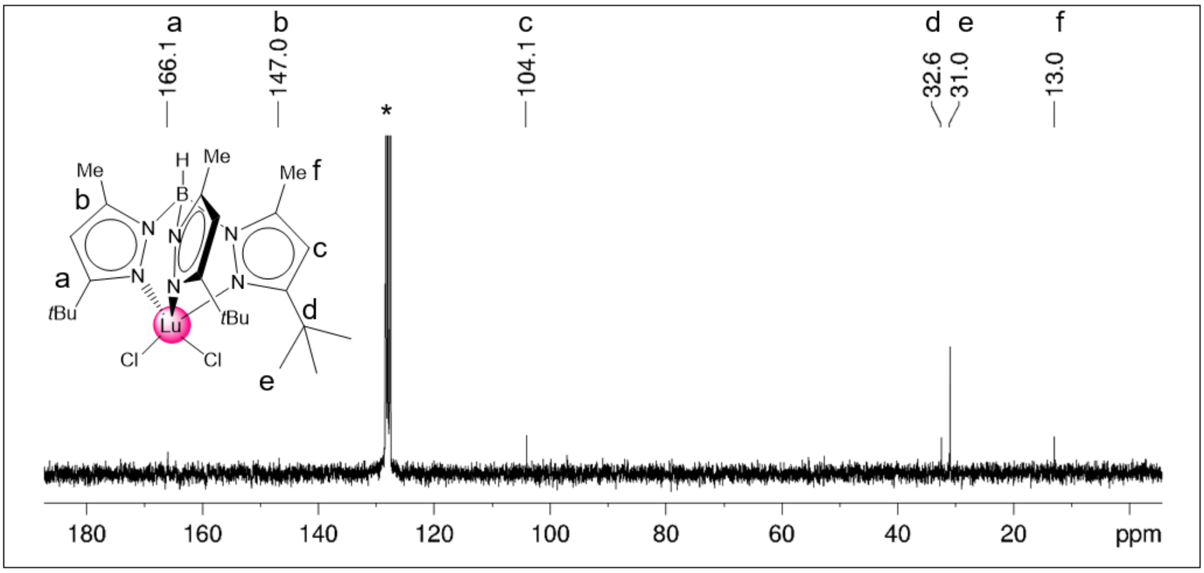


**Figure S26.** $^{13}$C{$^{1}$H} NMR spectrum (63 MHz, [D$_6$]benzene) of complex [Tp$^{tBu,Me}$YLuCl$_2$] (**5-Lu**) at 26 °C.

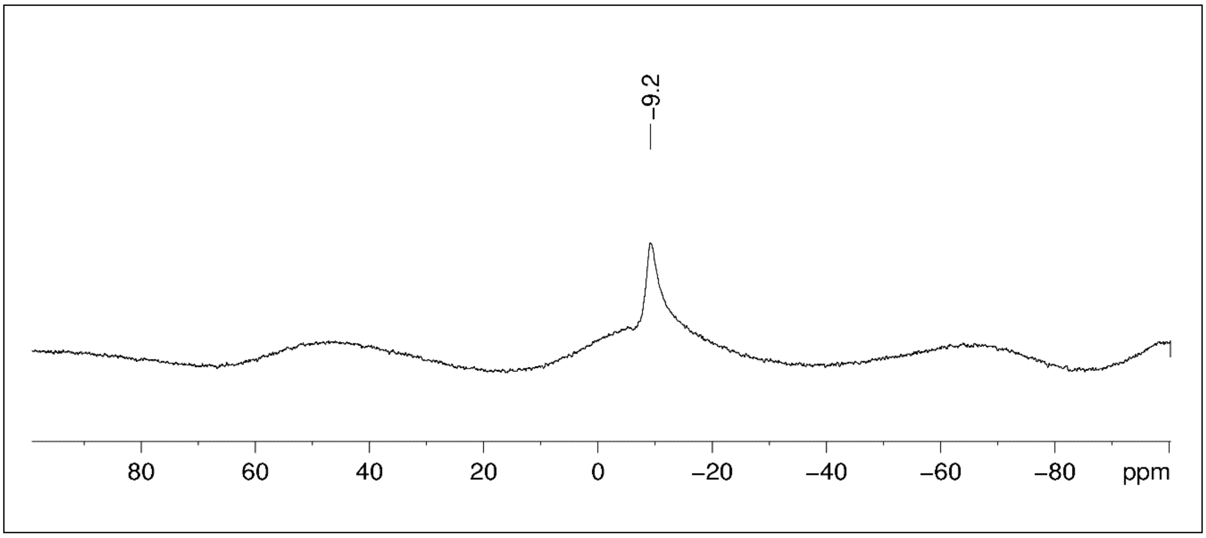


**Figure S27.** $^{11}B\{^{1}H\}$ NMR spectrum (80 MHz, [D8]thf) of complex [Tp$^{tBu,Me}$YLuCl$_2$] (**5-Lu**) at 26 °C.

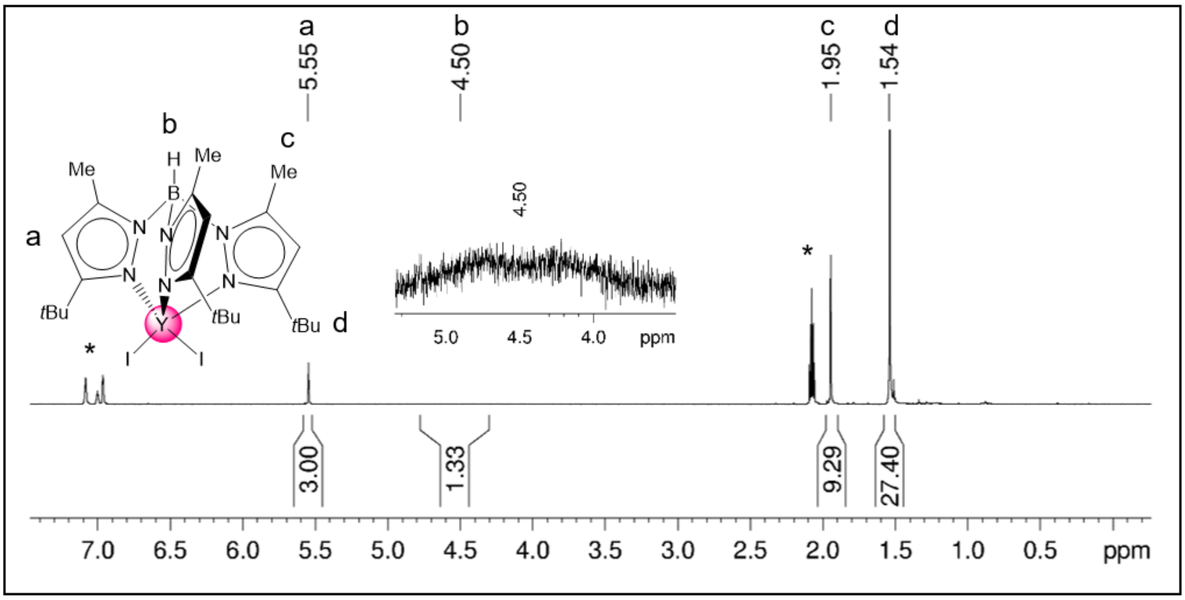


**Figure S28.** $^{1}H$ NMR spectrum (250 MHz, [D8]toluene) of complex [Tp$^{tBu,Me}$YI$_2$] (**6-Y**) at 26 °C.

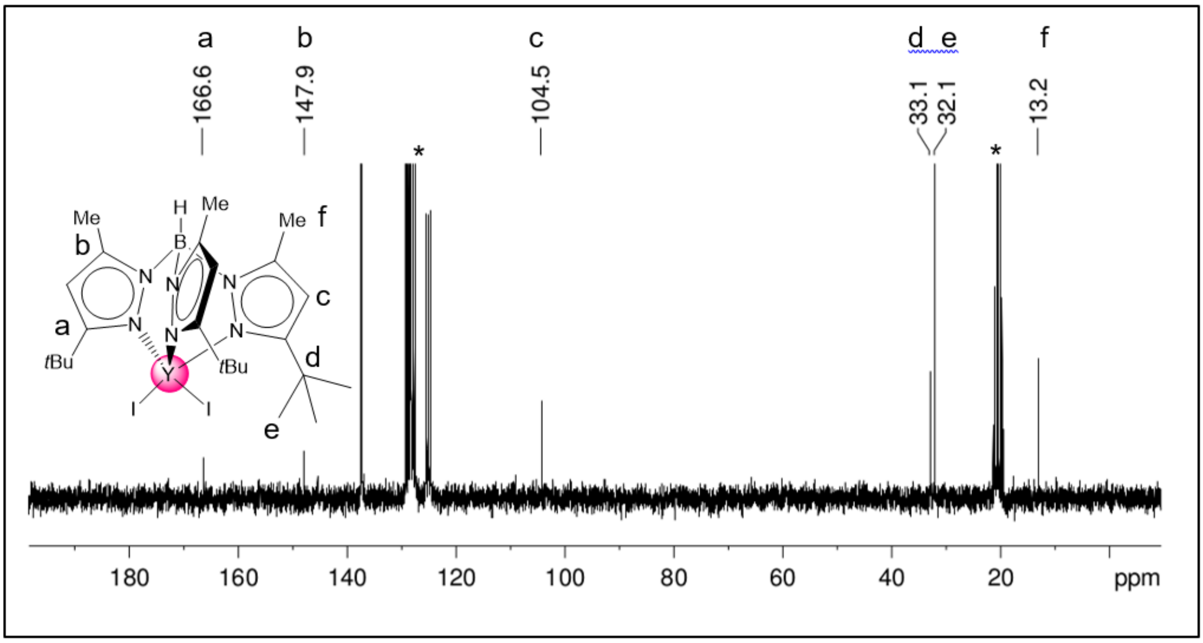


**Figure S29.** $^{13}C\{^{1}H\}$ NMR spectrum (63 MHz, [D8]toluene) of complex [Tp$^{tBu,Me}$YI$_2$] (**6-Y**) at 26 °C.

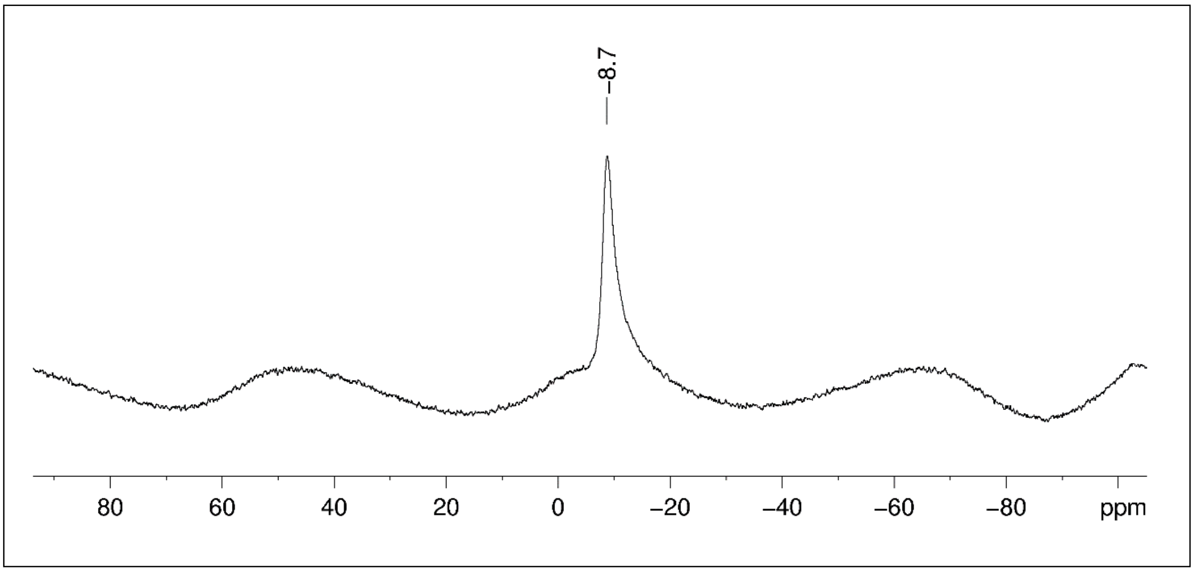


**Figure S30.** $^{11}B\{^{1}H\}$ NMR spectrum (80 MHz, [D8]toluene) of complex [Tp$^{tBu,Me}$YI$_2$] (**6-Y**) at 26 °C.

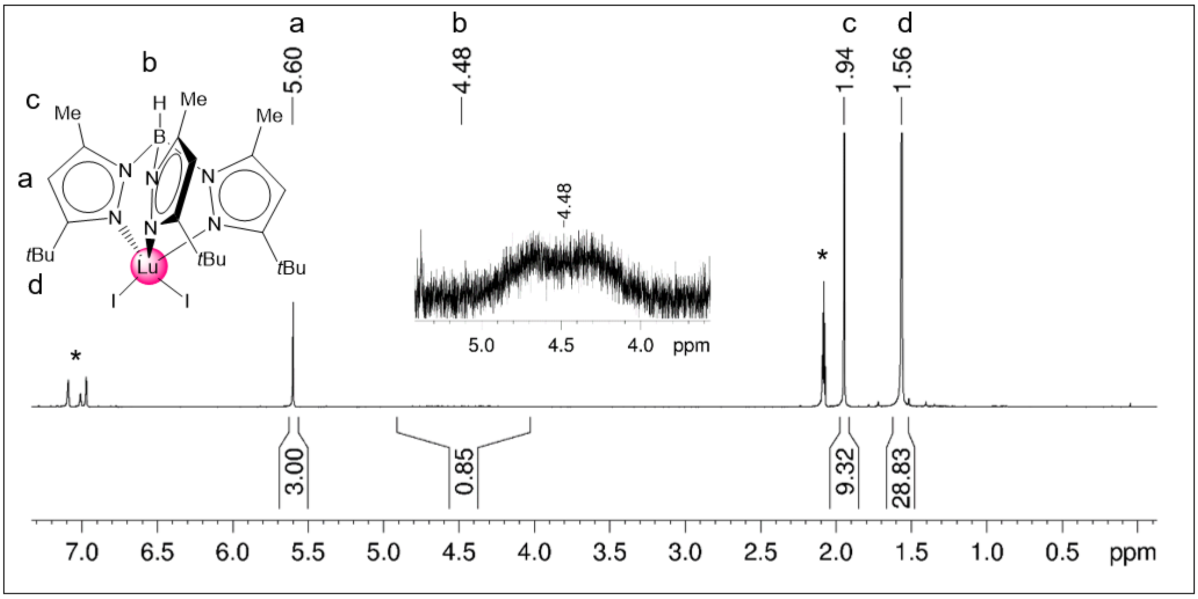


**Figure S31.** $^{1}H$ NMR spectrum (250 MHz, [D8]toluene) of complex [$Tp^{tBu,Me}LuI_2$] (**6-Lu**) at 26 °C.

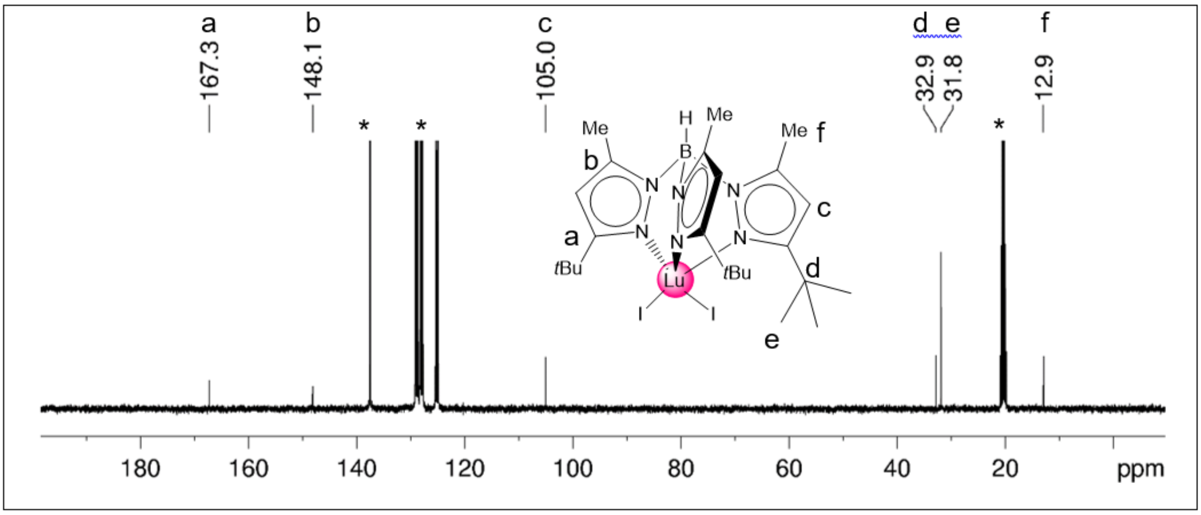


**Figure S32.** $^{13}C\{^{1}H\}$ NMR spectrum (101 MHz, [D8]toluene) of complex [$Tp^{tBu,Me}LuI_2$] (**6-Lu**) at 26 °C.

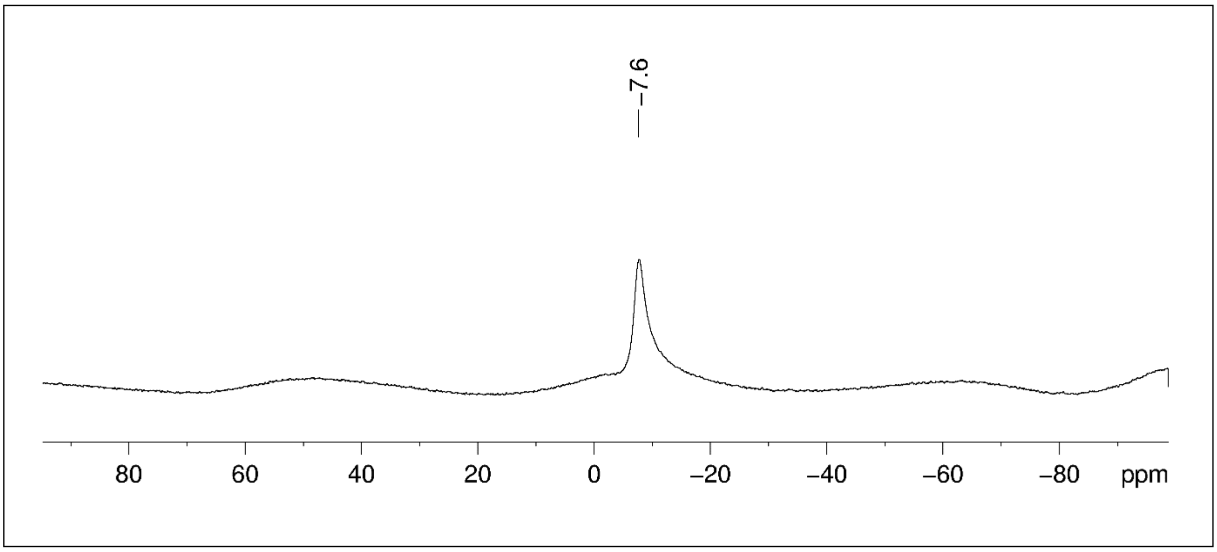


**Figure S33.** $^{11}B\{^{1}H\}$ NMR spectrum (80 MHz, [D8]toluene) of complex [Tp$^{tBu,Me}$Lu$I_2$] (**6-Lu**) at 26 °C.

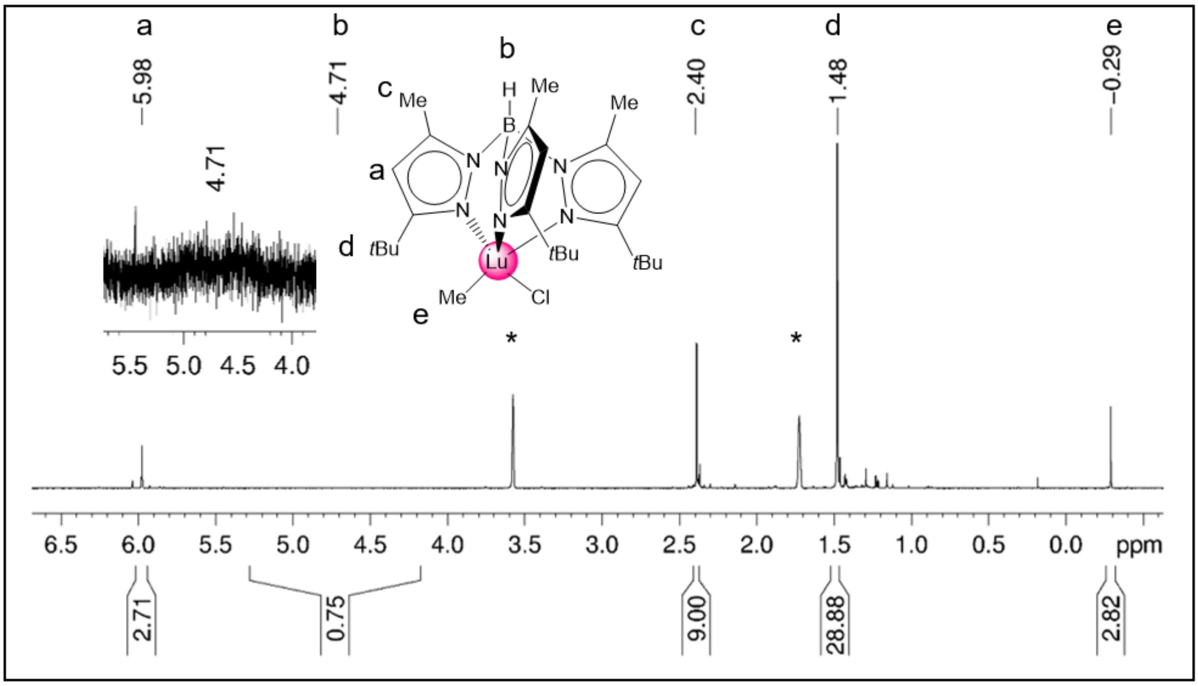


**Figure S34.** $^{1}H$ NMR spectrum (250 MHz, [D8]thf) of complex [Tp$^{tBu,Me}$LuMeCl] (**7-Lu**) at 26 °C.

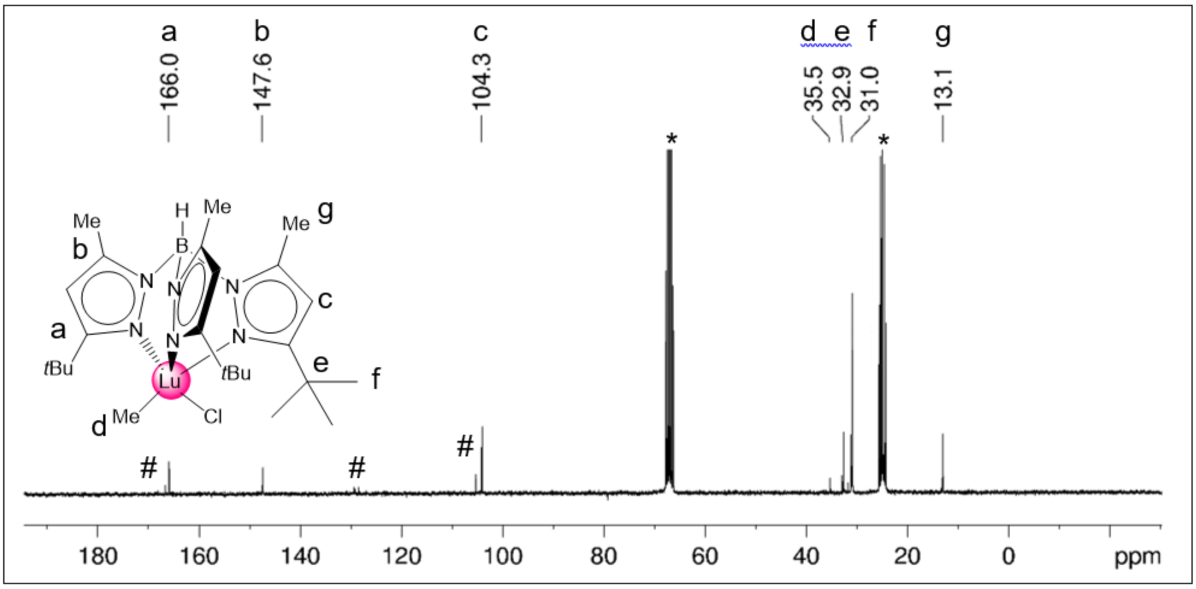


**Figure S35.** $^{13}C\{^{1}H\}$ NMR spectrum (63 MHz, $[D_8]$thf) of complex $[Tp^{tBu,Me}LuMeCl]$ (**7-Lu**) at 26°C with minor impurities (#) due to decomposition in thf.

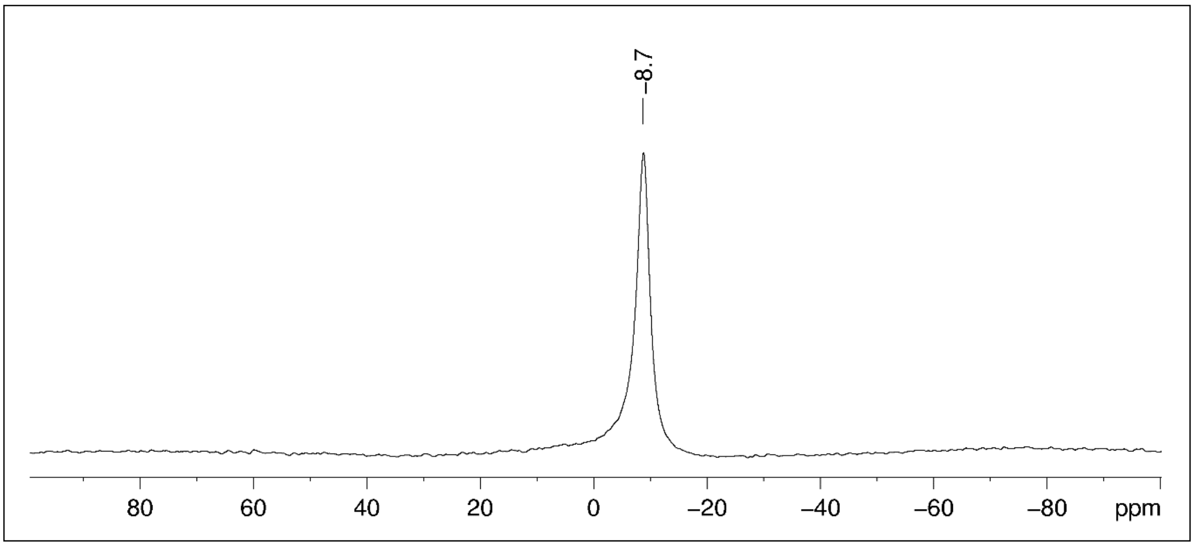


**Figure S36.** $^{11}B\{^{1}H\}$ NMR spectrum (80 MHz, $[D_8]$thf) of complex $[Tp^{tBu,Me}LuMeCl]$ (**7-Lu**) at 26 °C.

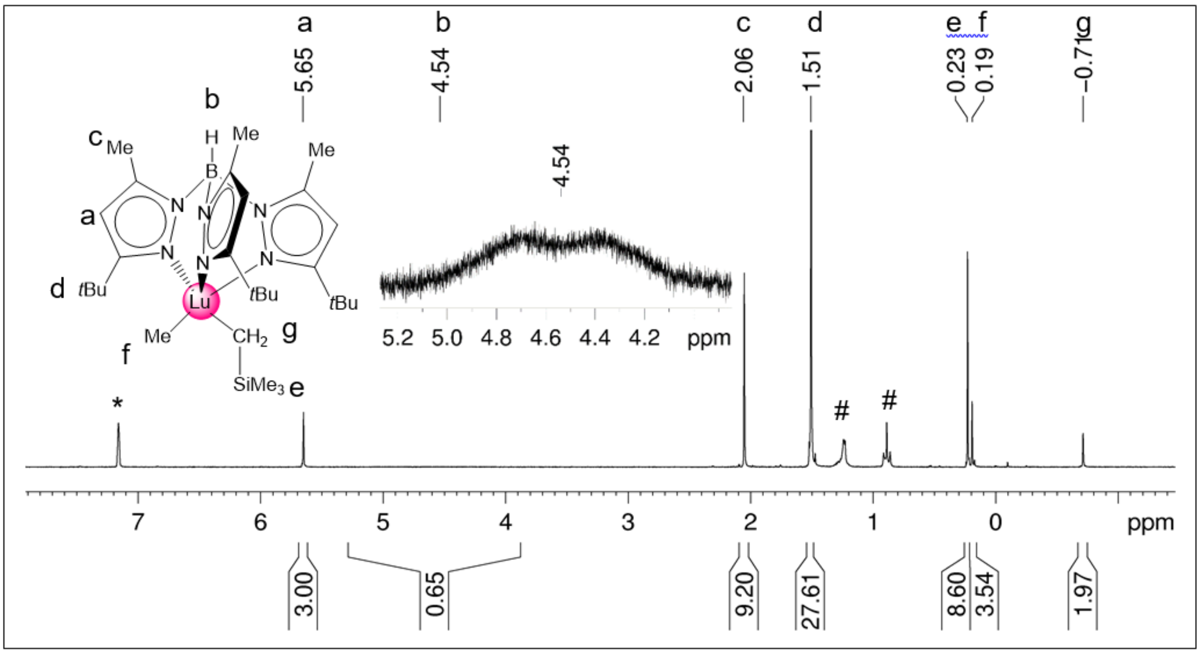


**Figure S37.** $^{1}$H NMR spectrum (250 MHz, [D$_6$]benzene) of complex [Tp$^{tBu,Me}$LuMe(CH$_2$SiMe$_3$)] (**8-Lu**) at 26 °C with traces of *n*-hexane (#).

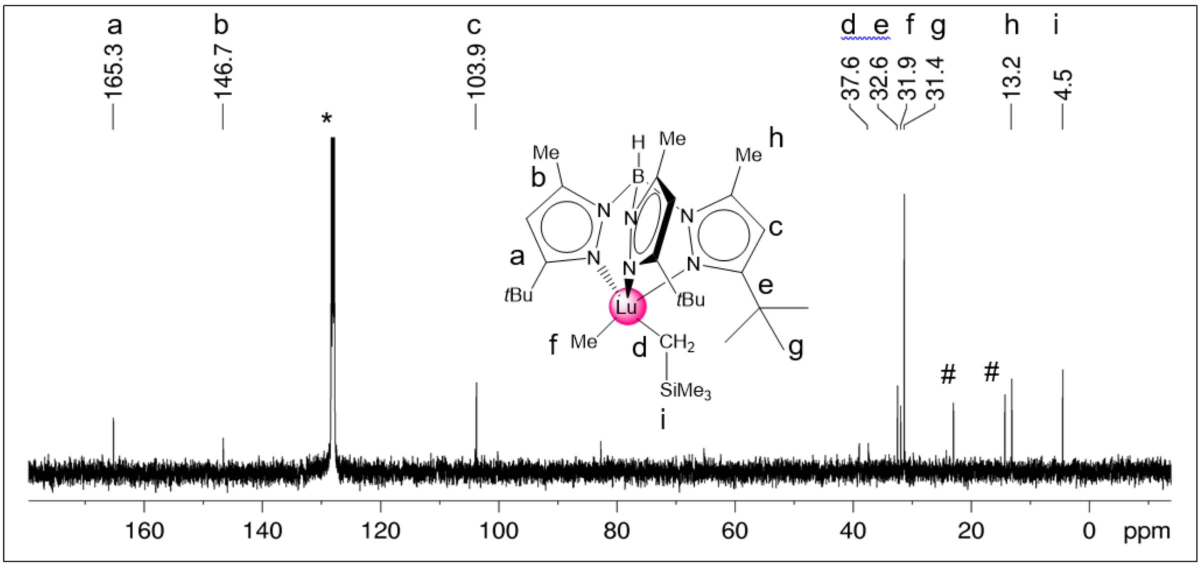


**Figure S38.** $^{13}$C{$^{1}$H} NMR spectrum (63 MHz, [D$_6$]benzene) of complex [Tp$^{tBu,Me}$LuMe(CH$_2$SiMe$_3$)] (**8-Lu**) at 26 °C with traces of *n*-hexane (#).

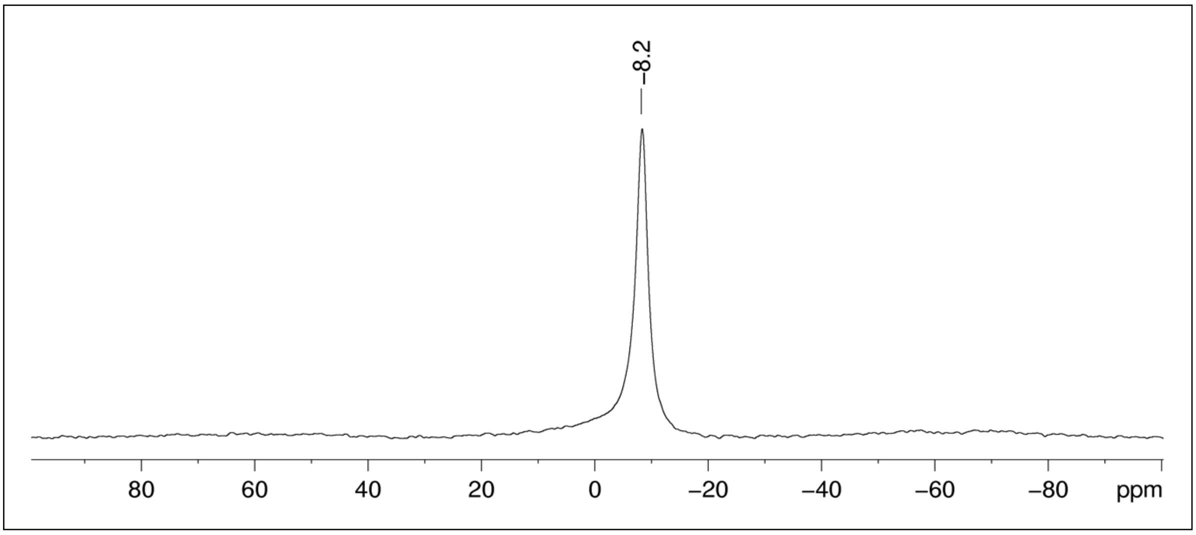


**Figure S39.** $^{11}B\{^{1}H\}$ NMR spectrum (80 MHz, [D$_6$]benzene) of complex [Tp$^{tBu,Me}$LuMe(CH$_2$SiMe$_3$)] (**8-Lu**) at 26 °C.

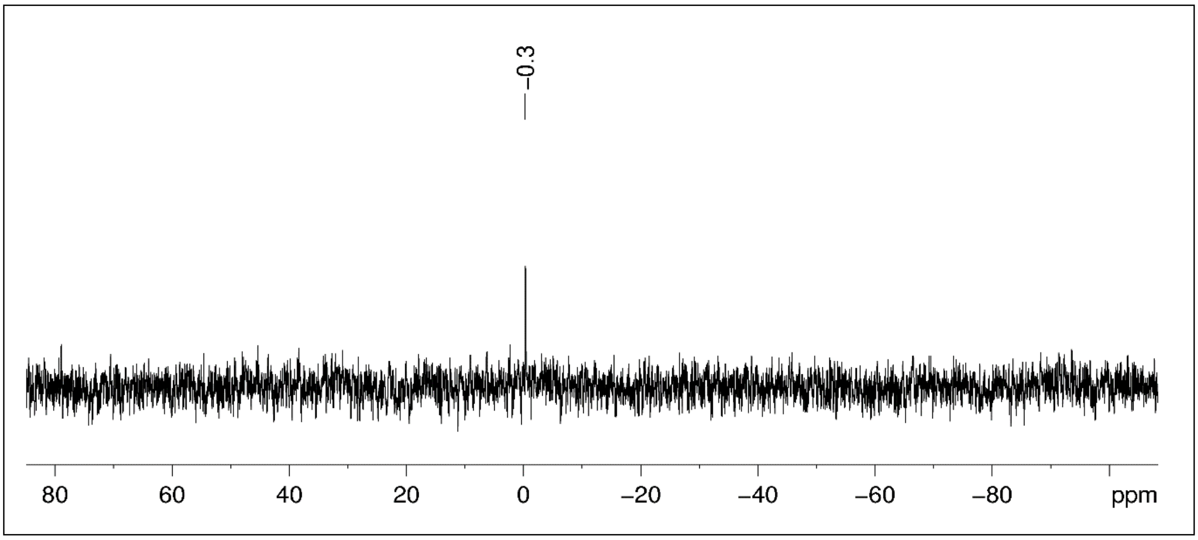


**Figure S40.** $^{29}Si\{^{1}H\}$-DEPT45 NMR spectrum (50 MHz, [D$_6$]benzene) of complex [Tp$^{tBu,Me}$LuMe(CH$_2$SiMe$_3$)] (**8-Lu**) at 26 °C.

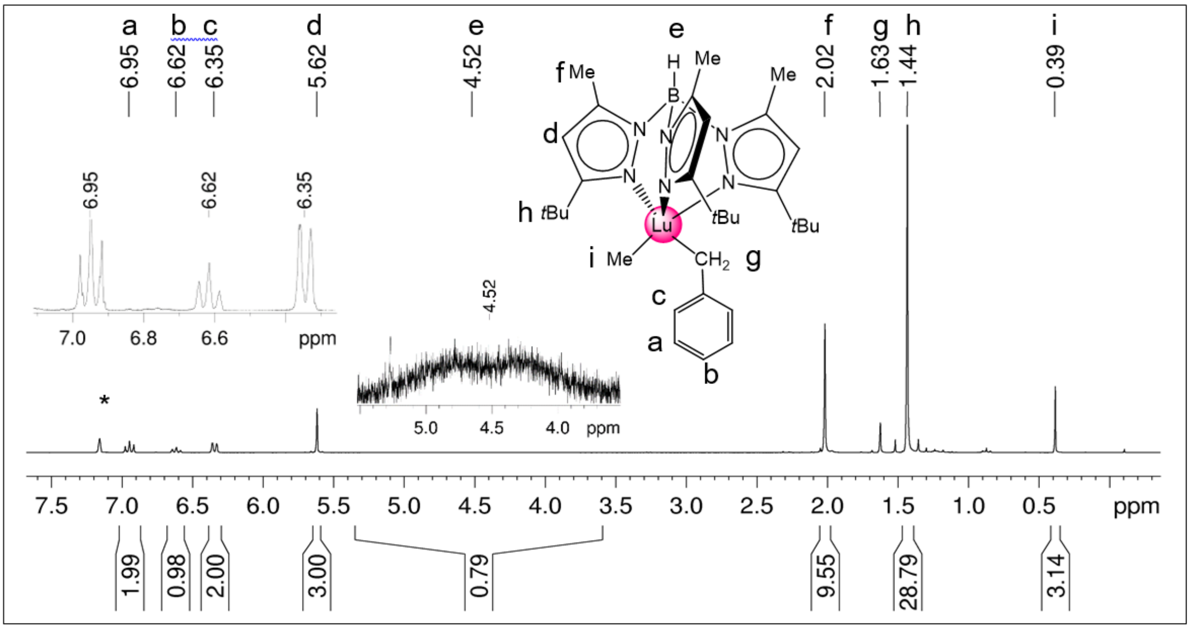


**Figure S41.** $^{1}$H NMR spectrum (250 MHz, [D6]benzene) of complex [Tp$^{tBu,Me}$LuMe(CH$_2$Ph)] (**9-Lu**) at 26 °C.

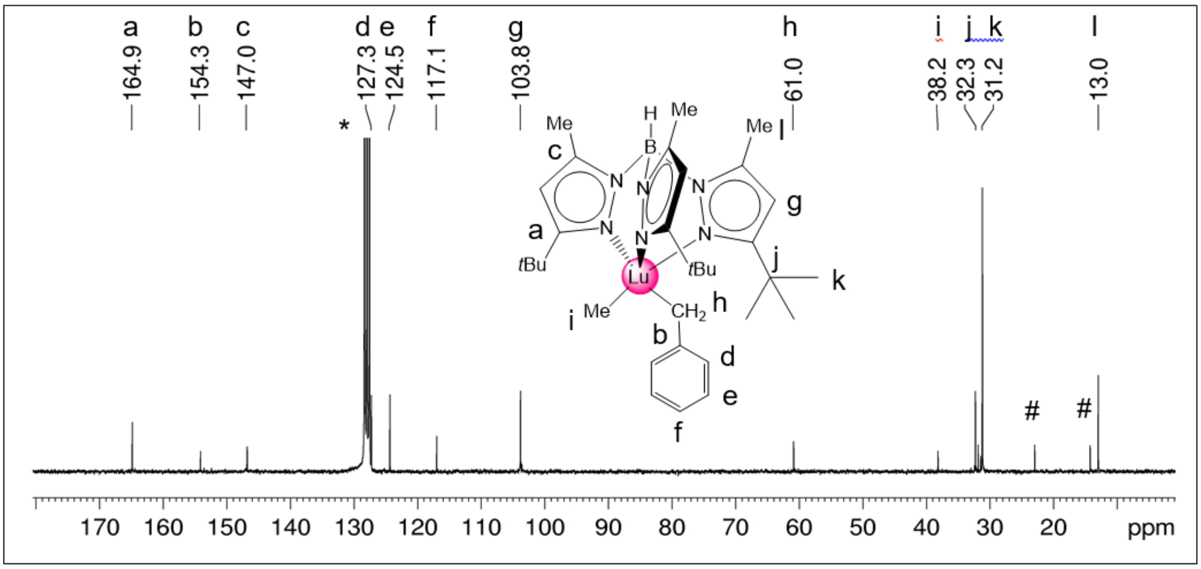


**Figure S42.** $^{13}$C{$^{1}$H} NMR spectrum (63 MHz, [D6]benzene) of complex [Tp$^{tBu,Me}$LuMe(CH$_2$Ph)] (**9-Lu**) at 26 °C with traces of hexane (#).

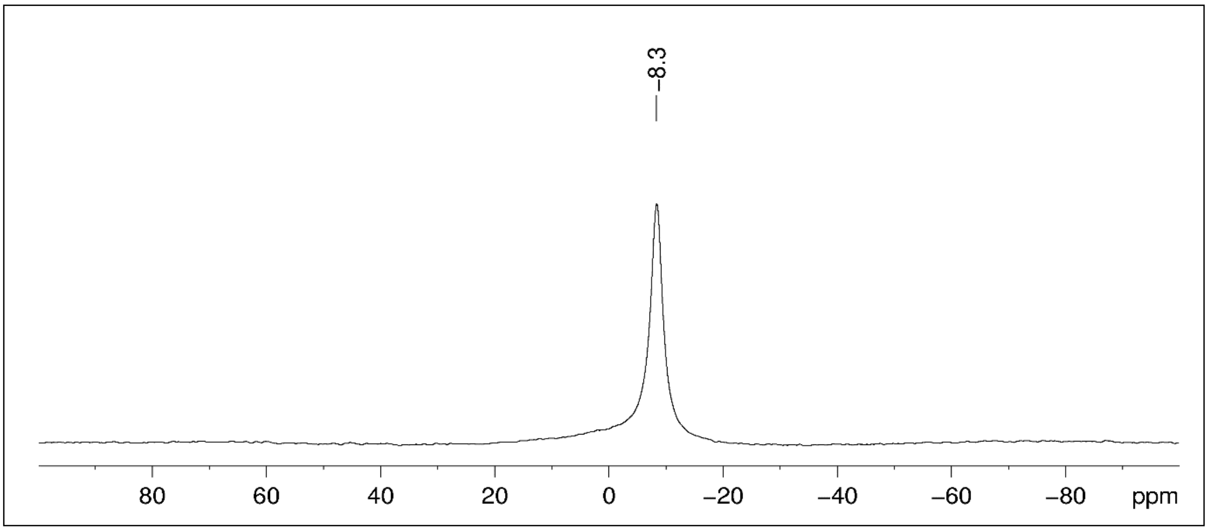


**Figure S43.** $^{11}B\{^1H\}$ NMR spectrum (80 MHz, [D$_6$]benzene) of complex [Tp$^{tBu,Me}$LuMe(CH$_2$Ph)] (**9-Lu**) at 26 °C.

## X-ray structure analyses

**Table S1.** Comprehensive crystallographic data for compounds **1-Lu**, **2-Lu**, **4-Lu**, and **5-Lu**

| | **1-Lu** | **3-Lu** | **4-Lu** | **5-Lu** |
|---|---|---|---|---|
| CCDC | 1945695 | 1945701 | 1945700 | 1945696 |
| Formula | $C_{26}H_{43}BF_3LuN_6O_3S$ | $C_{34}H_{51}BF_6LuN_7O_4S_2$ | $C_{35}H_{48}BF_{12}LuN_8O_8S_4$ | $C_{32}H_{56}BCl_2LuN_6O_2$ |
| M [g mol$^{-1}$] | 762.50 | 985.71 | 1250.83 | 813.50 |
| Crystal system | Monoclinic | Triclinic | Triclinic | Orthorhombic |
| Space group | $P2_1/n$ | $P\bar{1}$ | $P\bar{1}$ | $Cmc2_1$ |
| a [Å] | 12.2754(16) | 9.8940(3) | 12.9270(6) | 20.9267(15) |
| b [Å] | 17.658(2) | 14.1054(5) | 13.7542(6) | 9.7844(7) |
| c [Å] | 15.2272(17) | 16.1627(6) | 14.2033(7) | 17.7155(13) |
| α [°] | 90 | 86.5330(10) | 87.4410(10) | 90 |
| β [°] | 93.699(2) | 79.7220(10) | 81.360(2) | 90 |
| γ [°] | 90 | 76.3470(10) | 82.7930(10) | 90 |
| V [Å$^3$] | 3293.8(7) | 2156.34(13) | 2476.1(2) | 3627.3(5) |
| Z | 4 | 2 | 2 | 4 |
| T [K] | 100(2) | 100(2) | 100(2) | 99(2) |
| $\rho_{calcd}$ [g cm$^{-3}$] | 1.538 | 1.518 | 1.678 | 1.490 |
| μ[mm$^{-1}$] | 3.113 | 2.455 | 2.262 | 2.906 |
| F (000) | 1536 | 996 | 1252 | 1664 |
| Θ range [°] | 1.768 to 27.485 | 1.281 to 30.189 | 1.493 to 30.068 | 1.946 to 30.053 |
| total reflns | 48598 | 65970 | 106930 | 39179 |
| unique reflns | 7539 | 12746 | 14494 | 5445 |
| Rint | 0.0784 | 0.0283 | 0.0615 | 0.0544 |
| observed reflns (I>2σ) | 5801 | 11805 | 13160 | 5187 |
| Data/restraints/parameter | 7539 / 487 / 494 | 12746 / 189 / 554 | 14494 / 397 / 799 | 5445 / 61 / 235 |
| R1/wR2 (I>2σ) [a] | 0.0352 / 0.0718 | 0.0215 / 0.0505 | 0.0254 / 0.0594 | 0.0210 / 0.0469 |
| R1/wR2 (all data)[a] | 0.0569 / 0.0793 | 0.0244 / 0.0518 | 0.0299 / 0.0618 | 0.0228 / 0.0478 |
| GOF[a] | 1.046 | 1.060 | 1.053 | 1.038 |
| largest diff. peak and hole [e Å$^{-3}$] | 1.253 and -0.828 | 2.426 and -0.618 | 2.101 and -0.638 | 2.419 and -0.828 |

[a] R1 = Σ(||F0|-|Fc||)/Σ|F0|,F0> 4s(F0). wR2 = $\{\Sigma[w(F02-Fc2)^2/\Sigma[w(F02)^2]\}^{1/2}$.

**Table S1 continued.** Comprehensive crystallographic data for compounds **6-Lu**, **7-Lu**, **8-Lu**, and **9-Lu**.

| | **6-Lu** | **7-Lu** | **8-Lu** | **9-Lu** |
|---|---|---|---|---|
| CCDC | 1945699 | 1945697 | 1945698 | 1945702 |
| Formula | $C_{24}H_{40}BI_2LuN_6$ | $C_{25}H_{42}BClLuN_6$ | $C_{29}H_{54}BLuN_6Si$ | $C_{32}H_{50}BLuN_6$•1/2($C_7H_8$) |
| M [g mol$^{-1}$] | 852.20 | 647.87 | 700.65 | 750.62 |
| Crystal system | Monoclinic | Orthorhombic | Triclinic | Monoclinic |
| Space group | $P2_1/n$ | Pnma | $P\bar{1}$ | $P2_1/n$ |
| a [Å] | 12.4642(6) | 17.9200(18) | 11.9871(4) | 9.7194(7) |
| b [Å] | 13.2018(7) | 16.9031(16) | 12.1377(4) | 20.3381(15) |
| c [Å] | 19.0586(10) | 9.7340(9) | 15.4042(5) | 18.5754(14) |
| α [°] | 90 | 90 | 99.9460(10) | 90 |
| β [°] | 97.771(2) | 90 | 108.1190(10) | 98.5760(10) |
| γ [°] | 90 | 90 | 96.9700(10) | 90 |
| V [Å$^3$] | 3107.3(3) | 2948.5(5) | 2061.08(12) | 3630.8(5) |
| Z | 4 | 4 | 2 | 4 |
| T [K] | 101(2) | 173(2) | 150(2) | 100(2) |
| $\rho_{calcd}$ [g cm$^{-3}$] | 1.822 | 1.459 | 1.129 | 1.373 |
| μ[mm$^{-1}$] | 5.143 | 3.462 | 2.445 | 2.750 |
| F (000) | 1632 | 1308 | 720 | 1540 |
| Θ range [°] | 2.089 to 27.485 | 2.414 to 28.336 | 1.427 to 28.700 | 1.494 to 28.281 |
| total reflns | 78632 | 13206 | 89956 | 57404 |
| unique reflns | 7119 | 3749 | 10644 | 8999 |
| Rint | 0.0567 | | 0.0483 | 0.0563 |
| observed reflns (I>2σ) | 5912 | 2814 | 9478 | 7343 |
| Data/restraints/parameter | 7119 / 0 / 323 | 3749 / 336 / 187 | 10644 / 0 / 366 | 8999 / 159 / 483 |
| R1/wR2 (I>2σ) [a] | 0.0309 / 0.0747 | 0.0913 / 0.1642 | 0.0219 / 0.0484 | 0.0280 / 0.0605 |
| R1/wR2 (all data)[a] | 0.0407 / 0.0796 | 0.1254 / 0.1763 | 0.0274 / 0.0507 | 0.0381 / 0.0647 |
| GOF[a] | 1.038 | 1.278 | 1.059 | 1.039 |
| largest diff. peak and hole [e Å$^{-3}$] | 2.973 and -2.205 | 3.318 and -4.846 | 2.091 and -0.722 | 2.504 and -0.750 |

[a] R1 = Σ(||F0|-|Fc||)/Σ|F0|,F0> 4s(F0). wR2 = $\{\Sigma[w(F02-Fc2)_2/\Sigma[w(F02)^2]\}^{1/2}$.

Cone-angle calculations

To calculate the mathematically exact cone angles a series of .xyz files was generated from the final .cif files with ORTEP. The Mathematica package was downloaded free of charge from http://www.ccqc.uga.edu/references/software.php.

The adopted van der Waals radii were r = 1.20, 1.70, 1.55, 1.52, 1.92, 2.27 Å for H, C, N, O, B, Lu in this order.

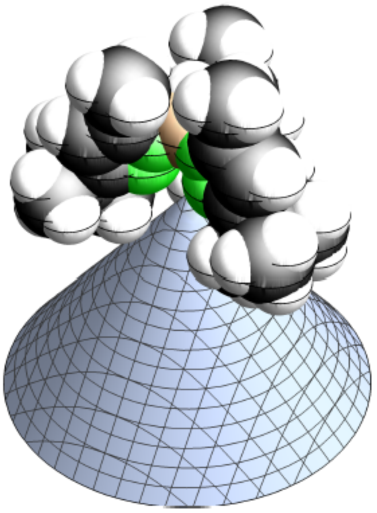

Tp$^{tBu,Me}$LuMeOTf_a (**1-Lu**)

due to disorder in one *tert*-butyl group two different cone angles were calculated for **1-Lu**

Ligand atoms forming cone = {22, 43, 49}

Cone angle (deg) = 278.007

Cone axis = {-0.316083, 0.800716, -0.508867}

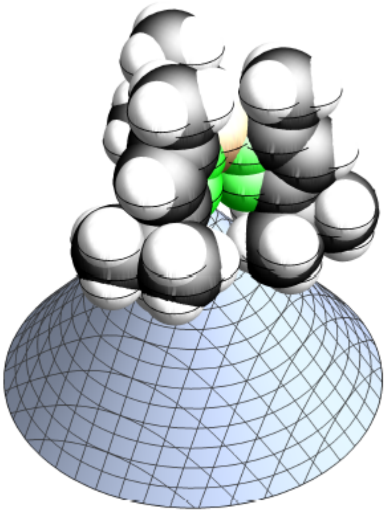

Tp$^{tBu,Me}$LuMeOTf_b (**1-Lu**)

Ligand atoms forming cone = {11, 34, 51}

Cone angle (deg) = 280.98

Cone axis = {0.162371, 0.909077, 0.383687}

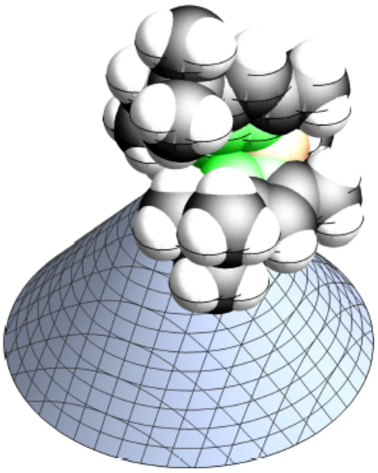

Tp$^{tBu,Me}$LuMeNTf$_2$ (**2-Lu**)

Ligand atoms forming cone = {37, 42, 53}

Cone angle (deg) = 280.4

Cone axis = {-0.755706, 0.611601, 0.234206}

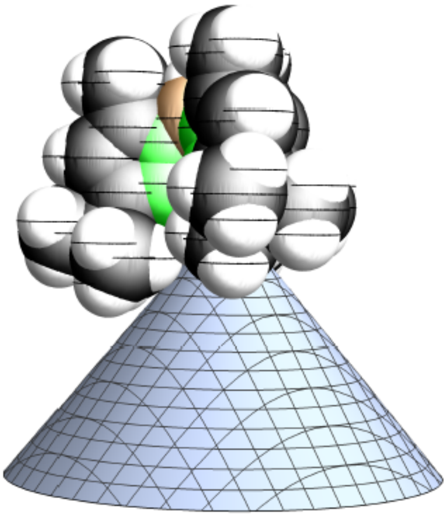

Tp$^{tBu,Me}$LuI$_2$ (**6-Lu**)

Ligand atoms forming cone = {34, 39, 46}

Cone angle (deg) = 278.17

Cone axis = {-0.143777, 0.94259, 0.301416}

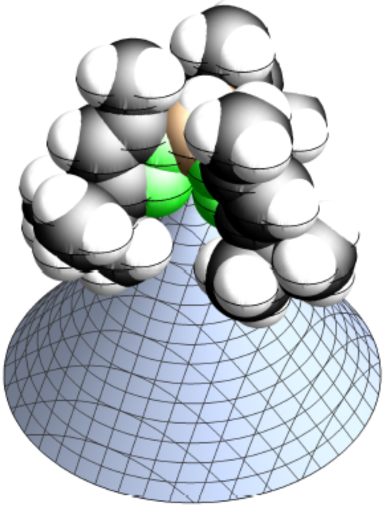

Tp$^{tBu,Me}$LuMeCl (**7-Lu**)

Ligand atoms forming cone = {35, 45, 56}

Cone angle (deg) = 278.932

Cone axis = {-0.3202, 0.943738, -0.0826429}

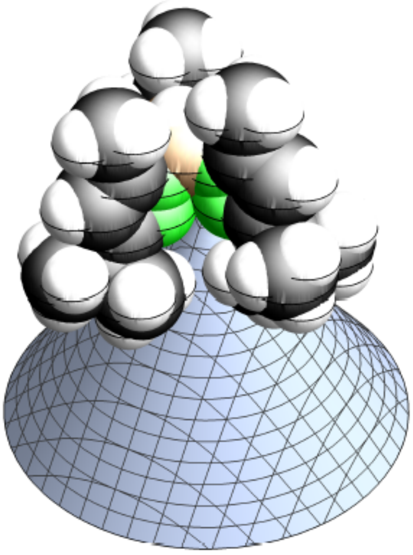

Tp$^{tBu,Me}$LuMeNeo (**8-Lu**)

Ligand atoms forming cone = {7, 31, 43}

Cone angle (deg) = 277.109

Cone axis = {-0.311522, 0.745404, 0.589344}

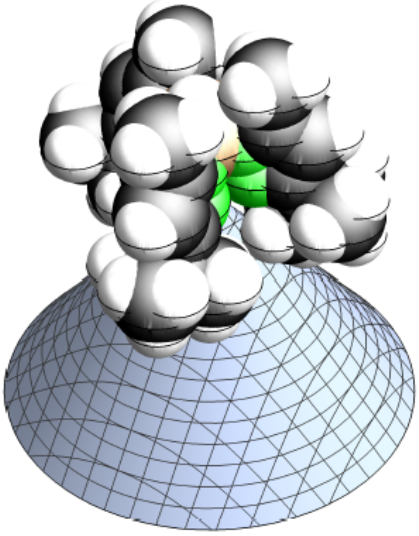

Tp$^{tBu,Me}$LuMeBn_a (**9-Lu**)

due to disorder in one *tert*-butyl group two different cone angles were calculated for **9-Lu**

Ligand atoms forming cone = {29, 42, 70}

Cone angle (deg) = 277.267

Cone axis = {-0.797352, -0.315855, 0.514262}

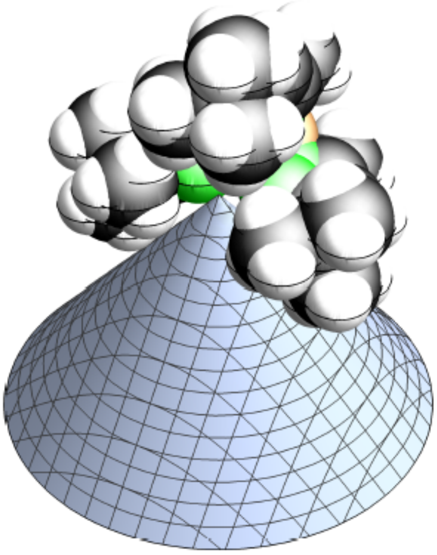

Tp$^{tBu,Me}$LuMeBn_b (**9-Lu**)

Ligand atoms forming cone = {31, 32, 67}

Cone angle (deg) = 277.268

Cone axis = {0.0836185, -0.209561, 0.974214}

Raw Data Files for Cone Angle Calculations

CompexDataBase1.txt

(X1) Tp*t*Bu,MeLuCl2

| | | | | |
|---|---|---|---|---|
| 1 | Lu | -0.36398 | -0.36470 | -1.55235 |
| 2 | C | 3.70546 | -1.32279 | -3.30348 |
| 3 | C | -1.74055 | 3.63113 | -3.19040 |
| 4 | C | 1.81816 | -2.74353 | -2.49688 |
| 5 | C | -2.95856 | 1.60157 | -2.39770 |
| 6 | C | 3.13135 | -2.07343 | -2.08815 |
| 7 | C | -2.40598 | 2.96356 | -1.97317 |
| 8 | C | 4.13539 | -3.15922 | -1.67112 |
| 9 | C | -3.57174 | 3.85151 | -1.51109 |
| 10 | C | 2.91718 | -1.12097 | -0.94626 |
| 11 | C | -1.41189 | 2.81693 | -0.85637 |
| 12 | C | -1.87025 | -3.93654 | -0.25941 |
| 13 | C | -3.74781 | -2.22864 | -0.22042 |
| 14 | C | 3.88177 | -0.59254 | -0.08289 |
| 15 | C | -0.95716 | 3.80916 | 0.01759 |
| 16 | C | -2.68715 | -2.96827 | 0.62181 |
| 17 | C | 3.21236 | 0.19854 | 0.82490 |
| 18 | C | -0.08615 | 3.19899 | 0.89339 |
| 19 | C | -1.77299 | -1.97891 | 1.30518 |
| 20 | C | -3.42012 | -3.79831 | 1.68434 |
| 21 | C | 3.76943 | 0.97563 | 1.97688 |
| 22 | C | 0.66101 | 3.80318 | 2.04143 |
| 23 | C | -1.60105 | -1.82139 | 2.68536 |
| 24 | C | -0.67632 | -0.80858 | 2.85100 |
| 25 | C | -0.13788 | -0.24560 | 4.11903 |
| 26 | H | 3.82813 | -1.95103 | -4.04757 |
| 27 | H | -2.39429 | 3.70914 | -3.91837 |
| 28 | H | 3.08412 | -0.61495 | -3.57417 |
| 29 | H | -0.98367 | 3.08528 | -3.48970 |
| 30 | H | 2.00194 | -3.44397 | -3.15734 |
| 31 | H | 4.56859 | -0.92618 | -3.06462 |
| 32 | H | -3.68801 | 1.73185 | -3.03920 |
| 33 | H | -1.42165 | 4.52279 | -2.94023 |
| 34 | H | 1.21856 | -2.07496 | -2.88783 |
| 35 | H | -2.24556 | 1.07615 | -2.81589 |
| 36 | H | 4.25469 | -3.79316 | -2.40959 |
| 37 | H | -4.23071 | 3.92551 | -2.23340 |
| 38 | H | 1.39579 | -3.14186 | -1.70845 |
| 39 | H | 4.99563 | -2.74222 | -1.45631 |
| 40 | H | -3.29733 | 1.12720 | -1.61100 |
| 41 | H | -3.23282 | 4.74273 | -1.28545 |
| 42 | H | 3.79724 | -3.63415 | -0.88444 |
| 43 | H | -1.40456 | -3.42891 | -0.95628 |
| 44 | H | -3.30225 | -1.70269 | -0.91687 |
| 45 | H | -3.99472 | 3.45373 | -0.72264 |
| 46 | H | -2.47384 | -4.58588 | -0.67771 |
| 47 | H | -4.34676 | -2.88220 | -0.63882 |
| 48 | H | 4.81893 | -0.74937 | -0.11635 |
| 49 | H | -1.20227 | 4.72776 | 0.00867 |
| 50 | H | -1.21502 | -4.40926 | 0.29465 |
| 51 | H | -4.26741 | -1.63267 | 0.35803 |
| 52 | H | -3.91096 | -4.32972 | 1.32618 |
| 53 | H | 3.55212 | 1.92460 | 1.86683 |
| 54 | H | 1.62348 | 3.67897 | 1.90688 |
| 55 | H | 4.74260 | 0.86630 | 2.00523 |

| | | | | |
|---|---|---|---|---|
| 56 | H | 0.46120 | 4.76084 | 2.09413 |
| 57 | H | -2.86954 | -4.32525 | 2.23150 |
| 58 | H | -3.98400 | -3.31149 | 2.25464 |
| 59 | H | 1.07007 | 1.12278 | 2.34745 |
| 60 | H | 3.37904 | 0.64589 | 2.81243 |
| 61 | H | 0.38856 | 3.36615 | 2.87452 |
| 62 | H | -2.03472 | -2.31382 | 3.37218 |
| 63 | H | 0.76983 | -0.43069 | 4.16441 |
| 64 | H | -0.40655 | 0.63939 | 4.18884 |
| 65 | H | -0.57345 | -0.73984 | 4.79369 |
| 66 | B | 0.71099 | 0.75168 | 1.31132 |
| 67 | N | 1.70801 | -0.65917 | -0.58415 |
| 68 | N | -0.83018 | 1.64967 | -0.53145 |
| 69 | N | 1.89421 | 0.15462 | 0.51540 |
| 70 | N | -0.01276 | 1.88928 | 0.55500 |
| 71 | N | -0.98462 | -1.09699 | 0.63789 |
| 72 | N | -0.30817 | -0.37587 | 1.62477 |
| 0 | | | | |

(X2) TptBu,MeLuI2

| | | | | |
|---|---|---|---|---|
| 1 | Lu | -0.38842 | -1.37835 | -0.71130 |
| 2 | C | -0.83145 | 0.19850 | -5.22549 |
| 3 | C | 1.58187 | 0.81917 | -4.90102 |
| 4 | C | 0.33879 | 0.22982 | -4.22287 |
| 5 | C | 0.62988 | -1.19010 | -3.76123 |
| 6 | C | -0.21596 | 2.47254 | -3.00342 |
| 7 | C | -0.00228 | 1.10591 | -3.03670 |
| 8 | C | -0.37858 | 2.83381 | -1.68175 |
| 9 | C | -0.57749 | 4.18166 | -1.08014 |
| 10 | C | 3.52965 | -2.03029 | -0.96355 |
| 11 | C | 3.56238 | -1.83145 | 0.55852 |
| 12 | C | 5.00888 | -1.54420 | 0.95763 |
| 13 | C | 2.69489 | -0.63862 | 0.94463 |
| 14 | C | 3.08385 | -3.09717 | 1.28535 |
| 15 | C | -4.34751 | -2.81399 | 1.74951 |
| 16 | C | -2.99455 | 2.60946 | 1.63065 |
| 17 | C | -2.63902 | 1.15289 | 1.62108 |
| 18 | C | -1.93485 | -3.46298 | 1.75123 |
| 19 | C | 3.04090 | 0.37594 | 1.82334 |
| 20 | C | -2.59770 | -1.04166 | 1.86204 |
| 21 | C | 1.97652 | 1.24680 | 1.85798 |
| 22 | C | -3.33443 | 0.09617 | 2.14576 |
| 23 | C | -2.94484 | -2.44683 | 2.28551 |
| 24 | C | 1.81348 | 2.48673 | 2.66340 |
| 25 | C | -2.98423 | -2.48086 | 3.82512 |
| 26 | H | -0.57565 | -0.32899 | -6.01080 |
| 27 | H | 1.85434 | 0.24086 | -5.64397 |
| 28 | H | -1.04869 | 1.11345 | -5.50230 |
| 29 | H | 1.37511 | 1.71505 | -5.24226 |
| 30 | H | -1.61472 | -0.20876 | -4.80101 |
| 31 | H | 0.92032 | -1.72966 | -4.52653 |
| 32 | H | 2.31194 | 0.87939 | -4.25013 |
| 33 | H | -0.24371 | 3.05552 | -3.75279 |
| 34 | H | -0.18149 | -1.58219 | -3.37548 |
| 35 | H | 1.33890 | -1.17456 | -3.08554 |
| 36 | H | -0.63496 | 4.85270 | -1.79219 |
| 37 | H | 3.78091 | -1.19469 | -1.40810 |
| 38 | H | 4.16145 | -2.73602 | -1.21409 |
| 39 | H | 2.62568 | -2.28735 | -1.23978 |
| 40 | H | -1.40600 | 4.18772 | -0.55725 |

| | | | | |
|---|---|---|---|---|
| 41 | H | 0.17901 | 4.39305 | -0.49445 |
| 42 | H | 5.58734 | -2.26400 | 0.62859 |
| 43 | H | 5.29191 | -0.69218 | 0.56517 |
| 44 | H | -4.34103 | -2.77876 | 0.76975 |
| 45 | H | -3.21704 | 2.89791 | 0.72056 |
| 46 | H | -1.95233 | -3.45604 | 0.77161 |
| 47 | H | 3.58306 | -3.87225 | 0.95147 |
| 48 | H | -0.30090 | 2.46917 | 0.99400 |
| 49 | H | 2.12798 | -3.22671 | 1.12089 |
| 50 | H | 5.07381 | -1.49325 | 1.93443 |
| 51 | H | -4.58251 | -3.71820 | 2.04368 |
| 52 | H | -5.00552 | -2.17488 | 2.09477 |
| 53 | H | -2.17071 | -4.35786 | 2.07425 |
| 54 | H | -2.23191 | 3.12968 | 1.96070 |
| 55 | H | 1.88814 | 3.26855 | 2.07643 |
| 56 | H | -1.03791 | -3.22799 | 2.06434 |
| 57 | H | -3.76701 | 2.75376 | 2.21629 |
| 58 | H | 3.23980 | -2.99799 | 2.24862 |
| 59 | H | 3.85358 | 0.45486 | 2.30798 |
| 60 | H | -4.15971 | 0.13640 | 2.61432 |
| 61 | H | 0.93242 | 2.48328 | 3.09244 |
| 62 | H | 2.51131 | 2.52470 | 3.34955 |
| 63 | H | -3.25702 | -3.37352 | 4.12454 |
| 64 | H | -3.62723 | -1.81692 | 4.15036 |
| 65 | H | -2.09343 | -2.27546 | 4.17931 |
| 66 | N | -0.05882 | 0.62366 | -1.77888 |
| 67 | N | -0.27531 | 1.69565 | -0.93510 |
| 68 | N | 1.44916 | -0.42312 | 0.45758 |
| 69 | N | 1.02931 | 0.76920 | 1.02222 |
| 70 | N | -1.49881 | 0.67895 | 1.06503 |
| 71 | N | -1.46917 | -0.70476 | 1.20141 |
| 72 | B | -0.26609 | 1.50754 | 0.59345 |

0

(X3) TptBu,MeLuMeCl

| | | | | |
|---|---|---|---|---|
| 1 | Lu | 1.02768 | -1.26206 | -0.37047 |
| 2 | C | 0.26377 | -1.67990 | -5.04073 |
| 3 | C | -2.08234 | -2.16579 | -4.32727 |
| 4 | C | -0.68345 | -1.76956 | -3.84333 |
| 5 | C | -1.26803 | 0.76841 | -3.65946 |
| 6 | C | -0.76941 | -0.43342 | -3.16119 |
| 7 | C | -0.18691 | -2.85014 | -2.89632 |
| 8 | C | -1.67850 | 3.08671 | -2.6156 |
| 9 | C | -1.22631 | 1.67555 | -2.63281 |
| 10 | C | 0.90228 | 4.24401 | 0.60089 |
| 11 | C | 1.50114 | 2.89863 | 0.76653 |
| 12 | C | 5.18090 | 0.52510 | 1.08772 |
| 13 | C | -1.86682 | -3.67515 | 1.16352 |
| 14 | C | 2.71415 | 2.55414 | 1.30373 |
| 15 | C | 2.79114 | 1.16324 | 1.27648 |
| 16 | C | -3.04527 | 2.16592 | 1.66406 |
| 17 | C | 3.49788 | -1.19776 | 1.69621 |
| 18 | C | -2.38643 | 0.84016 | 1.61246 |
| 19 | C | 3.86704 | 0.27103 | 1.82815 |
| 20 | C | -1.77370 | -1.29520 | 1.88913 |
| 21 | C | -2.69332 | -0.32891 | 2.27932 |
| 22 | C | -1.69019 | -2.73269 | 2.33933 |
| 23 | C | -0.36764 | -3.00287 | 3.03202 |
| 24 | C | 4.05084 | 0.58452 | 3.31679 |
| 25 | C | -2.81267 | -2.99983 | 3.33606 |

| | | | | |
|---|---|---|---|---|
| 26 | H | -0.07125 | -1.01138 | -5.67421 |
| 27 | H | 0.31675 | -2.55351 | -5.48244 |
| 28 | H | -2.40404 | -1.50422 | -4.97344 |
| 29 | H | -2.04215 | -3.04683 | -4.75379 |
| 30 | H | 1.15746 | -1.41725 | -4.73136 |
| 31 | H | -1.57722 | 0.92910 | -4.54285 |
| 32 | H | -2.69374 | -2.20165 | -3.56271 |
| 33 | H | -2.17131 | 3.28352 | -3.43900 |
| 34 | H | -0.21626 | -3.71857 | -3.34811 |
| 35 | H | 0.73479 | -2.65192 | -2.62902 |
| 36 | H | -0.89937 | 3.67872 | -2.55121 |
| 37 | H | -0.75981 | -2.87561 | -2.10113 |
| 38 | H | -2.26334 | 3.23363 | -1.84139 |
| 39 | H | 0.86460 | 4.46974 | -0.35268 |
| 40 | H | 5.06020 | 0.33286 | 0.13281 |
| 41 | H | -1.04759 | 2.43180 | -0.03442 |
| 42 | H | -1.13688 | -3.53746 | 0.52382 |
| 43 | H | -2.72367 | -3.49448 | 0.72587 |
| 44 | H | -3.41561 | 2.43404 | 0.78434 |
| 45 | H | 3.44390 | -1.43707 | 0.74747 |
| 46 | H | -0.00643 | 4.24570 | 0.97150 |
| 47 | H | 1.44831 | 4.90667 | 1.07229 |
| 48 | H | 5.44475 | 1.46216 | 1.20062 |
| 49 | H | 5.87959 | -0.05896 | 1.45079 |
| 50 | H | -1.85138 | -4.60198 | 1.48236 |
| 51 | H | 3.37658 | 3.15054 | 1.63130 |
| 52 | H | -2.43789 | 2.87248 | 2.00292 |
| 53 | H | 4.18146 | -1.74649 | 2.13297 |
| 54 | H | 2.62963 | -1.35567 | 2.12329 |
| 55 | H | -3.79887 | 2.07842 | 2.30020 |
| 56 | H | 0.36637 | -2.86336 | 2.39739 |
| 57 | H | -3.62024 | -2.83839 | 2.84777 |
| 58 | H | -3.40605 | -0.45082 | 2.89504 |
| 59 | H | 4.33207 | 1.51647 | 3.42209 |
| 60 | H | -0.35017 | -3.92879 | 3.35339 |
| 61 | H | -2.71603 | -3.92110 | 3.59000 |
| 62 | H | 4.73470 | -0.00788 | 3.69251 |
| 63 | H | 3.20315 | 0.44270 | 3.78686 |
| 64 | H | -0.26510 | -2.39198 | 3.79009 |
| 65 | H | -2.64455 | -2.40086 | 4.06382 |
| 66 | B | -0.57720 | 1.55210 | -0.09532 |
| 67 | N | -0.39221 | -0.24919 | -1.89151 |
| 68 | N | -0.67842 | 1.05475 | -1.56381 |
| 69 | N | 0.88594 | 1.75625 | 0.38592 |
| 70 | N | 1.68545 | 0.68250 | 0.69798 |
| 71 | N | -1.29094 | 0.57008 | 0.83067 |
| 72 | N | -0.89843 | -0.75071 | 0.99095 |

0

(X4) TptBu,MeLuMeNeosilyl

| | | | | |
|---|---|---|---|---|
| 1 | Lu | 0.46224 | -0.64746 | -1.51707 |
| 2 | C | -3.35919 | 0.10084 | -4.19683 |
| 3 | C | -2.39021 | -1.83409 | -2.94835 |
| 4 | C | -3.30459 | -0.62327 | -2.84211 |
| 5 | C | -4.72361 | -1.05992 | -2.46255 |
| 6 | C | 4.45556 | 2.02550 | -2.04473 |
| 7 | C | 3.56975 | -0.28826 | -1.77557 |
| 8 | C | -2.79601 | 0.34868 | -1.80106 |
| 9 | C | -3.32923 | 1.58768 | -1.46229 |
| 10 | C | 3.99212 | 0.96881 | -1.03504 |

| | | | | |
|---|---|---|---|---|
| 11 | C | -2.52385 | 2.09334 | -0.47009 |
| 12 | C | 5.14967 | 0.63262 | -0.07633 |
| 13 | C | 2.84354 | 1.52102 | -0.22582 |
| 14 | C | -0.22928 | -4.30341 | 0.22460 |
| 15 | C | -2.59833 | 3.41202 | 0.22850 |
| 16 | C | 2.82869 | 2.61478 | 0.63505 |
| 17 | C | 2.10209 | -3.51097 | 0.75721 |
| 18 | C | 1.55822 | 2.68319 | 1.15087 |
| 19 | C | 0.67915 | -3.68149 | 1.28791 |
| 20 | C | 0.12074 | -2.32206 | 1.71620 |
| 21 | C | 0.99681 | 3.65043 | 2.13989 |
| 22 | C | 0.74004 | -4.61559 | 2.50884 |
| 23 | C | -0.74711 | -0.63522 | 2.83970 |
| 24 | C | -0.38374 | -1.95640 | 2.94656 |
| 25 | C | -1.31211 | 0.26780 | 3.88838 |
| 26 | H | -3.65813 | -0.52448 | -4.8904 |
| 27 | H | -2.46766 | 0.43698 | -4.42426 |
| 28 | H | -3.98721 | 0.85139 | -4.14164 |
| 29 | H | -2.77198 | -2.47730 | -3.58184 |
| 30 | H | -5.08032 | -1.65099 | -3.15834 |
| 31 | H | -1.50741 | -1.54860 | -3.26385 |
| 32 | H | 5.20902 | 1.67402 | -2.56264 |
| 33 | H | 3.71686 | 2.24405 | -2.65089 |
| 34 | H | -5.29689 | -0.27028 | -2.37959 |
| 35 | H | 2.85682 | -0.06881 | -2.41165 |
| 36 | H | 4.33873 | -0.65557 | -2.26104 |
| 37 | H | -2.30303 | -2.25512 | -2.06714 |
| 38 | H | -4.09586 | 2.00170 | -1.84050 |
| 39 | H | 4.73537 | 2.83479 | -1.56672 |
| 40 | H | -4.70127 | -1.53883 | -1.60760 |
| 41 | H | 3.24148 | -0.95095 | -1.13256 |
| 42 | H | 5.90641 | 0.27836 | -0.58976 |
| 43 | H | -0.25298 | -3.72354 | -0.56475 |
| 44 | H | -3.34048 | 3.93582 | -0.14087 |
| 45 | H | 0.11858 | -5.18391 | -0.02692 |
| 46 | H | 2.09099 | -2.91748 | -0.02236 |
| 47 | H | -1.75747 | 3.89900 | 0.09761 |
| 48 | H | 5.42677 | 1.44317 | 0.39892 |
| 49 | H | 2.46146 | -4.38525 | 0.49803 |
| 50 | H | 4.85162 | -0.04026 | 0.57181 |
| 51 | H | -1.13551 | -4.40146 | 0.58529 |
| 52 | H | 3.55359 | 3.19861 | 0.82767 |
| 53 | H | -2.74627 | 3.26593 | 1.18558 |
| 54 | H | 2.66753 | -3.12014 | 1.45591 |
| 55 | H | 0.27390 | 4.16268 | 1.72250 |
| 56 | H | -0.97386 | 1.86021 | 1.75286 |
| 57 | H | 1.13150 | -5.47405 | 2.24533 |
| 58 | H | 1.70259 | 4.26313 | 2.43489 |
| 59 | H | -0.16549 | -4.76197 | 2.85411 |
| 60 | H | 0.64546 | 3.16107 | 2.91351 |
| 61 | H | 1.29275 | -4.20523 | 3.20731 |
| 62 | H | -2.09736 | 0.73301 | 3.53080 |
| 63 | H | -0.46427 | -2.50776 | 3.71521 |
| 64 | H | -0.63470 | 0.92539 | 4.15185 |
| 65 | H | -1.57477 | -0.26300 | 4.66897 |
| 66 | N | -1.72093 | 0.09299 | -1.04754 |
| 67 | N | 1.63132 | 0.95084 | -0.24800 |
| 68 | N | -1.55929 | 1.18349 | -0.21079 |
| 69 | N | 0.83542 | 1.67921 | 0.61171 |
| 70 | N | 0.06590 | -1.28320 | 0.85474 |
| 71 | N | -0.47281 | -0.23542 | 1.57648 |

| | | | | |
|---|---|---|---|---|
| 72 | B | -0.58097 | 1.18349 | 0.99752 |

0

(X5) TptBu,MeLuMeBn_a

| | | | | |
|---|---|---|---|---|
| 1 | Lu | 0.75370 | -1.20483 | -0.99685 |
| 2 | C | -0.42709 | 0.37569 | -5.48758 |
| 3 | C | -2.71530 | 0.12917 | -4.48492 |
| 4 | C | -1.20627 | 0.02921 | -4.20309 |
| 5 | C | -0.88723 | -1.39646 | -3.79524 |
| 6 | C | -0.99257 | 2.38458 | -3.10614 |
| 7 | C | -0.86219 | 0.99651 | -3.10777 |
| 8 | C | -0.61428 | 2.81028 | -1.85126 |
| 9 | C | -0.57877 | 4.20078 | -1.30524 |
| 10 | C | -2.70472 | -3.03753 | -0.23289 |
| 11 | C | 2.98882 | -2.66110 | 0.78567 |
| 12 | C | -2.31292 | -2.74094 | 1.23259 |
| 13 | C | 5.04238 | -1.54342 | 1.64921 |
| 14 | C | -1.96309 | -1.28599 | 1.39952 |
| 15 | C | 2.87938 | -0.28417 | 1.54343 |
| 16 | C | -1.14817 | -3.63450 | 1.64334 |
| 17 | C | 2.28000 | 1.85203 | 1.63714 |
| 18 | C | 3.51778 | -1.63264 | 1.77690 |
| 19 | C | 2.17030 | 3.31006 | 1.89780 |
| 20 | C | -1.95951 | 0.85151 | 1.95523 |
| 21 | C | 3.21342 | 0.94806 | 2.10128 |
| 22 | C | -3.32623 | -3.15371 | 2.36276 |
| 23 | C | -2.57799 | -0.34449 | 2.22715 |
| 24 | C | -2.23630 | 2.20571 | 2.52252 |
| 25 | C | 3.15455 | -2.08050 | 3.21140 |
| 26 | H | -0.67037 | -0.25470 | -6.19718 |
| 27 | H | -0.65199 | 1.28688 | -5.76924 |
| 28 | H | -2.95012 | -0.46988 | -5.22245 |
| 29 | H | 0.53456 | 0.31479 | -5.31239 |
| 30 | H | -2.94031 | 1.05145 | -4.73002 |
| 31 | H | -1.15453 | -2.00911 | -4.51312 |
| 32 | H | -1.28525 | 2.92847 | -3.82726 |
| 33 | H | -3.21512 | -0.12689 | -3.68131 |
| 34 | H | 0.07564 | -1.48218 | -3.63428 |
| 35 | H | -1.37740 | -1.61834 | -2.97582 |
| 36 | H | -0.74834 | 4.83947 | -2.02869 |
| 37 | H | 0.30311 | 4.37569 | -0.91538 |
| 38 | H | -1.93337 | -2.87832 | -0.81596 |
| 39 | H | -1.26637 | 4.29871 | -0.61420 |
| 40 | H | -3.44190 | -2.45022 | -0.49999 |
| 41 | H | -2.98640 | -3.97279 | -0.31255 |
| 42 | H | 3.16484 | -2.35356 | -0.12777 |
| 43 | H | 5.27949 | -1.29873 | 0.72976 |
| 44 | H | 3.43965 | -3.51820 | 0.93406 |
| 45 | H | -0.23440 | 2.63564 | 0.82345 |
| 46 | H | 2.02353 | -2.77316 | 0.91311 |
| 47 | H | 2.15935 | 3.79457 | 1.04676 |
| 48 | H | -0.37004 | -3.43449 | 1.08066 |
| 49 | H | -1.40108 | -4.57370 | 1.52967 |
| 50 | H | 5.44017 | -2.41148 | 1.86892 |
| 51 | H | -2.47028 | 2.82136 | 1.79689 |
| 52 | H | 5.38218 | -0.86250 | 2.26637 |
| 53 | H | -4.18434 | -2.70485 | 2.21166 |
| 54 | H | -3.45770 | -4.12521 | 2.34577 |
| 55 | H | 2.93755 | 3.60482 | 2.43181 |
| 56 | H | 1.34263 | 3.49315 | 2.38790 |

| | | | | |
|---|---|---|---|---|
| 57 | H | -0.92284 | -3.46567 | 2.58219 |
| 58 | H | 3.93973 | 1.12954 | 2.68500 |
| 59 | H | -3.27812 | -0.49878 | 2.85028 |
| 60 | H | -1.43842 | 2.53535 | 2.98607 |
| 61 | H | -2.98214 | 2.14932 | 3.15583 |
| 62 | H | -2.96816 | -2.88846 | 3.23597 |
| 63 | H | 3.54550 | -2.96249 | 3.38765 |
| 64 | H | 2.17993 | -2.13263 | 3.30093 |
| 65 | H | 3.50809 | -1.43263 | 3.85569 |
| 66 | B | -0.01389 | 1.63233 | 0.40925 |
| 67 | N | -0.40974 | 0.58652 | -1.91089 |
| 68 | N | -0.26049 | 1.71829 | -1.12944 |
| 69 | N | -1.00727 | -0.70027 | 0.65458 |
| 70 | N | 1.79812 | -0.14283 | 0.76810 |
| 71 | N | 1.41980 | 1.18259 | 0.82390 |
| 72 | N | -1.01432 | 0.63006 | 1.00957 |
| 0 | | | | |

(X6) TptBu,MeLuMeBn_b

| | | | | |
|---|---|---|---|---|
| 1 | Lu | -1.32164 | -0.78819 | -0.80604 |
| 2 | C | 1.54811 | -3.77319 | -3.69799 |
| 3 | C | 2.87928 | -1.28957 | -2.35020 |
| 4 | C | 1.28701 | -3.36590 | -2.24382 |
| 5 | C | 3.76911 | 1.10642 | -1.93064 |
| 6 | C | 1.69034 | -1.92997 | -2.00748 |
| 7 | C | -0.18643 | -3.57495 | -1.91920 |
| 8 | C | -4.38105 | 2.95038 | -1.65581 |
| 9 | C | 2.79338 | -0.00977 | -1.84167 |
| 10 | C | 2.14095 | -4.25211 | -1.30801 |
| 11 | C | 1.06267 | 4.25198 | -0.75131 |
| 12 | C | -1.47222 | 3.72197 | -0.67089 |
| 13 | C | -0.13980 | 3.37115 | -0.64592 |
| 14 | C | -2.19452 | 2.53894 | -0.52091 |
| 15 | C | -3.68147 | 2.36176 | -0.41436 |
| 16 | C | -4.05053 | 0.89656 | -0.28147 |
| 17 | C | -4.17165 | 3.10340 | 0.84114 |
| 18 | C | -0.37170 | -3.41438 | 2.19345 |
| 19 | C | 1.63161 | 0.52274 | 2.33474 |
| 20 | C | 2.76690 | 1.47442 | 2.52897 |
| 21 | C | 0.02530 | -0.97728 | 2.55255 |
| 22 | C | -2.29260 | -1.90196 | 2.88643 |
| 23 | C | -0.80646 | -2.17137 | 3.02507 |
| 24 | C | 1.08030 | -0.36202 | 3.22924 |
| 25 | C | -0.75772 | -2.15686 | 4.59825 |
| 26 | H | 0.97883 | -3.24141 | -4.29349 |
| 27 | H | 2.48954 | -3.61346 | -3.91752 |
| 28 | H | 1.34239 | -4.72423 | -3.81306 |
| 29 | H | 3.60467 | -1.66052 | -2.83742 |
| 30 | H | 4.52350 | 0.83630 | -2.49513 |
| 31 | H | -4.07077 | 2.48205 | -2.45803 |
| 32 | H | -0.72964 | -2.97281 | -2.46900 |
| 33 | H | 3.33330 | 1.89144 | -2.32174 |
| 34 | H | -0.43342 | -4.50354 | -2.11051 |
| 35 | H | -4.16667 | 3.90372 | -1.72917 |
| 36 | H | -5.35062 | 2.83973 | -1.56748 |
| 37 | H | 3.08957 | -4.13656 | -1.52388 |
| 38 | H | 1.89082 | -5.19255 | -1.42982 |
| 39 | H | 1.65866 | 3.90822 | -1.44910 |
| 40 | H | 0.78109 | 5.16178 | -0.98161 |
| 41 | H | -3.76498 | 0.41387 | -1.08511 |

| | | | | |
|---|---|---|---|---|
| 42 | H | 4.09559 | 1.32743 | -1.03436 |
| 43 | H | -0.34366 | -3.38365 | -0.97090 |
| 44 | H | -1.82861 | 4.59646 | -0.76964 |
| 45 | H | 1.98312 | -3.99129 | -0.37652 |
| 46 | H | -5.02229 | 0.81266 | -0.17766 |
| 47 | H | 1.53642 | 4.26309 | 0.10622 |
| 48 | H | 2.02832 | 1.88703 | 0.00636 |
| 49 | H | -3.60326 | 0.51793 | 0.50437 |
| 50 | H | -3.92147 | 4.04936 | 0.77891 |
| 51 | H | -5.14536 | 3.02805 | 0.90559 |
| 52 | H | -0.64679 | -3.29408 | 1.26010 |
| 53 | H | -3.75772 | 2.70728 | 1.63670 |
| 54 | H | 3.48895 | 1.25387 | 1.90435 |
| 55 | H | -2.51349 | -1.79971 | 1.93471 |
| 56 | H | 0.60153 | -3.51369 | 2.23753 |
| 57 | H | 2.45772 | 2.38904 | 2.36110 |
| 58 | H | -0.79963 | -4.21774 | 2.55867 |
| 59 | H | -2.79568 | -2.65680 | 3.25666 |
| 60 | H | -2.52363 | -1.08285 | 3.36665 |
| 61 | H | 3.09824 | 1.40564 | 3.44877 |
| 62 | H | 1.36008 | -0.52259 | 4.12268 |
| 63 | H | 0.11141 | -2.48569 | 4.90414 |
| 64 | H | -1.46911 | -2.73137 | 4.95366 |
| 65 | H | -0.89236 | -1.23873 | 4.92321 |
| 66 | B | 1.19540 | 1.17374 | -0.14942 |
| 67 | N | 0.90866 | -1.08268 | -1.32828 |
| 68 | N | 1.58981 | 0.11204 | -1.22037 |
| 69 | N | -0.06552 | 2.02553 | -0.49625 |
| 70 | N | -1.34439 | 1.50314 | -0.42227 |
| 71 | N | 0.93707 | 0.43437 | 1.17436 |
| 72 | N | -0.06819 | -0.49835 | 1.29815 |

0

(X7) TptBu,MeLuMeNTf2

| | | | | |
|---|---|---|---|---|
| 1 | Lu | 0.90235 | -1.28354 | -0.05014 |
| 2 | C | -2.36375 | -2.92884 | -3.75077 |
| 3 | C | 3.27280 | 2.39663 | -3.39047 |
| 4 | C | 2.68936 | -0.02325 | -3.25201 |
| 5 | C | -0.19201 | -3.32403 | -2.54801 |
| 6 | C | -1.70805 | -3.24733 | -2.39554 |
| 7 | C | 2.80474 | 1.28131 | -2.44158 |
| 8 | C | -2.24162 | -4.59637 | -1.90919 |
| 9 | C | 0.75835 | 2.84340 | -2.00323 |
| 10 | C | 1.47195 | 1.65660 | -1.84288 |
| 11 | C | -2.10217 | -2.16580 | -1.41904 |
| 12 | C | 3.84135 | 1.12238 | -1.32207 |
| 13 | C | -0.37014 | 2.72123 | -1.21438 |
| 14 | C | -1.46400 | 3.72260 | -1.00289 |
| 15 | C | -3.37656 | -1.86076 | -0.95704 |
| 16 | C | -3.23980 | -0.75061 | -0.15197 |
| 17 | C | -4.29659 | -0.00718 | 0.59660 |
| 18 | C | 2.89160 | -1.52823 | 2.56317 |
| 19 | C | -0.80501 | 1.27454 | 2.97246 |
| 20 | C | 1.09274 | 0.12902 | 3.08339 |
| 21 | C | -1.99649 | 2.12418 | 3.27115 |
| 22 | C | 3.44028 | 0.69653 | 3.52674 |
| 23 | C | 2.42658 | -0.43823 | 3.50951 |
| 24 | C | 0.22892 | 0.92803 | 3.82141 |
| 25 | C | 2.28893 | -1.02597 | 4.91998 |
| 26 | H | -2.17849 | -3.65482 | -4.38255 |

| | | | | |
|---|---|---|---|---|
| 27 | H | 2.63743 | 2.48398 | -4.13109 |
| 28 | H | -1.99846 | -2.08949 | -4.10103 |
| 29 | H | 2.06166 | 0.10746 | -3.99290 |
| 30 | H | 4.15854 | 2.17272 | -3.74544 |
| 31 | H | -3.33184 | -2.83896 | -3.63142 |
| 32 | H | 3.56938 | -0.26505 | -3.60825 |
| 33 | H | 0.03465 | -3.99443 | -3.22613 |
| 34 | H | 3.32227 | 3.24379 | -2.89970 |
| 35 | H | -2.00226 | -5.29378 | -2.55455 |
| 36 | H | 0.15312 | -2.44907 | -2.82483 |
| 37 | H | 2.36251 | -0.74065 | -2.67105 |
| 38 | H | 0.99863 | 3.58599 | -2.54450 |
| 39 | H | -3.21701 | -4.55060 | -1.82427 |
| 40 | H | 4.71359 | 0.90239 | -1.71217 |
| 41 | H | -1.33894 | 4.47819 | -1.61524 |
| 42 | H | 0.20974 | -3.57646 | -1.69179 |
| 43 | H | -4.18116 | -2.32474 | -1.15585 |
| 44 | H | -2.33131 | 3.30110 | -1.17707 |
| 45 | H | -1.84678 | -4.80851 | -1.03745 |
| 46 | H | 3.91045 | 1.96001 | -0.81929 |
| 47 | H | 3.56320 | 0.40185 | -0.71902 |
| 48 | H | -1.43832 | 4.04436 | -0.07799 |
| 49 | H | -4.30219 | 0.93013 | 0.30895 |
| 50 | H | -5.17145 | -0.40934 | 0.41318 |
| 51 | H | -2.13642 | 1.65421 | 0.59397 |
| 52 | H | -4.11000 | -0.05279 | 1.55717 |
| 53 | H | 3.02639 | -1.14987 | 1.66896 |
| 54 | H | 2.21351 | -2.23478 | 2.51924 |
| 55 | H | 3.50475 | 1.09050 | 2.63120 |
| 56 | H | -1.99349 | 2.90988 | 2.68529 |
| 57 | H | 3.73587 | -1.90540 | 2.88867 |
| 58 | H | -2.81484 | 1.60593 | 3.11790 |
| 59 | H | 4.31655 | 0.34853 | 3.79457 |
| 60 | H | 3.15266 | 1.38280 | 4.16487 |
| 61 | H | -1.96471 | 2.41537 | 4.20582 |
| 62 | H | 0.32879 | 1.18512 | 4.73035 |
| 63 | H | 1.59110 | -1.71542 | 4.92019 |
| 64 | H | 3.14151 | -1.42313 | 5.19307 |
| 65 | H | 2.04461 | -0.31347 | 5.54750 |
| 66 | N | 0.81501 | 0.84243 | -0.99506 |
| 67 | N | -1.21870 | -1.28777 | -0.91280 |
| 68 | N | -0.33411 | 1.51721 | -0.60733 |
| 69 | N | -1.93500 | -0.39641 | -0.13309 |
| 70 | N | -0.57211 | 0.70094 | 1.77351 |
| 71 | N | 0.60878 | -0.02060 | 1.83609 |
| 72 | B | -1.31765 | 0.92791 | 0.42548 |
| 0 | | | | |

(X8) TptBu,MeLuMeOTf_a

| | | | | |
|---|---|---|---|---|
| 1 | Lu | -0.13770 | -1.42063 | 0.81601 |
| 2 | C | 0.48602 | -3.42979 | -3.97813 |
| 3 | C | -2.36658 | 1.25386 | -3.42774 |
| 4 | C | -1.46736 | -1.19358 | -3.45362 |
| 5 | C | -1.56379 | -4.27741 | -2.83642 |
| 6 | C | -1.60676 | 0.05265 | -2.90179 |
| 7 | C | -0.32408 | -3.41136 | -2.60896 |
| 8 | C | -0.78218 | -1.95521 | -2.52246 |
| 9 | C | 1.62271 | 3.20566 | -1.90035 |
| 10 | C | 0.39127 | -3.81291 | -1.44354 |
| 11 | C | 1.87938 | 2.05036 | -0.98134 |

| | | | | |
|---|---|---|---|---|
| 12 | C | 3.09091 | 1.61239 | -0.46066 |
| 13 | C | -2.56508 | 3.57267 | 0.05492 |
| 14 | C | 3.78819 | -1.82002 | 0.35006 |
| 15 | C | 2.80162 | 0.46616 | 0.27385 |
| 16 | C | 5.18377 | 0.14246 | 0.94214 |
| 17 | C | -2.13934 | 2.43324 | 0.93046 |
| 18 | C | 3.76268 | -0.42662 | 1.01928 |
| 19 | C | -2.42844 | 2.21787 | 2.25878 |
| 20 | C | 3.34680 | -0.55801 | 2.48596 |
| 21 | C | -1.79891 | 1.03497 | 2.59609 |
| 22 | C | -0.99960 | -0.98085 | 3.85627 |
| 23 | C | -1.74906 | 0.35544 | 3.93997 |
| 24 | C | -3.19013 | 0.12031 | 4.43249 |
| 25 | C | -1.03847 | 1.27393 | 4.93241 |
| 26 | H | -0.10255 | -3.15508 | -4.71090 |
| 27 | H | -2.70112 | 1.06060 | -4.32863 |
| 28 | H | 0.81836 | -4.33625 | -4.14776 |
| 29 | H | -1.77805 | -1.47809 | -4.30453 |
| 30 | H | 1.24283 | -2.81037 | -3.91727 |
| 31 | H | -2.03820 | -3.96541 | -3.63588 |
| 32 | H | -1.76945 | 2.02957 | -3.46072 |
| 33 | H | -1.29144 | -5.20947 | -2.96033 |
| 34 | H | -3.12133 | 1.44775 | -2.83407 |
| 35 | H | 1.28861 | 2.87463 | -2.76007 |
| 36 | H | -2.15607 | -4.21010 | -2.05916 |
| 37 | H | 2.45561 | 3.70127 | -2.04344 |
| 38 | H | 0.73500 | -4.72222 | -1.56901 |
| 39 | H | 0.95496 | 3.80097 | -1.49859 |
| 40 | H | -0.77218 | 2.19276 | -1.46611 |
| 41 | H | 1.13990 | -3.19893 | -1.29232 |
| 42 | H | -3.02748 | 3.22329 | -0.73544 |
| 43 | H | -0.20792 | -3.79488 | -0.66899 |
| 44 | H | 4.00173 | -1.72309 | -0.60112 |
| 45 | H | 3.94431 | 2.01232 | -0.58165 |
| 46 | H | -1.77596 | 4.08095 | -0.22841 |
| 47 | H | 5.49263 | 0.12598 | 0.01424 |
| 48 | H | -3.17099 | 4.15994 | 0.55239 |
| 49 | H | 2.90950 | -2.24417 | 0.44536 |
| 50 | H | 4.46782 | -2.37775 | 0.78319 |
| 51 | H | 5.18380 | 1.06493 | 1.27131 |
| 52 | H | 5.78207 | -0.40093 | 1.49687 |
| 53 | H | 2.46526 | -0.98210 | 2.53946 |
| 54 | H | -2.95229 | 2.76911 | 2.82692 |
| 55 | H | 4.00235 | -1.10832 | 2.96523 |
| 56 | H | 3.30699 | 0.33151 | 2.89571 |
| 57 | H | -1.46975 | -1.58002 | 3.23934 |
| 58 | H | -0.08858 | -0.82425 | 3.52993 |
| 59 | H | -3.63933 | -0.51731 | 3.83860 |
| 60 | H | -3.68047 | 0.96997 | 4.42748 |
| 61 | H | -0.11450 | 1.42083 | 4.63804 |
| 62 | H | -0.96254 | -1.38987 | 4.74617 |
| 63 | H | -1.50690 | 2.13265 | 4.97586 |
| 64 | H | -3.17029 | -0.23952 | 5.34455 |
| 65 | H | -1.03490 | 0.85720 | 5.81869 |
| 66 | N | -1.00374 | 0.05226 | -1.70556 |
| 67 | N | -0.48903 | -1.19843 | -1.45596 |
| 68 | N | 0.92497 | 1.20904 | -0.56278 |
| 69 | N | 1.47504 | 0.21427 | 0.20593 |
| 70 | N | -1.36521 | 1.40146 | 0.47196 |
| 71 | N | -1.14334 | 0.52069 | 1.52359 |
| 72 | B | -0.59456 | 1.29512 | -0.85083 |

0

(X9) TptBu,MeLuMeOTf_b

| | | | | |
|---|---|---|---|---|
| 1 | Lu | -0.10666 | -1.62460 | 0.07682 |
| 2 | C | -1.39766 | 0.01065 | -5.06482 |
| 3 | C | 0.12502 | -1.57515 | -3.87519 |
| 4 | C | -1.05311 | -0.61319 | -3.70783 |
| 5 | C | -2.30249 | -1.38602 | -3.22631 |
| 6 | C | -0.76229 | 1.84475 | -2.88743 |
| 7 | C | -0.71862 | 0.46538 | -2.70724 |
| 8 | C | -0.40267 | 2.40271 | -1.66674 |
| 9 | C | 4.73353 | -1.77658 | -1.17468 |
| 10 | C | -0.34866 | 3.85012 | -1.28301 |
| 11 | C | 2.71779 | -3.03116 | -0.39592 |
| 12 | C | 3.80974 | -2.03432 | 0.01431 |
| 13 | C | -4.40588 | -1.18231 | 0.53469 |
| 14 | C | 3.21050 | -0.72536 | 0.45905 |
| 15 | C | 3.86143 | 0.39001 | 0.95056 |
| 16 | C | 4.64641 | -2.61675 | 1.16987 |
| 17 | C | 2.88276 | 1.33388 | 1.16437 |
| 18 | C | 3.03073 | 2.73064 | 1.68715 |
| 19 | C | -2.92384 | -2.82177 | 1.82192 |
| 20 | C | -3.60804 | -1.36412 | 1.92092 |
| 21 | C | -2.57137 | -0.24717 | 2.04519 |
| 22 | C | -1.42678 | 1.55700 | 2.58391 |
| 23 | C | -4.63938 | -1.36210 | 2.99911 |
| 24 | C | -2.55866 | 0.84876 | 2.89098 |
| 25 | C | -0.84986 | 2.78288 | 3.26305 |
| 26 | H | -1.53278 | -0.70133 | -5.72496 |
| 27 | H | -0.66107 | 0.58735 | -5.35489 |
| 28 | H | -2.21560 | 0.54065 | -4.98278 |
| 29 | H | -0.09304 | -2.24612 | -4.55677 |
| 30 | H | 0.91961 | -1.07385 | -4.15454 |
| 31 | H | -2.56840 | -2.03540 | -3.91053 |
| 32 | H | -0.99268 | 2.30917 | -3.68374 |
| 33 | H | -3.03677 | -0.75689 | -3.06899 |
| 34 | H | 0.30472 | -2.02393 | -3.02306 |
| 35 | H | -2.09336 | -1.85803 | -2.39290 |
| 36 | H | -0.53530 | 4.40424 | -2.06910 |
| 37 | H | 4.21072 | -1.41831 | -1.92319 |
| 38 | H | 5.15783 | -2.61621 | -1.44700 |
| 39 | H | 5.42215 | -1.12917 | -0.91899 |
| 40 | H | 2.18216 | -2.64801 | -1.12206 |
| 41 | H | 0.54439 | 4.06405 | -0.93893 |
| 42 | H | 3.13490 | -3.86384 | -0.70133 |
| 43 | H | -1.01697 | 4.03217 | -0.58980 |
| 44 | H | -3.79871 | -1.35480 | -0.21560 |
| 45 | H | -5.15270 | -1.81525 | 0.50272 |
| 46 | H | -4.74945 | -0.26635 | 0.47320 |
| 47 | H | 2.14027 | -3.21688 | 0.37389 |
| 48 | H | 5.11336 | -3.42260 | 0.86281 |
| 49 | H | 2.76639 | 3.37029 | 0.99267 |
| 50 | H | 4.79264 | 0.48572 | 1.10783 |
| 51 | H | -2.42316 | -2.89077 | 0.98104 |
| 52 | H | 0.42605 | 2.58406 | 0.89574 |
| 53 | H | 5.30380 | -1.95141 | 1.46572 |
| 54 | H | -3.61915 | -3.51190 | 1.84578 |
| 55 | H | 4.05638 | -2.84544 | 1.91861 |
| 56 | H | 3.96379 | 2.88920 | 1.93967 |
| 57 | H | 2.45700 | 2.84868 | 2.47293 |

| | | | | |
|---|---|---|---|---|
| 58 | H | -5.29957 | -2.06262 | 2.82026 |
| 59 | H | -2.31168 | -2.94870 | 2.57598 |
| 60 | H | -0.70692 | 3.48797 | 2.59838 |
| 61 | H | -5.08645 | -0.49005 | 3.02155 |
| 62 | H | -3.20531 | 1.06609 | 3.55139 |
| 63 | H | -4.20900 | -1.53011 | 3.86419 |
| 64 | H | 0.00463 | 2.55143 | 3.68254 |
| 65 | H | -1.47418 | 3.10159 | 3.94827 |
| 66 | N | -0.34954 | 0.19180 | -1.43571 |
| 67 | N | -0.14902 | 1.39969 | -0.81615 |
| 68 | N | 1.87445 | -0.49793 | 0.37100 |
| 69 | N | 1.66931 | 0.79997 | 0.82296 |
| 70 | N | -1.50578 | -0.19695 | 1.23397 |
| 71 | N | -0.80213 | 0.93422 | 1.57567 |
| 72 | B | 0.32080 | 1.51305 | 0.65551 |

0
